# Diabetes and Obesity and Treatment Effect of Early Rhythm Control vs Usual Care in Patients With Atrial Fibrillation: A Secondary Analysis of the EAST-AFNET 4 Randomized Clinical Trial

**DOI:** 10.1001/jamacardio.2025.2374

**Published:** 2025-07-30

**Authors:** Andreas Metzner, Stephan Willems, Katrin Borof, Guenther Breithardt, A. John Camm, Harry J. G. M. Crijns, Lars Eckardt, Larissa Fabritz, Nele Gessler, Andreas Goette, Bruno Reissmann, Renate B. Schnabel, Ulrich Schotten, Antonia Zapf, Andreas Rillig, Paulus Kirchhof

**Affiliations:** 1 Department of Cardiology, University Heart and Vascular Center, University Medical Center Hamburg-Eppendorf, Hamburg, Germany; 2 German Center for Cardiovascular Research, Partner Site Hamburg/Lübeck/Kiel, Hamburg, Germany; 3 Department of Cardiology and Internal Intensive Care Medicine, Asklepios Hospital St Georg, Hamburg, Germany; 4 Atrial Fibrillation Network (AFNET), Münster, Germany; 5 Department of Cardiology II (Electrophysiology), University Hospital Münster, Münster, Germany; 6 Cardiology Clinical Academic Group, Molecular and Clinical Sciences Research Institute, St George’s University of London, London, United Kingdom; 7 Department of Cardiology, Maastricht University Medical Center and Cardiovascular Research Institute Maastricht, Maastricht, the Netherlands; 8 Institute of Medical Biometry and Epidemiology, University Medical Center Hamburg-Eppendorf, Hamburg, Germany; 9 Institute of Cardiovascular Sciences, University of Birmingham, Birmingham, United Kingdom; 10 St Vincenz Hospital, Paderborn, Germany

## Abstract

**Importance:**

The EAST-AFNET 4 randomized clinical trial demonstrated that early rhythm control therapy added to anticoagulation therapy and therapy of concomitant conditions reduces the primary composite outcome of cardiovascular death, stroke, hospitalization because of heart failure, or acute coronary syndrome compared to usual care. However, the impact of body mass index (BMI, calculated as weight in kilograms divided by height in meters squared) and diabetes on outcomes in EAST-AFNET 4 is not known.

**Objective:**

To assess the effects of BMI and diabetes on outcomes in EAST-AFNET 4.

**Design, Setting, and Participants:**

EAST-AFNET 4 is an international, investigator-initiated, parallel-group, open, blinded outcome assessment randomized clinical trial conducted in 11 European countries. Patients who had early atrial fibrillation (AF, diagnosed ≤1 year before enrollment) and cardiovascular conditions were eligible for inclusion. The current analysis is a prespecified secondary analysis of the EAST-AFNET 4 trial performed in the final, locked dataset assigning patients to therapy group on the basis of randomization (intention-to-treat population). EAST-AFNET 4 was conducted from June 2010 to May 2020, and this secondary analysis of the final locked data base was performed in 2024.

**Intervention:**

EAST-AFNET 4 randomly assigned patients to either early rhythm control or usual care.

**Main Outcomes and Measure:**

The primary outcome of this analysis and the EAST-AFNET 4 trial is a composite of cardiovascular death, stroke, hospitalization because of heart failure, or acute coronary syndrome.

**Results:**

There were 1086 patients with obesity (BMI ≥30; mean [SD] BMI 34.5 [4.2]) and 1690 patients without obesity (BMI <30; mean [SD] BMI 25.9 [2.6]). Overall mean patient age was 70 years, and 1293 patients (46.6%) were female. Patients with obesity were younger (mean [SD] age, 68 [8.6] vs 72 [7.7] years) and had more frequently nonparoxysmal AF patterns (31% vs 24%) than patients without obesity. There was no difference in mean (SD) CHA_2_DS_2_-VASc score (3.4 [1.3] vs 3.3 [1.3]). Obesity did not change the effect of early rhythm control therapy on the first primary outcome (hazard rate point estimates: BMI <30, 0.84; BMI ≥30, 0.69; *P* for interaction = .22). Patients with diabetes were younger (mean [SD] age, 69 [8.6] vs 71 [8.2] years; *P* = .001) and had a higher mean CHA_2_DS_2_-VASC score (4.06 vs 3.11; *P* < .001). Diabetes did not interact with the treatment effect of early rhythm control (diabetes: hazard ratio [HR], 0.77; 95% CI, 0.57-1.05 vs no diabetes: HR, 0.78; 95% CI, 0.64-0.96; *P* for interaction = .93). There was no difference in safety outcomes between patients with and without diabetes (64 of 351 patients [18.2%] vs 167 of 1039 patients [16.1%]; *P* for interaction = .99).

**Conclusions and Relevance:**

This secondary analysis of the EAST-AFNET 4 randomized clinical trial shows that early rhythm control therapy retains its effectiveness and safety in patients with and without diabetes and patients with and without obesity.

**Trial Registration:**

ClinicalTrials.gov Identifier: NCT01288352

## Key Points

**Question:**

What are the impacts of obesity and diabetes on the effectiveness and safety of early rhythm control in patients with early atrial fibrillation and cardiovascular conditions?

**Findings:**

In this prespecified secondary analysis of the EAST-AFNET 4 randomized clinical trial involving 1086 patients with obesity and 1690 without, obesity did not change the effect of early rhythm control therapy. In 694 patients with diabetes, there was no interaction with the treatment effect of early rhythm control.

**Meaning:**

Early rhythm control therapy retains its effectiveness and safety in patients with and without diabetes and in patients with and without obesity.

## Teaser

This secondary analysis of the EAST-AFNET 4 randomized clinical trial assesses the effects of body mass index and diabetes on outcomes in EAST-AFNET 4.

## Introduction

Atrial fibrillation (AF) is the most frequent arrhythmia and is associated with increased morbidity and mortality.^1,2^ Obesity increases AF recurrences, most likely due to direct and indirect effects of epicardial fat on atrial function and structure, as well as electrical and structural atrial remodeling.^3,4,5,6^ The cardiometabolic determinants of AF^7,8,9^ furthermore suggest that patients with metabolic defects due to diabetes^6^ may be less suitable for rhythm control therapy. The EAST-AFNET 4 randomized clinical trial demonstrated that early rhythm control (ERC) therapy, when added to anticoagulation therapy and therapy of concomitant conditions, further reduces a combined outcome of cardiovascular death, stroke, hospitalization because of heart failure, or acute coronary syndrome compared to usual care (NCT01288352).^10^ The effect of ERC therapy is independent of AF-related symptoms^11^ and mediated by sinus rhythm.^12^ Whether this effectiveness is retained in patients with obesity and in those with diabetes is not known. The current subanalysis of the EAST-AFNET 4 study therefore assesses the effects of body mass index (BMI, calculated as weight in kilograms divided by height in meters squared) and of diabetes on outcomes in EAST-AFNET 4.

## Methods

This is a prespecified secondary analysis of the EAST-AFNET 4 trial. In brief, EAST-AFNET 4 was an international, investigator initiated, parallel-group, open, blinded outcome assessment randomized clinical trial conducted in 11 European countries. A total of 2789 patients with AF diagnosed within 12 months and at least 2 CHA_2_DS_2_-VASc risk factors were randomized to either ERC therapy (n = 1395) or usual care (UC; n = 1394). ERC consisted of anti-arrhythmic drug therapy, catheter ablation, or cardioversion in all patients after randomization. In patients assigned to UC, rate control was the initial strategy, and rhythm control was used in patients who remained symptomatic on optimal rate control therapy. Anticoagulation therapy and treatment of concomitant conditions was not different between randomized groups. All analyses reported here focusing on the influence of diabetes and/or abnormal BMI were performed in the final, locked dataset assigning patients to therapy group on the basis of randomization (intention-to-treat population). Patient groups were balanced between randomized groups as can be expected in a large randomized clinical trial. The protocol was approved by the ethics review boards of all institutions involved and is available in Supplement 1. All patients participating in the trial provided written informed consent. The EAST-AFNET 4 trial followed Consolidated Standards of Reporting Trials (CONSORT) reporting guidelines.

### Statistical Analysis

All patients with the required information were categorized into binary groups on the basis of BMI (<30 vs ≥30) and the presence of diabetes (yes or no).

Patients’ baseline characteristics were summarized with descriptive statistical methods. Categorical data are shown as absolute and relative frequencies and continuous variables as mean and standard deviation.

The efficacy and safety outcomes were analyzed for an interaction between treatment group and the BMI or diabetes groups. For calculation of time-to-event outcomes, such as the first primary outcome and its components (cardiovascular death, first stroke, first hospitalization for worsening heart failure, first hospitalization for acute coronary syndrome), a Cox proportional hazards model with a frailty term for the cluster center was used. The frailty term is a statistical modeling concept, which aims to account for heterogeneity caused by unmeasured covariates due to patients treated in different countries and by different clinicians (special cluster structure in recruitment). Every study site was given a unique identifier and included as frailty in the model to increase model stability and therefore take into a cluster effect with possible multiplicative effect on the baseline hazard function. Notably, it does not take into account whether the treatment effect differs between sites. The treatment effects are expressed as cause-specific hazard ratios (HRs) and 95% confidence intervals.

The second primary outcome, nights spent in the hospital, was analyzed with a negative binomial mixed model, with total sum of nights as outcome and a treatment group and BMI/diabetes group interaction as fixed factors, site as random effect, and the log of follow-up time as the offset. The treatment effect is shown as incidence rate ratio and 95% confidence intervals.

For the key secondary outcomes (rhythm at 2 years, left ventricular ejection fraction [LVEF], quality of life, AF-related symptoms, and cognitive function), baseline-adjusted mixed linear or mixed logistic models were implemented where appropriate using a multiply imputed dataset after 60 imputations of missing values with chained equations algorithm for a set of variables based on suggestions by White, Royston, and Wood.^10^ For the key secondary outcome, only the corresponding baseline measurement, treatment group and BMI/diabetes group, and their interaction (treatment × BMI/diabetes) were included as fixed effects and site as a random effect. No medications were included. The treatment effects are presented as the adjusted mean difference or odds ratio with 95% confidence intervals.

The safety outcomes were analyzed with mixed logistic regression models with an interaction term (treatment group with BMI/diabetes groups) and site as random effect. All analyses were performed using R software version 4.1.0 (R Project for Statistical Computing).

## Results

### Efficacy and Safety of ERC Therapy in Patients With Obesity

#### Baseline Characteristics

There were 1086 patients (39.1%) with obesity (BMI ≥30,^2^ obesity classes I-III; mean [SD] BMI, 34.5 [4.2]; Table 1) and 1690 patients (60.9%) without obesity (BMI <30; mean [SD] BMI, 25.9 [2.6]). Overall mean patient age was 70 years, and 1293 patients (46.6%) were female. Patients with obesity were younger (mean [SD] age, 68 [8.6] vs 72 [7.7] years), had more frequently nonparoxysmal AF patterns (31% vs 24%), had a lower incidence of previous stroke or transient ischemic attack (9.2% vs 13.2%), and experienced arterial hypertension (94.8% vs 83.3%) and stable heart failure (32.7% vs 26.0%) more often than patients without obesity. There was no difference in mean (SD) CHA_2_DS_2_-VASc score (3.4 [1.3] vs 3.3 [1.3]) or in chronic kidney disease of Modification of Diet in Renal Disease (MDRD) stage 3 or 4 (13.5% vs 12.0%) between patients with and without obesity. Patients with obesity were more often treated with β-blockers, angiotensin-converting enzyme (ACE) inhibitors, or angiotensin II receptor blockers, diuretics, statins, and mineralocorticoid receptor antagonists at inclusion. A total of 380 of 1086 patients with BMI of 30 or higher (35.0%) had diabetes, while 312 of 1690 patients with a BMI less than 30 (18.5%) were diagnosed as having diabetes.

**Table 1.** Baseline Characteristics of Patients With Body Mass Index (BMI) <30 and With BMI ≥30 and of Patients With and Without Diabetes

Characteristic	No./total No. (%)			
	BMI		Diabetes	
	BMI <30 (n = 1690)^b^	BMI ≥30 (n = 1086)^b^	No (n = 2090)	Yes (n = 694)
Age, mean (SD), y	72 (7.7)	68 (8.6)	71 (8.2)	69 (8.6)
Sex				
Female	778/1690 (46.0)	506/1086 (46.6)	1021/2090 (48.9)	271/694 (39.0)
Male	912/1690 (54.0)	580/1086 (53.4)	1069/2090 (51.1)	423/694 (61.0)
BMI (calculated), mean (SD)^a^	25.9 (2.6)	34.5 (4.2)	28.6 (5.2)	31.3 (5.5)
AF type				
First episode	641/1688 (38.0)	400/1086 (36.8)	797/2090 (38.1)	250/694 (36.0)
Paroxysmal	641/1688 (38.0)	350/1086 (32.2)	740/2090 (35.4)	254/694 (36.6)
Persistent or longstanding persistent	406/1688 (24.1)	336/1086 (30.9)	553/2090 (26.5)	190/694 (27.4)
Sinus rhythm at baseline	942/1686 (55.9)	558/1086 (51.4)	1141/2089 (54.6)	364/693 (52.5)
Days since AF diagnosis, median (IQR)	32.0 (6.0-105.0)	42.0 (6.0-121.0)	36.0 (6.0-113.0)	35.0 (6.0-109.0)
Absence of AF symptoms	492/1598 (30.8)	301/1026 (29.3)	565/1975 (28.6)	236/658 (35.9)
Previous pharmacological or electrical cardioversion	660/1673 (39.5)	425/1069 (39.8)	820/2068 (39.7)	269/685 (39.3)
Concomitant cardiovascular conditions				
Previous stroke or transient ischemic attack	223/1690 (13.2)	100/1086 (9.2)	243/2090 (11.6)	85/694 (12.2)
At least mild cognitive impairment	713/1613 (44.2)	450/1046 (43.0)	862/2003 (43.0)	304/664 (45.8)
Arterial hypertension	1408/1690 (83.3)	1029/1086 (94.8)	1803/2090 (86.3)	644/694 (92.8)
Systolic blood pressure, mean (SD), mm Hg	136 (18.7)	139 (20.2)	137 (19.1)	138 (20.0)
Diastolic blood pressure, mean (SD), mm Hg	80 (11.8)	83 (12.2)	81 (12.2)	80 (11.3)
Stable heart failure	440/1690 (26.0)	355/1086 (32.7)	581/2090 (27.8)	215/694 (31.0)
CHA_2_DS_2_-VASc score, mean (SD)	3.4 (1.3)	3.3 (1.3)	3.1 (1.2)	4.1 (1.4)
Chronic kidney disease of MDRD stage 3 or 4	202/1690 (12.0)	147/1086 (13.5)	238/2090 (11.4)	112/694 (16.1)
Medication at discharge				
Oral anticoagulation with NOAC or VKA	1509/1686 (89.5)	999/1086 (92.0)	1879/2088 (90.0)	638/694 (91.9)
Digoxin or digitoxin	74/1686 (4.4)	57/1086 (5.2)	100/2088 (4.8)	31/694 (4.5)
β-Blockers	1326/1686 (78.6)	913/1086 (84.1)	1673/2088 (80.1)	576/694 (83.0)
ACE inhibitors or angiotensin II receptor blocker	1067/1686 (63.3)	857/1086 (78.9)	1372/2088 (65.7)	560/694 (80.7)
Mineralocorticoid receptor antagonist	87/1686 (5.2)	95/1086 (8.7)	108/2088 (5.2)	74/694 (10.7)
Diuretic	569/1686 (33.7)	548/1086 (50.5)	747/2088 (35.8)	373/694 (53.7)
Statin	683/1686 (40.5)	508/1086 (46.8)	787/2088 (37.7)	409/694 (58.9)
Platelet inhibitor	280/1686 (16.6)	170/1086 (15.7)	315/2088 (15.1)	140/694 (20.2)
Oral antidiabetics	193/1686 (11.4)	265/1086 (24.4)	4/2088 (0.2)	455/694 (65.6)
Insulin	48/1686 (2.8)	73/1086 (6.7)	0/2088	121/694 (17.4)
Planned therapy for rhythm control at baseline				
AAD	797/1690 (47.2)	468/1086 (43.1)	949/2090 (45.4)	319/694 (46.0)
Ablation	61/1690 (3.6)	52/1086 (4.8)	84/2090 (4.0)	30/694 (4.3)
None	832/1690 (49.2)	566/1086 (52.1)	1057/2090 (50.6)	345/694 (49.7)
Diabetes detailed				
No	1360/1687 (80.6)	697/1086 (64.2)	2066/2090 (98.9)	0/694
No, but impaired glucose tolerance	15/1687 (0.9)	9/1086 (0.8)	24/2090 (1.1)	0/694
Yes, currently no therapy	11/1687 (0.7)	5/1086 (0.5)	0/2090	16/694 (2.3)
Yes, insulin therapy	55/1687 (3.3)	79/1086 (7.3)	0/2090	134/694 (19.3)
Yes, managed by diet alone	64/1687 (3.8)	71/1086 (6.5)	0/2090	136/694 (19.6)
Yes, oral antidiabetics	182/1687 (10.8)	225/1086 (20.7)	0/2090	408/694 (58.8)

Abbreviations: AAD, anti-arrhythmic drug; ACE, angiotensin-converting enzyme; AF, atrial fibrillation; MDRD, Modification of Diet in Renal Disease; NOAC, novel oral anticoagulant; VKA, vitamin K antagonist.

^a^ BMI calculated as weight in kilograms divided by height in meters squared.

#### Planned Therapy for Rhythm Control at Baseline and During Follow-Up

ERC strategies stratified by BMI group are shown in eFigure 1 in Supplement 2. Of note, anti-arrhythmic drug–based therapy was the dominant ERC in both BMI groups, with flecainide, amiodarone, and dronedarone as the most frequently applied medications. While the total proportion of medication-based ERC decreased in both groups until 24-month follow-up, the use of AF ablation increased in both groups. A total of 150 of 564 patients assigned to UC and with BMI of 30 or higher (26.6%) were converted to rhythm control during follow-up, and 249 of 824 patients with BMI less than 30 (30.2%).

#### Outcome Analysis

##### Primary and Secondary Outcomes

There were numerically more outcomes in patients with obesity than in patients without obesity (Table 2). Obesity did not change the effect of ERC therapy on first primary outcome (hazard rate point estimates: BMI <30, 0.84; BMI ≥30, 0.69; *P* for interaction = .22; Table 2; Figure). When analyzing the components of the first primary outcome, there were no significant differences observed for death from cardiovascular causes (0.76 vs 0.62; *P* for interaction = .58), stroke (0.78 vs 0.34; *P* for interaction = .10), hospitalization with worsening of heart failure (0.93 vs 0.67; *P* for interaction = .12), and for hospitalization with acute coronary syndrome (0.71 vs 1.05; *P* for interaction = .35) between patients with and without diabetes. ERC did not differentially affect the second primary outcome, nights spent in hospital, in both BMI groups (BMI <30: HR, 1.13; BMI ≥30: HR, 0.97; *P* for interaction = .25). Analysis of key secondary outcomes at 2 years, including change of LVEF or functional scores (eg, EuroQol 5-Dimension or Montreal Cognitive Assessment), also did not reveal any difference between BMI groups.

**Table 2.** Primary Study Outcome for Patients With Body Mass Index (BMI) <30 and With BMI ≥30^a^

Outcome	BMI categorical				BMI continuous			
	Treatment effect (95% CI)		*P* value for interaction^b^	*P* value pooled	Treatment effect (95% CI)	BMI (95% CI)^a^	Interaction term (95% CI)	*P* value for interaction^b^
	BMI <30^a^	BMI ≥30^a^						
First primary outcome	0.84 (0.68 to 1.04)	0.69 (0.52 to 0.91)	.22	.15	1.47 (0.57 to 3.81)	1.00 (0.98 to 1.02)	0.98 (0.95 to 1.01)	.19
Components of the first primary outcome								
Death from cardiovascular causes	0.76 (0.52 to 1.11)	0.62 (0.35 to 1.09)	.58	.04	1.04 (0.16 to 6.56)	0.97 (0.93 to 1.01)	0.99 (0.93 to 1.05)	.69
Stroke	0.78 (0.50 to 1.23)	0.34 (0.13 to 0.85)	.10	.001	2.99 (0.25 to 35.62)	0.95 (0.90 to 1.00)	0.95 (0.87 to 1.04)	.23
Hospitalization with worsening of heart failure	0.93 (0.70 to 1.25)	0.67 (0.47 to 0.96)	.12	.70	2.07 (0.59 to 7.29)	1.02 (1.00 to 1.05)	0.97 (0.93 to 1.01)	.14
Hospitalization with acute coronary syndrome	0.71 (0.44 to 1.13)	1.05 (0.59 to 1.85)	.35	.90	0.64 (0.09 to 4.75)	0.99 (0.95 to 1.04)	1.01 (0.94 to 1.08)	.80
Secondary primary outcome: nights spent in hospital	1.13 (0.96 to 1.34)	0.97 (0.79 to 1.19)	.25	.35	1.614 (0.796 to 3.273)	1 (0.983 to 1.016)	0.986 (0.963 to 1.01)	.24
Key secondary outcomes at 2 y								
Change in left ventricular ejection fraction	−0.11 (−0.99 to 0.77)	0.74 (−0.29 to 1.78)	.21	.24	−3.53 (−7.17 to 0.12)	−0.11 (−0.2 to −0.03)	0.13 (0.01 to 0.25)	.04
Change in EQ-5D score	0.38 (−1.89 to 2.64)	2.42 (−0.39 to 5.24)	.27	.40	−7.35 (−17.32 to 2.61)	−0.10 (−0.33 to 0.13)	0.29 (−0.04 to 0.63)	.09
Change in SF-12 mental score	−1.33 (−2.39 to −0.28)	−0.96 (−2.28 to 0.35)	.66	.41	−2.39 (−7.08 to 2.29)	−0.06 (−0.17 to 0.04)	0.04 (−0.11 to 0.2)	.60
Change in SF-12 physical score	0.15 (−0.76 to 1.07)	0.6 (−0.49 to 1.7)	.53	.25	−2.37 (−6.12 to 1.38)	−0.13 (−0.22 to −0.04)	0.09 (−0.03 to 0.22)	.15
Change in MoCA score	−0.21 (−0.55 to 0.12)	−0.01 (−0.42 to 0.39)	.45	.95	−0.85 (−2.24 to 0.53)	−0.01 (−0.05 to 0.02)	0.02 (−0.02 to 0.07)	.30
Sinus rhythm	2.87 (2.20 to 3.73)	3.64 (2.62 to 5.05)	.267	.35	1.81 (0.57 to 5.77)	0.98 (0.95 to 1.00)	1.02 (0.98 to 1.06)	.34
Asymptomatic	1.06 (0.83 to 1.37)	1.28 (0.93 to 1.77)	.36	.71	0.56 (0.19 to 1.62)	0.99 (0.96 to 1.01)	1.02 (0.99 to 1.06)	.18
Sinus rhythm at follow-up 12	2.94 (2.24 to 3.87)	3.71 (2.65 to 5.2)	.28	.55	1.62 (0.51 to 5.1)	0.97 (0.95 to 1.00)	1.02 (0.99 to 1.06)	.23
Recurrent AF	0.82 (0.71 to 0.96)	0.75 (0.63 to 0.9)	.47	.15	1.06 (0.57 to 1.99)	1.02 (1.01 to 1.04)	0.99 (0.97 to 1.01)	.35

Abbreviations: AF, atrial fibrillation; EQ-5D, EuroQol 5-Dimension; MoCA, Montreal Cognitive Assessment; SF-12, 12-Item Short Form Health Survey.

^a^ Calculated as weight in kilograms divided by height in meters squared.

^b^ *P* value interaction calculated with likelihood ratio test.

**Figure.**
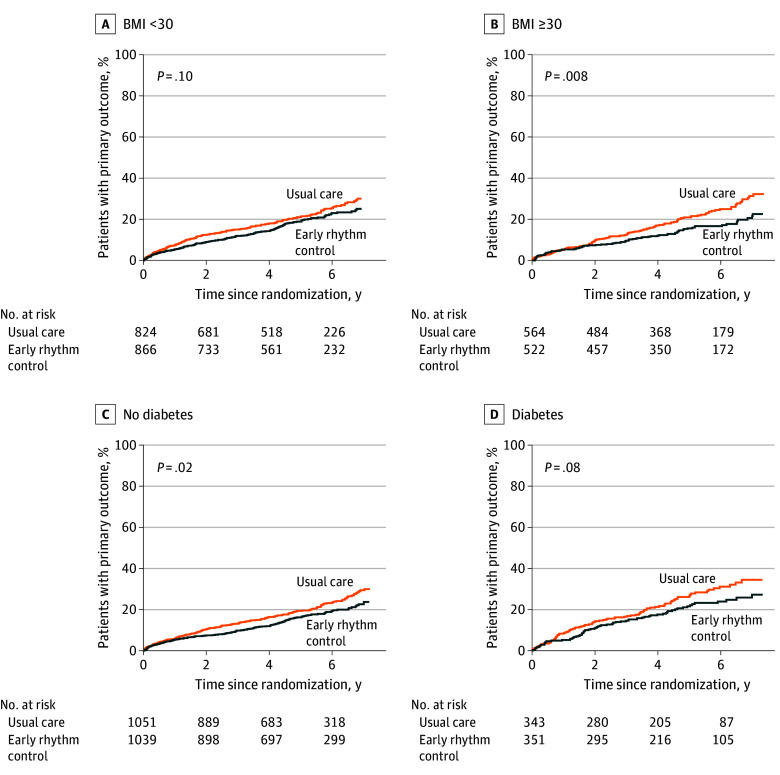
Primary Outcome for Patients With Body Mass Index (BMI) <30 (A), BMI ≥30 (B), Without Diabetes (C), and With Diabetes (D)^a^ ^a^Calculated as weight in kilograms divided by height in meters squared.

##### Safety Outcomes

There were no differences between patients with a BMI less than 30 and patients with a BMI of 30 or higher for the primary composite safety outcome (18.5% vs 13.4%; *P* for interaction = .37), as well as for stroke (3.9% vs 1.1%; *P* for interaction = .13) and for death (11.2% vs 7.9%; *P* for interaction = .43). There were also no differences when assessing serious adverse events related to anti-arrhythmic drug therapy, such as drug toxicity (0.9% vs 0.4%; *P* for interaction = .18) or drug-induced bradycardia (0.7% vs 1.5%; *P* for interaction = .99), and for serious adverse events related to AF ablation therapy, such as pericardial tamponade (0.2% vs 0.2%; *P* for interaction = .99), major bleeding (0.2% vs 0.8%; *P* for interaction = .92), and nonmajor bleeding (0% vs 0.2%; *P* for interaction > .99). All details are shown in eTable 1 in Supplement 2.

### Efficacy and Safety of ERC Therapy in Patients With Diabetes

#### Baseline Characteristics

There were 694 patients with diabetes (24.9%) in the EAST-AFNET 4 trial, with no difference between randomized groups (ERC: 351 patients [25%]; UC: 343 patients [25%]; *P* = .67). Patients with diabetes were younger (mean [SD] age, 69 [8.6] vs 71 [8.2] years; *P* = .001), more often male (61.0% vs 51.1%; *P* < .001), had a higher mean (SD) BMI (31.3 [5.5] vs 28.6 [5.2]; *P* < .001), and more often had arterial hypertension (92.8% vs 86.3%; *P* < .001), a higher CHA_2_DS_2_-VASc score (4.06 vs 3.11; *P* < .001), and chronic kidney disease of MDRD stage 3 or 4 (16.1% vs 11.4%; *P* = .003). Accordingly, patients with diagnosis of diabetes were more frequently taking guideline-conforming medication for cardiac conditions (eg, ACE inhibitor or angiotensin II receptor blocker, mineralocorticoid receptor antagonist, diuretic, statin, platelet inhibitor, or oral antidiabetics) (Table 3). Patients with diabetes were more often asymptomatic (36% vs 29%; *P* = .002). Most patients with diabetes were managed with oral antidiabetics (65.6% oral antidiabetics in patients with diabetes vs 0.2% in patients without diabetes; *P* < .001) (Table 1). A total of 380 of 694 patients with diabetes (54.8%) had a BMI of 30 or higher and 706 of 2090 patients without diabetes (33.8%).

**Table 3.** Medication at Discharge of Patients Randomized to Early Rhythm Control With and Without Diabetes

Characteristic	No./total No. (%)			*P* value
	Overall (N = 2784)	Diabetes		
		No (n = 2090 [75.1%])	Yes (n = 694 [24.9%])	
Oral anticoagulation with NOAC or VKA	2517/2782 (90.5)	1879/2088 (90.0)	638/694 (91.9)	.18
Digoxin or digitoxin	131/2782 (4.7)	100/2088 (4.8)	31/694 (4.5)	.79
β-Blockers	2249/2782 (80.8)	1673/2088 (80.1)	576/694 (83.0)	.15
ACE inhibitor or angiotensin II receptor blocker	1932/2782 (69.4)	1372/2088 (65.7)	560/694 (80.7)	<.001
Mineralocorticoid receptor antagonist	182/2782 (6.5)	108/2088 (5.2)	74/694 (10.7)	<.001
Diuretic	1120/2782 (40.3)	747/2088 (35.8)	373/694 (53.7)	<.001
Statin	1196/2782 (43.0)	787/2088 (37.7)	409/694 (58.9)	<.001
Platelet inhibitor	455/2782 (16.4)	315/2088 (15.1)	140/694 (20.2)	.002
Oral antidiabetics	459/2782 (16.5)	4/2088 (0.2%)	455/694 (65.6)	<.001

Abbreviations: ACE, angiotensin-converting enzyme; NOAC, non–vitamin K antagonist oral anticoagulant; VKA, vitamin K antagonist.

#### Planned Therapy for ERC at Baseline

There was no difference for ERC treatment strategies for patients with and without diabetes. Anti-arrhythmic drugs were started in 86% vs 87% of patients and ablation as index therapy was planned in 8.5% vs 7.9% of patients with and without diabetes, respectively. Details of ERC for patients with and without diabetes are shown in eFigure 2 in Supplement 2. Of note, the proportion of patients with ablation as the ERC strategy of choice increased significantly in both groups at 2 years after inclusion. A total of 76 of 343 patients with diabetes assigned to UC (22.2%) were converted to rhythm control during follow-up and 323 of 1051 patients with UC and no diabetes (30.7%).

#### Outcome Analysis

##### Primary and Secondary Outcomes

Diabetes did not interact with the treatment effect of ERC (patients with diabetes: HR, 0.77: without diabetes: HR, 0.78; *P* for interaction = .93) (Figure). Also, when analyzing the components of the first primary outcome, no differences were observed for death from cardiovascular causes (0.82 vs 0.67; *P* for interaction = .54), hospitalization with worsening of heart failure (0.64 vs 0.9; *P* for interaction = .17), and hospitalization with acute coronary syndrome (0.89 vs 0.8; *P* for interaction = .80). There was a numerical but non–statistically significant trend for a higher incidence of stroke in the diabetes group (0.88 vs 0.57; *P* for interaction = .33). For the secondary primary outcome, defined as nights spent in the hospital, there was no difference between both groups (1.07 vs 1.09; *P* for interaction = .91).

Key secondary outcomes, such as change in LVEF, mental and physical scores, incidence of sinus rhythm, or presence of AF-related symptoms at 2 years, were not different between patients with and without diabetes (Table 4).

**Table 4.** Primary Study Outcome for Patients With and Without Diabetes

Outcome	Treatment effect (95% CI)		*P* value for interaction	*P* value pooled
	No diabetes	Diabetes		
First primary outcome	0.78 (0.64 to 0.96)	0.77 (0.57 to 1.05)	.93	.004
Components of the first primary outcome				
Death from cardiovascular causes	0.67 (0.45 to 0.98)	0.82 (0.48 to 1.42)	.54	.04
Stroke	0.57 (0.35 to 0.93)	0.88 (0.43 to 1.8)	.33	.24
Hospitalization with worsening of heart failure	0.9 (0.69 to 1.18)	0.64 (0.43 to 0.96)	.17	.002
Hospitalization with acute coronary syndrome	0.8 (0.52 to 1.24)	0.89 (0.46 to 1.71)	.80	.12
Secondary primary outcome: nights spent in hospital	1.09 (0.94 to 1.26)	1.07 (0.83 to 1.37)	.91	.14
Key secondary outcomes at 2 y				
Change in left ventricular ejection fraction	−0.13 (−0.91 to 0.66)	1.36 (0 to 2.72)	.06	.32
Change in EQ-5D score	0.54 (−1.54 to 2.61)	2.99 (−0.56 to 6.54)	.25	.55
Change in SF-12 mental score	−1.21 (−2.2 to −0.21)	−1.07 (−2.67 to 0.53)	.89	.34
Change in SF-12 physical score	0.09 (−0.74 to 0.92)	1.17 (−0.3 to 2.63)	.21	.009
Change in MoCA score	−0.12 (−0.42 to 0.17)	−0.15 (−0.67 to 0.37)	.92	.21
Sinus rhythm	3.01 (2.38 to 3.82)	3.59 (2.4 to 5.37)	.45	.05
Asymptomatic	1.11 (0.87 to 1.4)	1.27 (0.86 to 1.87)	.55	.54
Sinus rhythm at 12 mo	2.97 (2.3 to 3.83)	4.12 (2.74 to 6.19)	.18	.08
Recurrent AF	0.83 (0.73 to 0.95)	0.69 (0.55 to 0.87)	.19	.70

Abbreviations: AF, atrial fibrillation; EQ-5D, EuroQol 5-Dimension; MoCA, Montreal Cognitive Assessment; SF-12, 12-Item Short Form Health Survey.

##### Safety Outcomes

There was no difference in safety outcomes between patients with and without diabetes (64 of 351 patients [18.2%] vs 167 of 1039 patients [16.1%], respectively; *P* for interaction = .99). Stroke and death occurred in 14 of 351 patients (4.0%) vs 26 of 1039 patients (2.5%) and in 41 of 351 patients (11.7%) vs 97 of 1039 patients (9.3%) with and without diabetes and ERC, respectively. All adverse events also related to anti-arrhythmic drug therapy and to AF ablation were not different between both groups and are listed in eTable 2 in Supplement 2.

## Discussion

This prespecified subanalysis of the EAST-AFNET 4 randomized clinical trial suggests that ERC therapy is equally effective in patients with obesity and diabetes compared to patients without obesity and diabetes. The results encourage the use of ERC therapy in patients with obesity and diabetes (ie, most patients with metabolic syndrome). Furthermore, rhythm control therapy using clinically available anti-arrhythmic drugs in most patients appeared safe and effective in patients with diabetes and patients with obesity with AF. Our analysis also confirms that obesity and diabetes are associated with a higher risk of cardiovascular events, illustrating the need for weight reduction in patients with obesity with AF^13^ and the need for good glycemic control in patients with diabetes with AF to reduce cardiovascular risk.^14^ This hypothesis-generating analysis suggests that rhythm control should be considered as well in patients with obesity and with diabetes with AF.

In view of the known cardiometabolic defects of obesity and diabetes aggravating AF,^8,15^ ventricular function,^7,15^ and AF-related outcomes,^8^ there is an understandable view that rhythm control may be futile in patients with obesity or diabetes with AF and/or that reversing obesity and treating diabetes may also reverse atrial cardiomyopathy and AF. Our analysis demonstrates that neither diabetes nor obesity affect the outcome-reducing effect of ERC. This effect is comparable to reduction in cardiovascular hospitalizations or cardiovascular death in ATHENA^16^ and the post hoc identification of lower rates of stroke with dronedarone in ATHENA.^10,17^ Considering the historic reluctance of cardiologists to offer rhythm control therapy to patients with obesity, including recommendations to achieve weight loss prior to rhythm control therapy,^5,18^ these findings are important for clinical care.

Obesity and diabetes are 2 conditions driving cardiovascular events^14^ and lead to manifestation of cardiovascular events at an early age.^19^ The younger age in patients with obesity and diabetes (Table 1) in this analysis illustrates an earlier onset of AF, in line with established risk factors for AF.^20,21,22^ AF will further add to the already heightened risk of cardiovascular events in patients with obesity and diabetes.^13^ Anticoagulation, weight reduction programs, and antidiabetic treatment can reduce risk of stroke, heart failure, and complication of diabetes and obesity in patients with AF and obesity and/or diabetes.^14^ While the pericardium contains epicardial and and pericardial adipose tissue, obesity leads to accumulation and activation of pericardial and epicardial fat, including paracrine actions of epicardial fat pads.^3,4,23^ Furthermore, increased fatty infiltration of the atria can create conduction barriers.^24^ These factors are believed to contribute to recurrent AF in patients with obesity.^25^ Weight loss, an important component in the management of patients with AF,^13^ is therefore often demanded prior to initiation of rhythm control therapy in patients with obesity with AF, based on the symptom- and rhythm-improving effects of weight loss in patients with AF.^5,26,27^ This analysis does not identify that obesity interacts with ERC, suggesting that early initiation of rhythm control therapy should be part of the initial management in patients with obesity with recently diagnosed AF.^13^ The analysis of safety outcomes suggests that established anti-arrhythmic drugs can be safely used in patients with obesity with recently diagnosed AF when suitable drugs are selected according to their safety profile and combined with electrocardiographic monitoring of QRS and QT durations during therapy initiation.^10,28^

AF bears a lifelong increased risk of death, heart failure, stroke, and dementia. Therapy of risk-enhancing cardiovascular conditions, including obesity and diabetes, but also hypertension, heart failure, and vascular disease, and initiation of anticoagulation will reduce the stroke risk and mortality.^29^ However, rhythm control was considered indicated only in patients remaining symptomatic despite rate control. Earlier secondary analysis of EAST-AFNET 4 suggested that ERC is effective in patients with and without heart failure,^30^ with and without a prior stroke,^31^ and more pronounced in patients with multiple comorbidities.^32^ The current analysis could demonstrate that the primary study end point is not affected by the diagnoses of diabetes or by a BMI less than 30 compared to patients with BMI of 30 or higher.

Patients with diabetes are at increased risk of vascular disease, including myocardial infarction and small vessel disease.^14^ These conditions, coronary microvascular dysfunction,^33^ and metabolic defects in diabetic hearts^6,34^ can increase the risk of proarrhythmic adverse effects with anti-arrhythmic drug therapy. This analysis suggests that ERC, mainly delivered using sodium channel blockers, amiodarone, and dronedarone (eFigure 2 in Supplement 2), effectively reduces outcomes and has a good long-term safety profile in patients with diabetes with recently diagnosed AF. When this analysis was planned, a high risk of recurrent AF was expected in patients with obesity with AF.^3,5,18,35,36^ Similarly, a higher risk of recurrent AF and a higher risk of rhythm control–associated adverse events was expected in patients with diabetes. This knowledge, based on mechanistic, translational, and observational clinical data,^3,4,23,24,25^ led to expectation of futility of ERC. To the contrary, this analysis showed that ERC is similarly effective and safe in patients with diabetes and in patients with obesity compared to patients without diabetes and without obesity with AF. Put simply, neither obesity nor diabetes should be a reason to withhold ERC therapy in patients with AF.

### Strengths and Limitations

Strengths of the analysis include a prespecified subanalysis and large groups of patients with and without obesity and with and without diabetes, adjudicated outcome collection, and long-term follow-up for a median of 5.1 years. Several limitations must be noted. First, the nature of the current analysis is exploratory. Second, while all patients with AF received therapy of their multiple concomitant conditions (Table 1), the EAST-AFNET 4 trial did not include specific interventions for weight reduction in its protocol. Furthermore, the trial was conducted before glucagon-like peptide-1 receptor agonists (GLP-1 RAs) became available. While it is likely that such interventions will reduce weight and cardiovascular outcomes, the lack of interaction of ERC across the ranges of BMI suggests that ERC will retain its efficacy in patients with obesity and diabetes receiving such interventions. Third, the outcome-reducing effect of ERC therapy found in this analysis should be tested in weight-controlled cohorts, especially in patients treated with GLP-1 RAs.^37,38^ Fourth, management of diabetes used the therapies available during the conduct of the trial. Whether the effect of rhythm control remains present in patients with diabetes treated with modern antidiabetic drugs (eg, sodium-glucose cotransporter 2 [SGLT2] inhibitors) that improve cardiac metabolism^7^ and reduce the risk of AF^39,40^ remains to be tested. Fifth, the treatment effects observed here are limited to rhythm control initiated in the first year after diagnosing AF. Whether they apply to patients with obesity or diabetes with longer AF durations (ie, legacy AF patients) needs to be tested. Sixth, while the effect of ERC therapy was consistent across the spectrum of BMI, there were relatively few patients with morbid obesity enrolled in the study. Further validation in larger datasets is warranted. Seventh, the study does not account for changes in BMI over the follow-up period. Eighth, conceptually, early AF ablation may further reduce outcomes compared to the mainly drug-based therapy evaluated here (EASThigh-AFNET 11 [NCT06324188]). Ongoing trials will determine the role of early AF ablation for outcome reduction. Based on several smaller randomized trials,^41,42^ patients with AF and reduced left ventricular function may be considered for AF ablation rather than drug-based ERC.^13^ Ninth, obesity and diabetes are associated with heart failure with preserved ejection fraction (HFpEF).^30^ Ongoing and planned trials will determine whether early AF ablation improves outcomes in patients with HFpEF and AF (CABA-HFPEF [NCT05508256]).

## Conclusions

This secondary analysis of the EAST-AFNET 4 randomized clinical trial shows that early rhythm control therapy retains its effectiveness and safety in patients with and without diabetes and patients with and without obesity, including some patients with severe obesity.

## Notes

Supplement 1. Trial Protocol

Supplement 2. eTable 1. Safety Outcomes for BMI Categories eTable 2. Safety Outcomes for ERC and UC for Patients With and Without Diabetes eFigure 1. Rhythm Control Chosen by Treatment Group for Patients With a BMI <30 kg/m2 and With a BMI ≥30 kg/m2 eFigure 2. Rhythm Control Chosen by Treatment Group for Diabetes and for Non-Diabetes Patients eFigure 3. CONSORT Checklist eAppendix. Links to Study Protocol and Statistical Analysis Plan

Supplement 3. Data Sharing Statement
