# Supplementary material for: Diabetes and Obesity and Treatment Effect of Early Rhythm Control vs Usual Care in Patients With Atrial Fibrillation: A Secondary Analysis of the EAST-AFNET 4 Randomized Clinical Trial
Source: JAMA Cardiol. 2025 Jul 30;10(9):932–41. doi: 10.1001/jamacardio.2025.2374 (PMC12311819; doi:10.1001/jamacardio.2025.2374)
Supplement: Supplement 1. — Trial Protocol [file jamacardiol-e252374-s001.pdf]

CLINICAL TRIAL PROTOCOL

EAST: <u>E</u>arly treatment of <u>A</u>trial fibrillation for <u>S</u>troke prevention <u>T</u>rial

An **I**nvestigator-driven, **P**rospective, Parallel-group, **R**andomized, **O**pen,
Blinded Outcom**e** Assessment (PROBE-design), Multi-centre Trial
for the Prevention of Stroke in High-risk Subjects with Atrial Fibrillation.

EudraCT number: 2010-021258-20
ISRCTN04708680
ClinicalTrial.gov: NCT01288352

<u>Responsible Sponsor:</u>
EAST is conducted jointly by the German Atrial Fibrillation NETwork (AFNET)
and the European Heart Rhythm Association (EHRA),
a branch of the European Society of Cardiology (ESC).
The legal sponsor of EAST is the German Atrial Fibrillation NETwork (Kompetenznetz Vorhofflimmern e.V. [AFNET])

<u>Version control:</u>
Final, dated September 21st, 2010
Amended, dated December 10th, 2010
Amended, dated April 11th, 2011
Amended, dated October 11th, 2011
Amended, dated December 9th, 2011
Amended, dated May 10th, 2013
Amended, dated May 14th, 2015
Amended, dated August 12th, 2019

Prepared by:
Elisabeth Freund, MSc, CRI and Sabine Jürgensmeyer, PhD, AFNET

COORDINATING INVESTIGATORS

Prof. Paulus Kirchhof, Münster, Germany and Birmingham, UK (International Chief Investigator)
Prof. Günter Breithardt, Münster, Germany
Prof. Harry Crijns, Maastricht, The Netherlands
Prof. John Camm, London, UK

Coordinating Address:

Kompetenznetz Vorhofflimmern e.V. (AFNET e.V.)
[Atrial Fibrillation Network]
Mendelstraße 11
D-48149 Münster, Germany
Phone: +49-251-980 1340
Fax: +49-251-980 1349
E-mail: info@kompetenznetz-vorhofflimmern.de
http://www.kompetenznetz-vorhofflimmern.de/english

CONTRACT RESEARCH ORGANISATION (CRO)

CRI – The Clinical Research Institute GmbH
Arnulfstr. 19,
D-80335 Munich, Germany
Phone: +49-89-990 1649-990 (Study Hotline)
Fax: +49-89-990 1649-890
E-mail: east@cri-muc.eu

All information in this study protocol including attachments provided to you as investigator, potential investigator, co-investigator or committee member must be treated confidentially. The right to use this information is limited to you, your staff, members of the relevant committees or entitled authorities. The objectives and the content of this study, as well as its results, must be treated confidentially and may not be made available to third parties at any time before, during and after the study without written approval of the sponsor except to the extent necessary to get informed consent from patients. This applies to investigators and all supporting staff involved in the study. Transmission, duplication or use for publication is permitted only with the written agreement of the sponsor.

This protocol has been written in accordance with current applicable guidelines (ICH-GCP and EU-Directive 2001/20/EC) as well as all other relevant additional references, medical and legal ones.

Table of Content

1 Abbreviations....8
2 Trial summary....10
3 Introduction....12
3.1 Background Information....12
3.2 Study Rationale....12
3.3 Benefit-risk assessment....13
4 Study Objectives....14
4.1 Primary outcome parameter....14
4.2 Secondary outcome parameters....14
4.3 Safety outcome parameters....15
5 Study Design....15
5.1 Study Flow Chart....16
5.2 Selection of study sites....16
6 Selection of Patients....17
6.1 Informed Consent....17
6.2 Study Population....17
6.2.1 Number of Patients....17
6.2.2 Inclusion criteria....17
6.2.3 Exclusion criteria....18
6.2.4 Randomisation....19
7 Therapy....19
7.1 Standard management of AF....19
7.2 Recommendations for usual care....20
7.3 Recommendations for early therapy of AF....20
7.3.1 Antiarrhythmic rhythm control drug therapy....20
7.3.2 Rate control therapy....23
7.4 Concomitant medication....24
7.5 Cardioversion....24
7.6 Catheter ablation....24
7.6.1 Re-ablation with the aim to re-isolate the pulmonary veins....25
7.6.2 Other ablation targets....25
7.7 Post-study treatment....25
8 Assessing and Reporting of Adverse Events....26
8.1 Adverse Events of Special Interest....26
8.2 Serious Adverse Events....26

8.3 Recording and Reporting Serious Adverse Events and Adverse Events of Special Interest (if serious) ... 27
8.3.1 Definition of Intensity ... 27
8.3.2 Definition of Causality ... 28
8.3.3 Adverse Event Follow-up Procedures ... 28
8.3.4 Suspected Unexpected Serious Adverse Events (SUSARs) ... 28
8.3.5 Pregnancy ... 28
9 Study Schedule ... 28
9.1 Visit schedule ... 28
9.2 Baseline Visit ... 30
9.3 Follow-up ... 30
9.3.1 Clinical Visits (Months 12, 24) ... 30
9.3.2 Central Follow-up ... 31
9.3.3 Triggered Visits ... 31
9.4 Contact at (premature) study termination ... 31
9.5 Transthoracic Echocardiography ... 31
9.6 Electrocardiogram ... 32
9.7 Blood Samples ... 32
9.8 ECG monitoring ... 32
9.9 Sub-Studies ... 33
10 Duration of study participation ... 33
10.1 Overall Duration of Study ... 33
10.2 Individual duration of Study ... 33
11 Stopping and Discontinuation Criteria ... 33
11.1 Discontinuation criteria related to the study ... 33
11.2 Discontinuation criteria related to the patient ... 33
12 Statistics ... 34
12.1 Statistical Methods ... 34
12.1.1 Analysis of the primary outcome ... 34
12.1.2 Analysis of secondary outcomes ... 34
12.1.3 Subgroup analyses ... 35
12.1.4 Safety analyses ... 35
12.2 Sample Size Calculation ... 35
12.3 Patient Selection for Analyses ... 35
13 Access to Source Data / Documents ... 36
13.1 Source Data ... 36
13.2 Source Documents ... 36
13.3 Direct Access ... 36

14 Quality Control and Quality Assurance 36
14.1 Quality Control 36
14.2 Initiation Visit 37
14.3 Study Monitoring 37
14.4 Close Out Visit 37
14.5 Quality Assurance 37
14.5.1 Inspections 37
14.5.2 Audits 38
15 Ethical and Legal Consideration 38
15.1 Ethical consideration 38
15.1.1 Institutional Review Board/ Independent Ethics Committee (IRB / IEC) 38
15.1.2 Steering Committee 38
15.1.3 Executive Steering Committee 39
15.1.4 Endpoint Review Committee 39
15.1.5 Data and Safety Monitoring Board 39
15.2 Legal Consideration 39
15.3 Modification of Protocol 40
15.4 Financing and Insurance 40
15.5 Investigator's Information on Study Treatment 40
15.6 Personal Data and Data Protection 40
15.7 Data Handling and Record Keeping 40
15.7.1 Completion of Case Report Forms 40
15.7.2 Archiving 41
15.8 Confidentiality 41
15.9 Responsibilities 41
16 Final Report and Publication Policy, Property Rights 41
17 Definitions and Classifications 42
17.1 Protocol Violation 42
17.2 Major Protocol Violation 42
17.3 Protocol Deviation 42
18 References 43
19 Signatures 48
20 Appendices 49
Appendix I: List of participating countries / national principal investigators 49
Appendix II: List of participating study sites 50
Appendix III: Members of the Steering Committee 51
Appendix IV: Members of the Executive Steering Committee 53
Appendix V: Members of the Endpoint Review Committee 54

Appendix VI: Members of the Data and Safety Monitoring Board 55
Appendix VII: (deleted with protocol amendment 14.05.2015) 56
Appendix VIII: Patient Information and Informed Consent 57
Appendix IX: Time Schedule 65
Appendix X: Definitions of study assessments 66
Appendix XI: Design and sample size calculation 71
Appendix XII: MoCA test 73
Appendix XIII: EQ-5D questionnaire 74
Appendix XIV: SF-12 76
Appendix XV: Description of ECG monitoring 77
Appendix XVI: Declaration of Helsinki (Version Seoul, October 2008) 78
Appendix XVII: Consensus statement of HRS/EHRA for catheter ablation of atrial fibrillation (Calkins et al Europace 2007). 79
Appendix XVIII: ESC 2012 guidelines for the management of patients with atrial fibrillation 80
Appendix XIX: Karnofsky score 81

1 Abbreviations

**ACC**	American College of Cardiology
**ACS**	acute coronary syndrome
**AE**	adverse event
**AF**	atrial fibrillation
**AF-CHF**	Atrial Fibrillation Congestive Heart Failure trial
**AFNET**	Atrial Fibrillation Network
**ALAT**	Alanin Aminotransferase
**BID**	twice-daily
**CABG**	coronary artery bypass graft
**CIOMS**	Council for International Organizations of Medical Sciences
**CRF**	case report form
**CV**	curriculum vitae
**CRI**	The Clinical Research Institute GmbH
**CRO**	Contract Research Organisation
**CRP**	C-reactive protein
**CYP450 3A4**	Cytochrome P450 3A4- the most abundant of the P450 enzymes, responsible for initial drug metabolism in the liver
**DSMB**	Data and Safety Monitoring Board
**EAST**	Early treatment of Atrial fibrillation Stroke prevention Trial
**ECG**	electrocardiography
**e-CRF**	electronic case report form
**EHRA**	European Heart Rhythm Association
**EQ-5D**	Euroquol 5D questionnaire
**ERC**	Endpoint Review Committee
**ESC**	European Society of Cardiology
**eSC**	Executive Steering Committee
**FU**	follow-up
**GCP**	Good Clinical Practice
**GFR**	glomerular filtration rate
**HF**	heart failure
**HRS**	Heart Rhythm Society
**ICH**	International Conference on Harmonisation
**ICF**	informed consent form
**IEC**	Independent Ethics Committee

**INR**	International Normalized Ratio
**IIT**	investigator initiated trial
**IRB**	Institutional Review Board
**LA**	left atrium
**LBBB**	left bundle branch block
**LV**	left ventricle
**LVEF**	left ventricular ejection fraction
**MARVIN**	Electronic trial management system used for the EAST study; provides the e-CRF as well as business logic and management tools for data documentation, management and cleaning
**MDRD**	Modification of Diet in Renal Disease
**MI**	myocardial infarction
**MoCA**	Montreal cognitive assessment
**MRI**	magnetic resonance imaging
**NSTEMI**	non-ST-segment elevation myocardial infarction
**NYHA**	New York Heart Association
**PCI**	percutaneous coronary intervention
**PE**	physical examination
**PI**	principal investigator
**PVI**	pulmonary vein isolation
**QoL**	Quality of life
**SAE**	serious adverse event
**SF-12**	12-item Short-Form health survey
**STEMI**	ST-segment elevation myocardial infarction
**TIA**	transient ischemic attack
**TTE**	transthoracic echocardiography

2 Trial summary

TITLE	**E**arly treatment of **A**trial fibrillation for **S**troke prevention **T**rial (**EAST**)
INVESTIGATOR/ TRIAL LOCATION	11 European countries
STUDY OBJECTIVE(S)	To test whether an early, comprehensive, rhythm control therapy can prevent adverse cardiovascular outcomes in patients with atrial fibrillation (AF) compared to usual care
STUDY DESIGN	Investigator-driven, Prospective, parallel-group, randomized, open, blinded outcome assessment (PROBE) parallel-group interventional multi-centre trial.
STUDY POPULATION<br>Main selection criteria	The following criteria must be present for eligibility into the study:<br>▪ Recent onset AF, i.e. AF with a known history of ≤ 1 year prior to randomisation<br>▪ Risk for stroke as evidenced by **EITHER**<br>a) <u>one of the following</u>: age > 75 years, prior stroke or transient ischemic attack (TIA) **OR**<br>b) <u>two of the following</u>: hypertension, diabetes mellitus, left ventricular hypertrophy, age > 65 years, female sex, peripheral artery disease, kidney disease (MDRD stage III or IV), stable heart failure (NYHA II or LVEF <50%), severe coronary artery disease (previous myocardial infarction, CABG or PCI)
Total expected number of patients	2,745 patients to be randomized
Expected number of sites	Approximately 200 study sites including 40-50 ablation sites
INVESTIGATIONAL INTERVENTIONS<br><br>Brief description of used pharmacological products, formulations, route of administration dose regimen and interventional procedures	EAST prospectively tests the hypothesis that an early, structured rhythm control therapy based on antiarrhythmic drugs and catheter ablation can prevent AF-related complications in patients with AF when compared to usual care. Patients will be randomized to early therapy or usual care. In the **early therapy group**, patients will receive either catheter ablation (usually by pulmonary vein isolation), or adequate antiarrhythmic drug therapy at an early time point. The initial therapy will be selected by the local investigator. Upon AF recurrence, both modalities will be combined. **Usual care** will be conducted following the current ESC guidelines for AF treatment. Early rhythm control therapy will be guided by ECG monitoring.
PRIMARY OUTCOME PARAMETER	A composite of cardiovascular death, stroke / transient ischemic attack (TIA), and hospitalization due to worsening of heart failure or due to acute coronary syndrome.<br>The 1st co-primary outcome parameter is defined as the time to the first occurrence of a composite of the a.m. components. The 2nd co-primary outcome is nights spent in hospital per year.

SECONDARY OUTCOME PARAMETER	Several secondary outcomes will be assessed in the study population. Key secondary outcomes: Each of the components of the primary outcome, time to recurrent AF, cardiovascular hospitalizations, all-cause hospitalizations, left ventricular function, quality of life, cognitive function, cost of therapy. These and additional secondary outcome parameters will be assessed in the main trial and in investigator driven sub-studies.<br>Assessment of safety: The primary safety outcome comprises all deaths, the components of the primary efficacy parameter plus other adverse events related to the study intervention with special emphasis on proarrhythmia and complications due to interventions.
ASSESSMENT SCHEDULE	6-monthly central follow-up contacts (questionnaires) to assess (outcome) events. At months 12 and 24 clinical outpatient follow-up visits.
STATISTICAL CONSIDERATIONS	Efficacy:<br>Description of the primary efficacy analysis and population: The primary analysis is in the intention-to-treat population, consisting of all randomized patients with at least one follow-up assessment. A two-group comparison of the time to the 1st co-primary outcome will be performed using a logrank test adjusted to the group sequential design in a way that a two-sided overall significance level of 5% is kept, of which 4% are spent on the 1st co-primary, and 1% on the 2nd co-primary outcome.<br>Safety: All primary outcomes including all events that result in a hospitalization will be centrally adjudicated by an independent blinded Endpoint Review Committee. Another independent Data and Safety Monitoring Board will monitor the trial. Adverse event frequencies will be compared between groups according to type of event and organ. All interventions planned in EAST are in-line therapies. EAST tests a novel therapeutic strategy, early rhythm control therapy, that applies established therapies within approved indications at an early point in time.
DURATION OF STUDY PERIOD<br>(per patient)	First-patient-in to last-patient-out: approx. 8 years<br>EAST is an event-driven trial, i.e. the trial will be terminated after 685 evaluable primary outcomes have occurred. A duration of the entire trial of around 8 years is expected. All patients will be followed-up until the end of the trial with a minimum follow-up period of 30 months.

3 Introduction

3.1 Background Information

Atrial fibrillation (AF) affects 1-2% of the population in Europe [1-3]. It is estimated that one in four forty-year-old adults will develop AF during their lifetime [4]. Moderately effective methods to terminate and prevent AF by ion-channel blocking drugs are available [5-7]. These have recently been supplemented by catheter-based ablation of the pulmonary veins [8, 9]. Despite the clear association between AF and premature death [3], ischemic stroke [10, 11], and cardiovascular events, there is no systematic evidence that rhythm control treatment improves outcome in AF patients [12-17]. These findings notwithstanding, subgroup analyses, e.g. of the AFFIRM trial [18], and the outcome of the recently published ATHENA trial [19] support the epidemiological observation that presence of AF is one of the few modifiable factors associated with death in AF patients. Apart from the effect of dronedarone on a composite outcome driven by cardiovascular hospitalizations in the ATHENA trial [19], there are no controlled data that show a benefit of rhythm control therapy beyond improved quality of life: The major studies were the Atrial Fibrillation Follow-up Investigation of Rhythm Management (AFFIRM) trial [12], the Rate Control versus Electrical Cardioversion (RACE) trial [13], and most recently, the Atrial Fibrillation Congestive Heart Failure (AF CHF) trial [17]. There was also a series of smaller studies performed, including the Pharmacological Intervention in Atrial Fibrillation (PIAF) [16], Strategies of Treatment of Atrial Fibrillation (STAF) [14], and How to Treat Chronic Atrial Fibrillation (HOT CAFÉ)[15], among others. Virtually all studies have shown that primary rate control is not inferior to rhythm control. Meta-analysis has demonstrated no significant excess or reduced mortality with either strategy [20]. Interestingly, the PIAF trial which enrolled patients with a short history of AF (< 1 year) found an improvement of exercise capacity in the rhythm control arm when compared to a rate control therapy, despite a relatively small patient number [16].

Hence, current guidelines for the treatment of AF and medical practice base the decision for “rhythm control” **or** “rate control” treatment on individual factors that are often influenced by non-systematic impressions of the treating physician [2, 6, 21]. Furthermore, therapy for AF is only indicated in patients with recurrent AF [2, 6, 21]. In summary, the treating physician is left alone in the important therapeutic decision as to whether pursuing sinus rhythm is important in a given patient. This lack of evidence is unacceptable.

3.2 Study Rationale

Why has rhythm control therapy of AF not been effective in the prevention of deaths and stroke? This may in part be due to the fact that the tested interventions, mainly ion channel-blocking drugs, may have proarrhythmic effects, especially in patients with left ventricular hypertrophy or severe heart failure [22]. Proarrhythmia, however, is a relatively rare event, even in high-risk patient groups. More importantly, the rhythm control interventions used in the published trials were only moderately effective (e.g. sinus rhythm rates at the end of follow-up in the AFFIRM trial were 30% in the “rate control” group and 60% in the rhythm control group [12]). It has been suspected that the negative outcome of rhythm control therapy in the AFFIRM trial is a consequence of “positive patient selection”; i.e. that enrolled patients were selected by having survived a phase of AF-related complications. Furthermore, the AF-CHF trial probably suffered from a similar bias by preferably enrolling patients without marked AF-related symptoms. Much of the atrial damage induced by AF was probably irreversible in the AF-CHF patients (e.g. 3.5% stroke, or 1% per year). Interestingly, stroke rates in AF-CHF were numerically smaller in the rhythm control group (3% vs. 4% [17]), as well as in the dronedarone arm of the ATHENA trial [36]. Furthermore, anticoagulant therapy was often withdrawn from patients in rhythm control arms, e.g. in the AFFIRM trial, based on the assumption that sinus rhythm was present,

resulting in a potentially avoidable excess risk of ischemic stroke, potentially induced by asymptomatic recurrences of AF [18].

The causes underlying AF are multifactorial. AF itself causes marked changes in atrial electrophysiology (“electrical remodelling”) and in the molecular function and structure of the atria ("structural remodelling"). Ion channel-blocking antiarrhythmic drugs, the main intervention in the published “rhythm control” trials, may counter the “electrical remodelling”, but leave other mechanisms untouched. These vicious circles initiate and maintain AF, and contribute to AF-related complications [23, 24].

The marked molecular and structural changes in the atria (“atrial cardiomyopathy”) induced by AF include calcium overload, atrial fibrosis, myolysis, myocellular hypertrophy, activation of the rennin-angiotensin system, and atrial contractile dysfunction [23]. These profound changes may still be reversible during early phases of the arrhythmia, but provoke relevant and permanent atrial damage during later stages of AF [25].

Furthermore, many patients with AF suffer from focal triggering sources that initiate AF [9]. These focal initiators can be treated by isolation of the pulmonary veins (“catheter ablation of AF” [8]) [37].

Taken together, insufficient, non-structured and delayed therapy of the multiple factors that lead to AF, maintain it, and cause its complications have most likely contributed to the limited efficacy of rhythm control interventions in the past “rhythm control strategy” trials. There is, hence, a growing need for a trial that tests whether a structured and early antiarrhythmic intervention is beneficial for AF patients [26]. The tools for such an early AF therapy are available to clinical cardiologists, but have so far not been systematically applied to the AF patient population.

This trial protocol, therefore, suggests a controlled trial that tests the hypothesis that an early, standardized rhythm control therapy strategy can prevent cardiovascular outcomes attributable to AF. This therapeutic strategy will be compared to usual care as defined by the ESC guidelines for the treatment of AF.

3.3 Benefit-risk assessment

Early, structured rhythm control therapy has the potential to improve prognosis of AF patients by preventing AF-related cardiovascular complications.

If the EAST trial would confirm the hypothesis that early rhythm control therapy of AF helps to reduce cardiovascular complications in AF, this would benefit the trial patients and a large portion of the current population of patients in AF. The risk of the participating patients appears small given the fact that all therapies are approved and applied in-line with current recommendations and guidelines. It is likely that the trial patients will benefit from the structured, well-controlled application of guideline-conform patient management in both trial groups.

In addition to the therapeutic benefit this trial will further enhance the pathophysiological understanding of AF.

Telemetric ECG screening is used as a consistent monitoring tool in the structured rhythm control group and may help to prevent future strokes in a patient population at potential risk for stroke [32, 33], reflecting the spreading notion that asymptomatic AF is a common first manifestation of stroke [11]. Patients in the usual-care group may receive better than average attention of cardiovascular disease or risk factors due to the structured follow-up in a cardiac centre in the setting of a controlled trial (study site).

As all treatments in EAST are in-line with clinical practice and recommended by guidelines, adverse events are expected to occur in similar clinical manifestations and at a comparable rate as the known adverse events of the approved therapies applied in the trial.

In light of the safety profile of the employed, established drugs and the frequency of AF and its possible cardiovascular complications the benefit-risk assessment turns out to be positive.

4 Study Objectives

4.1 Primary outcome parameter

To test whether an early, comprehensive, standardized rhythm control therapy can prevent clinically relevant outcome events in patients with AF in comparison to current best clinical routine. The two components of the co-primary outcome parameter assess clinically relevant outcomes from the perspective of the patient (cardiovascular death, stroke, acute heart failure, acute coronary syndromes) and from the perspective of the health care system (nights spent in hospital).

The 1st co-primary outcome parameter is defined as the time to the first occurrence of a composite of

- cardiovascular death,
- stroke or transient ischemic attack with matching lesion on imaging (ischemic stroke and hemorrhagic stroke, includes intracranial haemorrhage),
- worsening of heart failure, and
- acute coronary syndrome,

the latter two assessed by hospitalizations.

The 2nd co-primary outcome parameter is nights spent in hospital per year. This parameter integrates a majority of health care expenditures and medical efforts in the management of the EAST trial population. Nights spent in hospital was chosen over other parameters because it is easily and objectively counted.

4.2 Secondary outcome parameters

The secondary outcome parameters are defined as

- all-cause death,
- AF-related death,
- time to the first occurrence of each of the components of the 1st co-primary outcome,
- time to recurrent AF (paroxysmal, persistent, long-lasting persistent, permanent),
- AF burden,
- time to first therapy change,
- time to first cardiovascular hospitalization,
- number of cardiovascular hospitalizations (over-night stay),
- left ventricular function at 24 months,
- quality of life changes at 24 months and at study termination compared to baseline,
- health-related cost calculation estimated by quantification of interventions, nights spent in hospital, and cost of outpatient treatment,
- change of cognitive function (MoCA) at 24 months compared to baseline,
- cardiac rhythm (sinus rhythm vs. AF),
- time to first symptomatic AF recurrence,
- time to first progression of AF (from paroxysmal to persistent or long-lasting persistent or permanent and each of these components).

Some further outcome parameters will be investigated in sub-studies that will apply additional tests such as intensified ECG monitoring, advanced imaging techniques such as three-dimensional echocardiography, or cerebral magnetic resonance imaging, among others.

4.3 Safety outcome parameters

The **primary safety outcome parameter** is a composite of death including cardiovascular death, stroke/TIA, and serious adverse events of special interest (proarrhythmia and other rhythm control therapy-related adverse events; refer to section 8.1). **Secondary safety outcome parameters** are the components of this composite, the number of serious adverse events of all types and of each type separately.

All outcome events will be centrally adjudicated by the Endpoint Review Committee (ERC; refer to section 15.1.4). All safety outcome parameters will be analysed descriptively only but are not part of the biometrical model of the trial.

5 Study Design

EAST is an investigator-driven, prospective, parallel-group, randomized, open, blinded outcome assessment (PROBE) controlled multi-centre study. The trial tests whether an early, comprehensive, standardized rhythm control therapy based on catheter ablation and antiarrhythmic drugs in addition to standardized therapy of underlying and concomitant cardiac diseases and conditions and antithrombotic therapy can prevent cardiovascular outcomes attributable to AF compared to usual care as defined by the current European guidelines for the management of AF. The trial will be conducted in several European countries (Appendix I).

Patient recruitment is expected to be completed after 65 months.

EAST is an event-driven trial with a planned number of randomised patients of n=2,745 and a fixed number of events (n=685). The total duration of the trial is an estimate based on observed outcome rates in other large trials with similar populations. The total number of events in the trial is depending on the time at risk, that is the follow-up time of all patients. In practice, the event-driven design may result in slight variation of the expected trial duration and of the total number of patients enrolled if observed event rates do not exactly match the projected rates. All patients will be followed until the end of the trial.

Interim analyses:

The progress of the trial will be monitored regularly in a blinded manner by the members of the Executive Steering Committee. Special attention will be given to the recruitment process (blinded) and the therapies applied in both arms of the study (unblinded). If required, specific recommendations will be given to keep the trial on track. A blinded re-assessment of the sample size will be performed by the trial statistician (according to rules laid down in the statistics section of this protocol) after 42 months or when 2,400 patients have been included, whatever comes first. Given the estimated sample sizes for the two co-primary outcomes, this re-assessment will be based on the first co-primary outcome. Based on these calculations, the executive Steering Committee decides on the further recruitment plan of the study.

A group sequential design according to O'Brien-Fleming with three interim analyses will be used to allow an early termination of the trial if results are clear. Details are to be found in the statistics section (section 12).

Documentation of all study relevant data will be performed in an electronic trial management system called MARVIN. MARVIN provides the Electronic Case Report Forms (e-CRFs) as well as business logic and management tools for data documentation, management and cleaning. Details regarding e-CRF-completion and how to use MARVIN are described in a separate data entry user manual.

5.1 Study Flow Chart

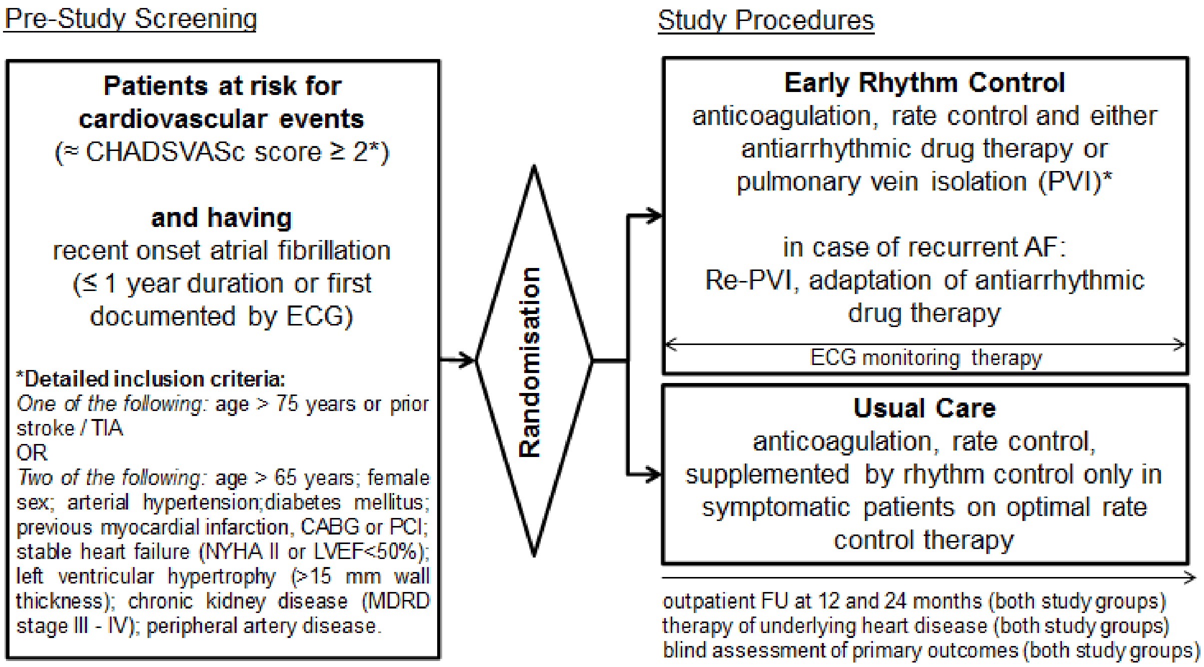

* The initial therapeutic decision for interventional or drug therapy will be left with the treating physician in the study site. Given the fact that early AF therapy will be applied irrespective of AF-related symptoms, therapeutic safety is expected to be the decisive factor for the initial choice of therapy.

5.2 Selection of study sites

Based on recommendations and surveys, the expected therapeutic strategy in the usual care group will usually be rate control, and the initial therapeutic decision will often be antiarrhythmic drug therapy in the early therapy group. The structure of study sites is supposed to reflect this ratio to guarantee a treatment close to clinical routine and medical guidelines. Therefore, local study networks will be created consisting of one ablation site (A-site) and 3-6 drug management sites (D-sites) without ability to perform ablation procedures. Each A-site plus its surrounding D-sites create one study cluster.

All study sites will be contracted and are able to screen, randomise, and follow study patients. The A-sites will perform all study-related ablation procedures in this study cluster. In addition, study sites are encouraged to recruit patients in “screening facilities” with access to patients at high risk for AF, e.g. stroke units or neurological departments, specialised diabetes or hypertension outpatient clinics etc.

6 Selection of Patients

6.1 Informed Consent

A signed, ethics committee/IRB approved informed consent form (Appendix VIII), written in accordance with country-specific applicable data privacy acts, the Declaration of Helsinki (Appendix XVI) and the applicable laws for research using medical devices and drugs, will be obtained from every patient prior to any study-related procedure. Screening assessment such as blood sampling or recording of a resting ECG is considered to be performed routinely during clinical routine and therefore is not considered to be part of study related procedures.

The investigator or responsible medical staff (or other designated research staff if permitted by the relevant national regulations) will explain the nature, purpose and risks of the study and provide the patient with a copy of the patient information sheet (Appendix VIII). The patient will be given sufficient time to consider the study's implications before deciding whether to participate.

Should there be any amendments to the protocol, such that would directly affect the patients' participation in the study, e.g. a change in any procedure, the informed consent form must be amended to incorporate this modification and the patients must agree to sign this amended form indicating that they re-consent to further participate in the modified study.

A signed copy of the patient’s informed consent form must be maintained in the study files. The patient’s permanent medical records should indicate the patient's study participation. A patient information sheet will be handed out to the patient unless declined by him/her.

6.2 Study Population

The intended population for this study is patients who present with documented AF whereas the AF episode must last longer than 30 s (at least one ECG documentation by any type of ECG) fulfilling the inclusion criteria as listed below. Patients will be recruited from contracted study sites only. Screening of potentially eligible patients will also be performed in facilities where a population with a high risk for AF is expected, e.g. in neurology clinics and stroke units, in hypertension clinics etc. (so called “screening facilities”), i.e. from institution’s broad referral network.

6.2.1 Number of Patients

A total of 2,745 patients will be randomised. The sample size anticipates a loss-to-follow-up of 5% of the total observation time. The sample size may be adapted once in a blinded manner as described in the statistics section (section 12).

Patients will be recruited in approximately 200 study sites including 40-50 ablation sites throughout Europe.

6.2.2 Inclusion criteria

I1. Recent-onset AF (≤ 1 year prior to enrolment)

I2. At least one ECG within recent 12 months that documents AF whereas the AF episode must last longer than 30 s.

I3. One of the following

- age > 75 years or
- prior stroke or transient ischemic attack

OR

two of the following

- age > 65 years,
- female sex,
- arterial hypertension (chronic treatment for hypertension, estimated need for continuous antihypertensive therapy or resting blood pressure > 145/90 mmHg),
- diabetes mellitus
- severe coronary artery disease (previous myocardial infarction, CABG or PCI)
- stable heart failure (NYHA II or LVEF <50%),
- left ventricular hypertrophy on echocardiography (more than 15 mm wall thickness),
- chronic kidney disease (MDRD stage III or IV),
- peripheral artery disease.

I4. Provision of signed informed consent.

I5. Age ≥ 18 years.

6.2.3 Exclusion criteria

General exclusion criteria

E1. Any disease that limits life expectancy to less than 1 year.

E2. Participation in another clinical trial, either within the past two months or ongoing

E3. Previous participation in the EAST trial.

E4. Pregnant women or women of childbearing potential not on adequate birth control: only women with a highly effective method of contraception [oral contraception or intra-uterine device (IUD)] or sterile women can be randomized.

E5. Breastfeeding women.

E6. Drug abuse.

E7. Prior AF ablation or surgical therapy of AF.

E8. Previous therapy failure on amiodarone, e.g. patients who suffered from symptomatic recurrent AF that required escalation of therapy while on amiodarone.

Exclusion criteria related to a cardiac condition

E9. Patients not suitable for rhythm control of AF.

E10. Severe mitral valve stenosis.

E11. Prosthetic mitral valve.

Exclusion criteria based on laboratory abnormalities

E12. Clinically relevant hepatic dysfunction requiring specific therapy.

E13. Clinically manifest thyroid dysfunction requiring therapy. After successful treatment of thyroid dysfunction, patients may be enrolled when their thyroid function is controlled.

E14. Severe renal dysfunction (stage V, requiring or almost requiring dialysis).

6.2.4 Randomisation

The patients will be randomised to one of two parallel study groups, namely "usual care" and "early therapy".

Randomization will be stratified by site to eliminate potential confounders related to different healthcare practice.

A randomisation list will be created by the responsible study statistician.

This list will be imported into the randomisation server of MARVIN. During the trial, randomisation will be performed by MARVIN according to the imported randomisation list. The investigator has to document several clinical items first, because they are of importance for the check of eligibility of the patient for randomisation and for the calculation of the relevant stratum. MARVIN displays the random group and asks for confirmation by authorised study personnel. The account ID of the person performing the randomisation in MARVIN as well as the corresponding time stamp will automatically be documented in an electronic audit trail.

7 Therapy

EAST compares two treatment strategies. None of the therapies employed in EAST is investigational. The difference between treatment groups consists of a different timing of authority approved, marketed and recommended therapies for AF. An early, standardized intervention to maintain sinus rhythm (rhythm control) will be compared with usual care in AF patients.

Since the real distribution of therapeutic strategies in both study groups is unknown, a continuous monitoring of the distribution by the executive Steering Committee will be established. If required, measures will be taken so that therapeutic strategies will remain different in both groups.

Therapies will be administered open-label to achieve a high external validity. All medications and interventional therapies for AF should be documented, including anticoagulation and therapy of concomitant cardiovascular diseases: type of intervention, name of drug, onset of therapy, change in treatment strategy, and reason for change.

7.1 Standard management of AF

Management of AF should follow the recommendations of the 2010 edition of the ESC guidelines for the management of atrial fibrillation (refer to appendix XVIII). This standard management consists of

1. adequate antithrombotic therapy by either continuous therapy with vitamin K antagonists (INR 2-3) or by approved novel anticoagulants such as thrombin inhibitors or factor Xa inhibitors. The choice of agent and monitoring should follow local routine. Adequacy of antithrombotic therapy will be monitored.
2. In patients with AF, ventricular rate should be well controlled. This is usually achieved by a resting heart rate of 80 – 100 bpm. An inadequately controlled ventricular rate should be reduced by AV nodal slowing agents.
3. Furthermore, the recommendations regarding reduction of cardiovascular risk factors should be carefully followed. This includes management of hypertension, diabetes mellitus, vascular heart disease, and heart failure, among others.

To ensure that rate and rhythm control therapy is applied safely, timely and within the current guidelines for AF management, this section of the study protocol details suggested procedures for antiarrhythmic drug therapy and for catheter ablation that are appropriate in the context of early

therapy. When these therapeutic modalities are applied in the conventional care group, the same recommendations apply.

7.2 Recommendations for usual care

Usual care closely follows the suggestions laid out in the current guidelines for AF. In addition to the therapeutic modalities mentioned above, antithrombotic therapy and therapy of underlying heart disease, usual care usually consists of an initial attempt to control symptoms by rate control therapy. Rhythm control interventions are only indicated when symptoms cannot be controlled by optimal rate control therapy in the usual care group.

It is worth to mention that “usual care” in the context of EAST, most often consisting of antithrombotic "background" therapy and rate control, is likely to result in therapeutic improvement for the enrolled patients due to the structured outpatient monitoring of therapy within the trial.

7.3 Recommendations for early therapy of AF

Patients in the early treatment group will be treated on site following exactly the same therapeutic recommendations of the European guidelines as the usual care group. In addition, rhythm control therapy will be initiated early with the aim of preventing recurrence and delaying or preventing progression of AF.

Early-onset rhythm control therapy can consist of the following interventions:

1. Optimal antiarrhythmic drug therapy,
2. Catheter ablation with the aim of pulmonary vein isolation (PVI). This procedure will aim at complete bi-directional isolation of the pulmonary veins,
3. Antiarrhythmic drug therapy and catheter ablation may be supplemented by early cardioversion in patients with persistent AF.

All individual treatment decisions will be taken by the treating study physician considering the labelling of the procedures and drugs and patient preferences. In general, ablation of AF will be a reasonable therapeutic option in symptomatic patients, especially when AF recurs on antiarrhythmic drug therapy. Antiarrhythmic drug therapy is a reasonable initial therapeutic choice in patients with less severe symptoms. Upon AF recurrence, the other therapeutic modality will be added to the existing therapy, i.e. patients on antiarrhythmic drugs will undergo catheter ablation, and patients with AF recurrence after ablation will receive antiarrhythmic drug therapy.

7.3.1 Antiarrhythmic rhythm control drug therapy

Antiarrhythmic drug therapy using ion channel blockers is an essential part of early and comprehensive rhythm control in EAST and should be initiated within two weeks after randomisation latest. Given the fact that recurrent AF may be caused by many different processes, antiarrhythmic drug therapy should not be modified upon the first or second AF recurrence, but should rather be considered a part of a long-term therapy concept. Important for the selection of an antiarrhythmic drug in EAST should be safety concerns. The following antiarrhythmic drugs are suggested for early and safe rhythm control therapy in EAST:

Dronedarone is a novel benzofuran derivative structurally related to amiodarone. It has been approved for treatment of paroxysmal or persistent AF in the US by the Food and Drug Administration and by EMA. The safety profile of dronedarone is advantageous in patients without structural heart disease and in stable patients with heart disease. Specifically, dronedarone has a very low risk for proarrhythmia, e.g. demonstrated by the paucity of torsades de pointes in the EURIDIS/ADONIS and

ATHENA trials. Therefore, dronedarone should be used for early rhythm control in EAST, especially in patients who cannot be treated with sodium channel blockers.

Dronedarone blocks sodium channels, shows a non-competitive anti-adrenergic activity, prolongs action potential duration and refractory periods, and has calcium antagonist properties. Importantly, dronedarone also slows AV nodal conduction (mean decrease in ventricular rate during AF 10-15 bpm). Dronedarone should not be given to patients with left ventricular systolic dysfunction or to patients with current or previous episodes of heart failure, based on the outcomes of the PALLAS and the ANDROMEDA trials.

Dronedarone has a low bioavailability (around 15-20%) and is best absorbed with food. Therefore, dronedarone should be taken concomitant with meals. Dronedarone is given in a fix dose of 2 x 400 mg/d po. If a moderate asymptomatic increase in creatinine is observed after beginning of treatment with dronedarone, the investigator should use clinical judgement taking into account that this may be expected with dronedarone due to reduced tubular creatinine excretion without alterations in glomerular filtration rates. Therefore, depending on patient condition and symptoms, an increase in creatinine should not necessarily lead to the discontinuation of treatment with ACE-inhibitors or angiotensin type II-receptor antagonists, and neither to discontinuation of dronedarone. Given the involvement of the CYP450 3A4 cytochrome in the metabolism of dronedarone, all concomitant drugs which are potent inhibitors of CYP450 3A4 such as ketoconazole, itraconazole, nefazodone, ritonavir, cyclosporin, troleandomycin should be replaced by alternative treatment. It is recommended to monitor the QT interval on the ECG upon initiation of dronedarone therapy. Drugs with a known potential to prolong the QT interval should not be coadministered with dronedarone.

Amiodarone is a multi-channel blocker with a very long half-life (approximately 90 days). The effectiveness of amiodarone to prevent recurrent AF exceeds that of other antiarrhythmic agents. Amiodarone is therefore a good therapeutic option in patients with frequent, symptomatic AF recurrences despite optimal catheter ablation and therapy with dronedarone or other antiarrhythmic drugs. Unlike most other antiarrhythmic drugs, amiodarone can be safely administered in patients with structural heart disease including patients with advanced heart failure. Therefore, amiodarone should be the agent of choice in patients with advanced and unstable heart failure.

The bioavailability of amiodarone amounts to 40%. The main electrophysiological effects of amiodarone are mediated through intracellular metabolites such as desethylamiodarone. Amiodarone contains iodine and is very lipophilic, its volume of distribution is around 5000l. These pharmacokinetic parameters require a prolonged loading dose of amiodarone, and explain the delayed onset of amiodarone' s antiarrhythmic effect. The antiarrhythmic effect is usually only seen after 4-8 weeks of therapy. Amiodarone is excreted via liver and bile.

In the context of the EAST trial, it is recommended to initiate amiodarone therapy by a loading dose of 600 mg/d over four weeks, followed by a lower loading dose of 400 mg/d for four weeks. Thereafter, the maintenance dose of 200 mg/d should be continued. This regime follows the loading doses used in the EMIAT trial.

During therapy, ECG monitoring is suggested: Amiodarone slows AV nodal conduction. Therefore, heart rate during AF and PQ interval should be monitored during initiation of therapy, and rate control therapy should be adapted according to the measurements. Furthermore, an increase in QT interval beyond 0.076 s upon therapy should raise caution for possible proarrhythmic effects. Amiodarone has important drug-drug interactions: The required dose of vitamin K antagonists to achieve a therapeutic INR is lower when amiodarone is concomitantly given, due to an altered metabolism of warfarin or phenoprocoumon. In addition, drugs with a known potential to prolong the QT interval should not be co-administered with amiodarone.

Table 1: Suggested doses and main caveats for dronedarone and amiodarone

**Drug**	**Dose**	**Caveats for use**	**ECG monitoring**	**AV nodal slowing**
Dronedarone	2 x 400 mg/d	concomitant medication with QT-prolonging drugs, can be used in light-moderate heart failure, increases creatinine excretion – elevations in serum creatinine of 0.1-0.2 mg/dl are common and do NOT reflect reduced renal function. Dronedarone should not be used in patients with heart failure.	QT interval prolongation > 0.06s upon therapy	10-12 bpm in AF
Amiodarone	600 mg/d for 4 weeks, 400 mg/d for 4 weeks, then 200 mg/d	concomitant medication with QT-prolonging drugs, can be used in heart failure, reduce dosing of vitamin K antago-nists and of digitoxin/digoxin	QT interval prolongation > 0.06s upon therapy	10-12 bpm in AF

Other antiarrhythmic drugs may be used in selected patients, provided that the investigator can assure the safety of such therapy. These may be needed in patients in whom even a combination of dronedarone and catheter ablation cannot achieve adequate symptomatic improvement in patients with symptomatic AF recurrences, especially when amiodarone is not an option, e.g. due to extra-cardiac side effects. It may also apply in patients in whom dronedarone is considered second-line therapy. In these special circumstances, and provided that study patients do not suffer from structural heart disease, flecainide and propafenone may be used.

Flecainide is a slowly dissociating sodium channel blocker. It can be safely administered in patients without relevant structural heart disease. This can usually be documented by a normal left ventricular wall thickness and left ventricular ejection fraction on two-dimensional echocardiography and a normal stress test (treadmill, stress echocardiography, or myocardial scintigraphy). Flecainide should not be used in patients with coronary artery disease or in patients with a reduced left ventricular ejection fraction.

The usual dose for flecainide is 2 x 100 mg/d per os. Its bioavailability is almost complete. Flecainide's serum half-life is around 16 – 20 hours. In patients who weigh more than 80 kg, especially when AF recurs, the dose can often be safely increased to 3 x 100 mg/d po. Flecainide is eliminated by both renal excretion after hepatic metabolization via the CYP450-2D6 isoenzyme, rendering flecainide difficult to douse in patients with a reduced renal function: Patients with a glomerular filtration rate < 50 ml/min as calculated using the Cockroft-Gould formula, or roughly estimated by an increased serum creatinine level, should not receive flecainide.

Upon initiation of flecainide therapy, daily ECG monitoring is recommended. An increase in QRS duration of > 25% on therapy compared to baseline is an important sign for a potential risk for proarrhythmia. Patients who experience a prolongation of QRS duration > 25% during flecainide therapy should not continue such therapy. When the flecainide dose is increased, QRS duration should be monitored following the same criteria. In many countries, it is relatively simple to measure flecainide plasma levels. These should be measured as trough levels, e.g. in the morning prior to intake of the morning tablet.

Propafenone is another slowly dissociating sodium channel blocker. In addition, propafenone has a weak ß adrenoreceptor blocking effect. This effect is often not measurable by clinical means (e.g. mean heart rate during AF). It can be safely administered in patients without relevant structural heart disease. This can usually be documented by a normal left ventricular wall thickness and left ventricular ejection fraction on two-dimensional echocardiography and a normal stress test (treadmill, stress echocardiography, or myocardial scintigraphy). Propafenone should not be used in patients with coronary artery disease or in patients with a reduced left ventricular ejection fraction.

Propafenone' s serum half-life is around 5 hours. Its bioavailability amounts to 50%. Propafenone is highly protein-bound. Propafenone is mainly metabolised in the liver via the CYP4502D6 enzyme. Poor metabolizers may experience excessive plasma levels of propafenone upon exposure to normal drug doses. The usual oral dose is 3-4 x 150 mg/d. Similar to flecainide, daily ECG monitoring is recommended upon initiation of therapy for 2-3 days. An increase in QRS duration of > 25% on therapy compared to baseline is an important sign for a potential risk for proarrhythmia. Patients who experience a prolongation of QRS duration > 25% during propafenone therapy should not continue such therapy. More than 600 mg daily dose of propafenone are not recommended.

Table 2: Suggested doses and main caveats for antiarrhythmic drugs in the EAST trial

**Drug**	**Dose**	**Caveats for use**	**ECG monitoring**	**AV nodal slowing**
Flecainide	2-3 x 100 mg/d	creatinine clearance < 50 mg/ml, coronary artery disease, reduced LVEF	QRS duration increase > 25% above baseline	-
Propafenone	3-4 x 150 mg/d	coronary artery disease, reduced LVEF	QRS duration increase > 25% above baseline	slight

7.3.2 Rate control therapy

All patients should additionally receive adequate rate control therapy, usually consisting of ß- blockers, calcium channel antagonists, and digitalis glycosides. Dose should be adjusted to achieve a resting heart rate below 100 bpm. In some patients, a lenient rate control target may be sufficient [40, 41]. In others, rate control may also consist of achieving an adequate increase in heart rate upon exertion. Heart rate upon exertion can be assessed by a 6 minute walk test which is part of the baseline and follow-up assessments of patients participating in a sub-study. Dosage of rate control therapies should be titrated to avoid symptomatic bradycardias [40, 41].

Table 3: Suggested daily doses for rate control agents. These drugs are readily available and used in all study sites. The aim of rate control is adequate control of ventricular rate during AF.

Metoprolol	100 – 200 mg/d (often 3 x 47.5 mg/d) po
Bisoprolol	5 – 10 mg/d po
Digoxin	0.2 - 0,25 mg/d maintenance dose, loading is usually required for 3-7 days
Digitoxin	0.07 mg/d po maintenance dose, loading is usually required for 3-7 days
Verapamil	3 x 80 mg/d po, no loading dose required

7.4 Concomitant medication

No patient must be deprived of any necessary therapy as a consequence of participating in EAST. It is important that the participants receive all accepted evidence-based treatments in accordance with national or international guidelines. This includes optimized antithrombotic therapy in patients at risk for stroke, optimal treatment of arterial hypertension, preferentially with an ACE inhibitor or a sartan, adequate heart failure treatment including diuretics, ACE inhibitors or angiotensin type II-receptor antagonists (sartans), beta-blockers, medication for rate control, and adequate therapy of vascular disease.

ß-adrenoreceptor blockers (ß-blockers) are permitted (except sotalol that is not considered as ß-blocker but as antiarrhythmic drug), but should be used with caution. Dronedarone, propafenone and amiodarone have AV-nodal slowing properties in addition to their antifibrillatory effects. Therefore, the ß-blocker dose should be adapted to achieve adequate rate during AF if given concomitantly with dronedarone, propafenone or amiodarone. The dose of other rate-controlling agents such should be adjusted if needed. Dronedarone may increase plasma levels of digoxin. Therefore, it should be expected that patients could require and tolerate lower doses of digoxin than usual.

All concomitant drugs which can cause torsades de pointes are contraindicated when amiodarone or dronedarone are given. Such drugs include some phenothiazines, cisapride, bepridil, tricyclic antidepressants, and certain oral macrolides. A full list of drugs is accessible at www.torsades.org

7.5 Cardioversion

Cardioversion will be performed in patients with persistent AF following local routine. It is desired within the concept of early therapy to achieve cardioversion early, e.g. using guidance by transeosophageal echocardiography rather than several weeks of anticoagulation. Schedules for cardioversion in the usual care group, if indicated according to the 2010 ESC Guidelines, should be defined by local routine.

7.6 Catheter ablation

The aim of catheter ablation in AF patients is **bi-directional isolation of the pulmonary veins (PVI)**. PVI should be performed following local routine. Clearly, procedural safety is paramount in the context of early therapy, and all means for a safe procedure should be taken. The study will be conducted in experienced centres which should be on the plateau phase of the learning curve for AF ablation. Evaluation of experimental or novel ablation devices is not permitted in this study.

The exact ablation technique should follow the recommendations of the AFNET/EHRA/ECAS consensus statement on catheter ablation of atrial fibrillation, the upcoming ESC guidelines on AF management, and local routine. Local routine should guide details of the procedure (e.g. the type of ablation and mapping system used, or the choice of ablation energy). An Ablation Committee will provide an evidence-based list of reasonable devices for PVI (catheters, energy sources, catheter visualization and mapping systems). In selected centres, other procedures and technologies may be used after review of efficacy and safety data by that sub-committee.

Prior to each catheter ablation procedure, a transoesophageal echocardiography or another method to exclude left atrial thrombi should be applied. Usually, isolation of the pulmonary veins will be achieved by circumferential, often antral, isolation of the left and right pulmonary veins in “two circles”. Sequential isolation of the ostia of each pulmonary vein is also permitted if the operator deems this procedure appropriate for a given patient. Usually, successful isolation will be demonstrated by abolition of conduction of atrial impulse into the pulmonary veins. After each ablation procedure, a three-months “healing period” should be observed. During this healing period, repeated catheter

ablation procedures are not allowed. In case of recurrent AF, appropriate antiarrhythmic drug therapy should be initiated.

In patients of the early therapy group, each ablation procedure should be performed not later than two months after its indication, i. e. two months after randomisation or two months after AF recurrence requiring escalation of rhythm control therapy.

7.6.1 Re-ablation with the aim to re-isolate the pulmonary veins

Upon AF recurrence outside of the therapy stabilization period despite additional antiarrhythmic drug therapy, or upon recurrence of arrhythmias that are likely amenable to catheter ablation (e.g. isthmus-dependent or left atrial flutter), re-ablation should be performed. Similar to the first procedure, in patients of the early therapy group, re-ablation should be performed as early as possible, and no later than 2 months after documentation of recurrent arrhythmias. It is well documented that electrical re-connection of the pulmonary veins is common after initially successful isolation of the pulmonary veins. Therefore, the first aim of any re-ablation procedure should be verification of the electrical isolation of pulmonary veins, and re-isolation should be performed following local routine (usually by gap-mapping of the previously applied isolation lines). Re-isolation of pulmonary veins should be verified electrically by monitoring electrical activity in the pulmonary veins.

7.6.2 Other ablation targets

Ablation techniques beyond successful and persistent isolation of the pulmonary veins should be reserved to patients with recurrent AF despite continued antiarrhythmic drug therapy or to patients with atrial tachycardias or atrial flutters. Such additional techniques should follow local routine. Unlike re-isolation of the pulmonary veins, application of such additional ablation techniques constitutes a change in therapy in the context of EAST. They may include the techniques outlined in the catheter ablation consensus document and may include

- A linear lesion that connects the mitral anulus and the isolation line around the pulmonary veins (“mitral isthmus line”)
- A linear lesion that connects the lateral and septal isolation lines around the pulmonary veins (“roof line”)
- Ablation of the isthmus between the vena cava inferior and the tricuspid anulus (“right atrial isthmus line”)
- Targeted ablation of left or right atrial tachycardias following established mapping and ablation techniques
- Ablation of continuous fractionated electrograms (CFAE), electrical isolation of the coronary sinus, or electrical isolation of the superior vena cava in selected patients as a last resort

This suggestion of procedures closely follows the “step-wise approach” suggested by the group of Michel Haissaguerre.

7.7 Post-study treatment

After end of the study (planned or premature study discontinuation), the investigator is free to decide, on which further medication to put the patient or which procedure to be performed.

8 Assessing and Reporting of Adverse Events

As all treatments in EAST are in-line with clinical practice and recommended by guidelines, adverse events are expected to occur in similar clinical manifestations and at a comparable rate as the known adverse events of the approved therapies applied in the trial. Therefore, in the context of EAST, not all non-serious adverse events will be recorded, but only non-serious "Adverse Events of Special Interest" which are defined as described below.

8.1 Adverse Events of Special Interest

Proarrhythmia: Any event that is an arrhythmia or has a potential arrhythmic background and is additionally judged as causally related to the therapeutic intervention, e.g. drug-induced proarrhythmia (torsade de pointes, ventricular tachycardia, ventricular fibrillation), drug-induced bradycardia, AV nodal block, ablation-induced or drug-induced atrial arrhythmias (e.g. left atrial flutter), or syncope.

Other complication of therapy: Any other event that is judged as causally related to the therapies applied within the trial, e.g. bleeding events caused by catheter interventions or antithrombotic therapy, complications of ablation procedures (e.g. pulmonary vein stenosis, pericardial tamponade, atrio-oesophageal fistula), drug toxicity of AF-related drug therapy, among others.

Other non-serious adverse events will not be recorded in EAST.

8.2 Serious Adverse Events

A **serious adverse event (SAE)** is any untoward medical occurrence that

- results in death or,
- is life-threatening or,
- requires inpatient hospitalization or prolongation of existing hospitalization or,
- results in persistent or significant disability/ incapacity or,
- is a congenital anomaly / birth defect, or
- is a medically important event.

More than one of the above criteria can be applicable to each event.

Medical and scientific judgement should be exercised in deciding whether expedited reporting is appropriate in other situations, such as important medical events that may not be immediately life-threatening or result in death or hospitalization but may jeopardize the patient or may require intervention to prevent one of the other outcomes listed in the definition above. These should also usually be considered serious.

NOTE:

Death is the outcome of an Adverse Event. The event to be reported comprehensively is the medical condition leading to death, e.g. underlying disease, accident. The only exception is sudden cardiac death. The medical records will be used to determine whether death was due to a cardiovascular cause and/or due to AF [11].

Life-threatening in the definition of a Serious Adverse Event or Adverse Reaction refers to an event in which the patient was at risk of death at the time of the event. It does not refer to an event which hypothetically might have caused death if it was more severe.

Hospitalisation is defined as inpatient care of more than one calendar day (= at least one overnight stay).

As a study specific definition,
a) any hospital stay planned prior to randomisation and
b) any overnight hospital stay required only for diagnostic procedure (e.g. sleep laboratory)
is not considered "hospitalisation" in the sense of the criteria for Serious Adverse Events and needs not be documented unless any other criteria for seriousness is met.

A catheter ablation for AF (if performed during a hospitalisation) is **not** considered as "hospitalisation" in the sense of the criteria for Serious Adverse Events as it is part of the therapy within the context of the EAST study. A catheter ablation for AF will be documented in the eCRF as extra “visit” but needs not be documented as Serious Adverse Event unless any other criteria for seriousness is met.

8.3 Recording and Reporting Serious Adverse Events and Adverse Events of Special Interest (if serious)

All serious adverse events will be recorded in the e-CRF.

The investigator should specify and report in the e-CRF the nature of the sign or symptom, the date of onset, the date of resolution (duration), the intensity, interventions performed (if any), the relationship to study treatment and to AF, and the outcome.

In the case of knowledge of a serious adverse event, the investigator must immediately (within one working day of being notified of the event):

- Fill out as a minimum the following items of the internet-based SAE report:
  - type of event,
  - description (if mandatory),
  - date of onset,
  - criteria for seriousness,
  - causal relationship to study therapy.
- As soon as further information regarding the event is available (e.g. discharge letter), the investigator should complete the documentation in the e-CRF and sign it electronically. Copies of the discharge letter, of all reports regarding examinations carried out and/or diagnostic findings should be faxed to CRI. For laboratory results, the laboratory normal ranges should be included.
- Follow-up of any SAE that is fatal or life threatening should be provided within one additional calendar week.

According to legal requirements and international standards, annual safety reports will be prepared by CRI and forwarded to responsible authorities of all participating countries and to all corresponding ECs / IRBs.

8.3.1 Definition of Intensity

Intensity	Definition
Mild	Patient is aware of signs and symptoms but they are easily tolerated
Moderate	Signs/symptoms cause sufficient discomfort to interfere with usual activities
Severe	Patient is incapable to work or perform usual activities
Life-threatening	Patient is at severe risk of death at the time of the event

8.3.2 Definition of Causality

In accordance with the CIOMS group, causality of an event will be assessed as:

not related: No causal relationship exists between the study treatment and the event but an obvious alternative cause exists, e.g. the patient's underlying medical condition or concomitant therapy.

or

related: There is a reasonable / plausible possibility that the event may have been caused by the study treatment (e.g. the event cannot be explained by concomitant disease(s) or other drugs/treatments).

8.3.3 Adverse Event Follow-up Procedures

The investigator should take all appropriate measures to ensure the safety of the patients, notably he/ she should follow-up the outcome of any AE of special interest or SAE (clinical signs, laboratory values or other, etc.) until the return to normal or consolidation of the patient's condition.

In case of any serious adverse event, the patient must be followed until clinical recovery is completed and laboratory results have returned to normal, or until progression has been stabilized. This may imply that follow-up will continue after termination of the EAST trial, and that additional investigations may be requested by the monitoring team.

8.3.4 Suspected Unexpected Serious Adverse Events (SUSARs)

This applies to any serious adverse event that is considered related to study therapy and the nature or severity is not consistent with the applicable product information. The expectedness of an adverse reaction will be determined by the sponsor / ERC according to the respective summary of product information in its current version.

CRI will notify the competent authorities, corresponding ECs / IRBs and all local principal investigators concerned of SUSARs in-line with applicable regulatory requirements.

8.3.5 Pregnancy

It is reminded that all means should be put in place to prevent pregnancy during the study. Nevertheless, in case of pregnancy, the sponsor should be immediately informed. Follow-up of the pregnancy will be mandatory until the outcome has been determined. Pregnancy will be recorded as an AE in all cases. It will be qualified as a SAE only if it fulfils SAE criteria.

9 Study Schedule

9.1 Visit schedule

The timing and assessments of the study procedures are summarized in the following table.

Table 4: Visit Schedule

**Assessment**	**Baseline Visit** Day 0	**Central FU*** Month 6	**Visit 1*** Month 12	**Central FU*** Month 18	**Visit 2*** Month 24	**Central FU*** Month 30	**Central FU*** Month 36	**Central FU*** Month 42 [2]
Signed ICF (medical informed consent)	X							
Check inclusion & exclusion criteria	X							
Physical examination (PE)/ medical history	X		X		X			
12-lead ECG	X		X		X			
Laboratory parameters (blood sample)	X		X [3]		X			
Transthoracic echocardiography (TTE)	X				X			
Initiation of therapy (early rhythm control or usual care) [1]	X							
Karnofsky score	X		X		X			
MoCA	X				X			
Quality of Life (EQ-5D, SF-12)	X				X			
Adverse event/ serious adverse event		X	X	X	X	X	X	X

[1] In patients with persistent AF, an early cardioversion will be performed.

[2] Further central follow-up is planned in 6-monthly intervals until the end of the whole trial.

[3] Only INR / PT and alpha-PTT

* Time window +/- 2 months

9.2 Baseline Visit

A patient meets eligibility criteria of the study if all inclusion and exclusion criteria are fulfilled as described in section 6.2.2 and 6.2.3. Prior to any trial related procedure a signed informed consent form has to be obtained from every patient to be included in EAST and kept on file locally.

At the baseline visit, the investigator or designee will:

- Obtain patients' informed consent
- Assess patients' medical history
- Obtain a 12-lead ECG (an ECG performed within 14 days prior to randomisation might be used as baseline ECG)
- Perform a physical examination
- Obtain blood samples for laboratory assessments (refer to section 9.7)
- Conduct the 6-minute walk test (only sites participating in this sub-study)
- Assess cognitive function (MoCA test)
- Assess social functioning (EQ-5D and SF-12 questionnaire)
- Assess performance status (Karnofsky score)
- Perform a transthoracic echocardiography (a TTE performed within 4 weeks prior to randomisation might be used as baseline TTE)
- Initiate or schedule study therapy
- Only for patients randomised to the early therapy group: hand out an ECG device (refer to section 9.8)

9.3 Follow-up

Information regarding study-relevant outcomes / events will be obtained by questionnaires in 6-monthly intervals, starting at month 6 until month 90 or longer if necessary (i.e. central follow-up). Personal follow-up visits will be performed at months 12 and 24 only (instead of the central follow-up) as study-relevant technical measurements and health-economic information are expected to change only little in the long-term follow-up after three years.

All patients will be followed until completion of the total trial for outcome and safety. As some outcome events (e.g. stroke or myocardial infarction) are not directly related to the trial intervention, we encourage adherence to the assigned therapy group even after a primary outcome event occurred.

For all follow-up visits, a time window of +/- 2 months is allowed.

9.3.1 Clinical Visits (Months 12, 24)

At each visit, the investigator or designee will

- Obtain a 12-lead ECG
- Obtain a Holter ECG (only sites participating in this sub-study)
- Perform a physical examination
- Obtain blood samples for anticoagulation status and liver function tests as far as the patient is prescribed dronedarone (refer to section 9.7)
- Conduct the 6-minute walk test (only sites participating in this sub-study)
- Assess performance status (Karnofsky score)
- Assess for clinical events and AEs respectively SAEs occurred since the preceding visit / contact

At month 24, the investigator or designee will in addition:

- Assess social functioning (EQ-5D and SF-12 questionnaire)
- Assess cognitive function (MoCA test)
- Perform a transthoracic echocardiography
- Obtain blood samples for assessment of serum creatinine, haemoglobin, leucocytes, platelets, and coagulation status

9.3.2 Central Follow-up

Questionnaires asking for clinical events, respectively SAEs, since last contact will be prepared by CRI and sent by the study site by mail to all patients. In case of missing answer after one written reminder (prepared by CRI, posted by the study site), the study site should contact the patient by phone in order to obtain the required information.

The patients will return the completed questionnaires (which contain pseudonymous data only) directly to CRI. In case a patient reports an event, the responsible study site will be informed by CRI and has to contact the patient's family doctor respectively the hospital where the patient was treated and ask for supportive documents (i.e. hospital discharge letter, diagnostic reports) as applicable.

Data regarding events and hospitalisations reported by the patient will be documented primarily by CRI in the e-CRF on the basis of available medical data. The study site is subsequently responsible for completion of the event data.

9.3.3 Triggered Visits

In case AF recurrence is detected in an ECG and the decision is taken for an escalation in therapy, a triggered visit should be scheduled and therapeutic measures should be documented in the eCRF. Escalation in therapy does not include change of dosage or change of antiarrhythmic drug within the same substance class (e.g. flecainide to propafenone). During these triggered visits, the investigator or his designee will:

- Obtain a standard 12-lead ECG and evaluate AF and type of AF
- Assess for clinical events and AEs respectively SAEs occurred since the preceding visit / contact

9.4 Contact at (premature) study termination

In case of patient's withdrawal of consent to further study participation, the investigator should contact the patient and

- assess for interventional treatment for AF and
- assess for clinical events and AEs respectively SAEs that occurred since the preceding visit / contact.

These data will be documented in the e-CRF in a withdrawal visit.

At the end of the total trial a final questionnaire will be prepared by CRI and sent by the study site to all patients still under observation, asking for clinical events respectively SAEs since the last contact.

Wherever feasible, patients will be followed for all-cause mortality after completion of the trial, e.g. via central health care registries.

9.5 Transthoracic Echocardiography

Left ventricular function will be measured by M-mode echocardiography as left ventricular end-diastolic and end-systolic diameter and fractional shortening. These measurements may be

supplemented by left ventricular end-diastolic and end-systolic volume as estimated by the Simpson (biplane) method. For patients in AF, both M-mode and 2-D measurements will be averaged over 5 cardiac cycles.

9.6 Electrocardiogram

All patients will undergo 12-lead ECG at every clinical follow-up visit. Operators recording ECGs should ensure that chest leads are placed in the proper position and electrodes make good skin contact to minimize artefacts. The reversal of limb leads and the switching of precordial leads have been well-documented to cause alterations in ECGs.

Calibration marks or clear notations should be inscribed on each ECG tracing to enable the interpreter to determine the paper speed and gain settings used in recording. Standard settings of a paper speed of 50 mm per sec and a calibration of 10 mm per mV should be used unless required by technical reasons and indicated on the tracing. An adequate notation of date and time should also be available.

12-lead ECGs performed at the baseline visit and at follow-up visits preferentially should be available in digitised format in order to enable simple uploading in the eTMS (MARVIN). In case an ECG is only available as paper version, a copy has to be faxed to CRI for further analyses to be performed by a central evaluation office.

9.7 Blood Samples

Routine laboratory parameters will be assessed at baseline visit in order to determine the current laboratory status . If these parameters can be assessed from a blood sample not older than 7 days at the date of inclusion, the blood sampling does not have to be repeated. Parameters include serum creatinine, haemoglobin, leucocytes, platelets, the coagulation status, and cardiac enzymes (CK, CK-MB, troponin) in case of suspicious ACS. For patients prescribed dronedarone, liver function tests (i.e. ALAT) should be performed at baseline visit and at every follow-up visit as recommended in the summary of product characteristics.

All blood parameters will be determined at the local laboratory of the study sites provided their analytical laboratories are certified. Copies of the laboratory certificates as well as the corresponding normal ranges (as far as necessary) will be collected by CRI as a part of the site initiation procedure.

At baseline, an additional blood sample will be collected (20 ml whole blood) and sent to a central laboratory for further analysis. Patients have to provide explicit signed informed consent to obtain this extra blood sample. Details regarding handling and shipment of these blood samples are described in a separate lab manual.

Aim of theses analyses is to evaluate the possible mechanisms of AF genesis and to gain new knowledge regarding treatment of AF in the future.

9.8 ECG monitoring

At the baseline visit every patient in the early therapy group will be handed out an ECG device and asked to record his/her ECG daily (at least 2-3 times a week) irrespective of related symptoms at varying times of the day. In case of symptoms the patient should additionally record an ECG. Details are described in appendix XV.

9.9 Sub-Studies

For a subgroup of patients, brain morphology on MRI will be determined in a planned sub-study that will relate cognitive function with therapy, rhythm, and cerebral lesions. These patients have to provide a separate signed informed consent form in addition to the informed consent of the main study. Details will be described in separate protocols. Other envisioned sub-studies encompass e.g. measurement of left atrial function by three-dimensional echocardiography and intensified ECG monitoring. Sub-studies requiring additional examinations that are outside of routine care for AF patients (e.g. MRI) will be subject to separate informed consent.

10 Duration of study participation

10.1 Overall Duration of Study

With an expected screening and enrolment period of 65 months and a sliding initiation of sites over a period of 18 months, and a minimum follow-up period of another 30 months, overall study duration is calculated to be approximately 8 years (95 months). The end of the study will be established, when the number of primary outcomes for final analysis has been reached (refer to section 12). This will be defined by the eSC based on the information provided by CRI and the study statistician. Final data cleaning will require presumably two more months after study closure.

10.2 Individual duration of Study

According to the study protocol, follow-up is planned in 6-monthly intervals after enrolment until the end of the study. It is expected that the mean follow-up time will be about five years per patient with a minimum follow-up time of 30 months and a maximum follow-up time of presumably approximately 8 years (95 months). Every patient will be followed-up until the end of the entire study.

11 Stopping and Discontinuation Criteria

When the study is terminated, the nature of termination will be documented (scheduled end/discontinuation with justification). Discontinuation of the study will be communicated in writing and will be a joint decision of the sponsor and the EC.

11.1 Discontinuation criteria related to the study

Following a recommendation of the DSMB, the eSC may decide discontinuation of the study due to efficacy criteria or adverse reactions in either study group. Discontinuation of the study can also be decided if patients cannot be recruited in sufficient numbers within a certain time period. This also applies to local study sites which may be closed if major protocol violations occur repetitively, the site does not comply with the study protocol or decisions of the committees or the principal investigator or if the site remains inactive for a long period of time.

11.2 Discontinuation criteria related to the patient

The patients will be advised in the informed consent forms that they have the right to withdraw from study participation at any time without statement of reasons.

Patients who withdraw consent to participation in the trial will be contacted by the local investigators. If they consent to a minimal follow-up for vital status or for the primary outcome (either by central follow-up via questionnaires and contacting of the patient's family doctor or by unique contact at the end of the whole study), these data will be recorded in a withdrawal visit.

In any case, patient's withdrawal of consent has to be documented and confirmed either by the patient's (preferably) or the investigator's signature.

In the event that a patient completely withdraws from the study (i. e. no further contact allowed), the date and the reason of the individual study termination should be recorded in the withdrawal visit.

The responsible investigator will take all acceptable measures to retrieve information on vital status on all patients enrolled in the trial.

Once a patient has been randomised and an attempt was made to treat the patient, the treatment of the patient must not be discontinued. In case of patient's request or medical necessity of study treatment discontinuation, patients will be followed according to study protocol. In case a protocol deviation or violation is noticed the patient will remain in the intention-to-treat group and will be followed according to protocol. Patient will be followed according to the study protocol irrespective of whether they experience a primary outcome.

Reasonable effort should be made to contact any patient lost to follow-up during the course of the study in order to complete assessments and retrieve any outstanding data.

12 Statistics

12.1 Statistical Methods

12.1.1 Analysis of the primary outcome

The primary analysis follows from the chosen group sequential approach. Up to four analyses will be performed to the end of the trial. Each analysis will consist of a log-rank test comparing the 1st co-primary outcome "time to the first primary event" between random groups. Significance bounds are given in the Appendix XI "Design and sample size calculation". Overall, an alpha of 0.04 will be spent on the 1st co-primary outcome. The 2nd co-primary outcome will be compared between random groups by means of a Mann-Whitney U test. An alpha of 0.01 will be spent on the 2nd co-primary outcome.

A more detailed extended analysis of the primary outcome will be performed after termination of the study by statistical model building based on Cox proportional hazard models that include several covariates. In particular, pathways, type of recruiting site, age, gender, clinical conditions, and other baseline determinations will be taken into account. Interventions will be included as time-dependent covariates.

12.1.2 Analysis of secondary outcomes

Secondary outcomes will be analysed depending on the scale type. Time-to-event outcomes will be analysed by log-rank tests. Dichotomous variables will be tested by Likelihood-ratio-chi-square tests. Continuous variables will be analysed with analysis of covariance models that include the baseline determinations of the outcome, if appropriate, and with two-sample t tests in all other cases. Scale transformations will be considered based on the pooled data set. Analysis details will be laid down in a statistical analysis plan that has to be finished and agreed upon within the executive Steering Committee before breaking the blind. Tests will be performed on a 5% level without multiplicity adjustment to yield nominal p values.

12.1.3 Subgroup analyses

Subgroup analyses that allow to identify determinants of treatment success will be performed by interaction tests. A list of subgroup criteria will be pre-specified in the statistical analysis plan.

12.1.4 Safety analyses

Primary and secondary safety outcome and adverse event frequencies will be compared between groups according to type of event and organ. All interventions planned in EAST are in-line therapies. EAST does not test a specific type of therapy, but rather a novel therapeutic strategy, namely early rhythm control therapy. In addition to the strategy-oriented group comparisons, complication rates will be listed according to type of intervention across groups.

12.2 Sample Size Calculation

Usual-care therapy is estimated to result in an annual death rate of 3-4%, an annual stroke rate of 1.5%, and an annual rate of cardiovascular hospitalizations (excluding planned hospitalizations for AF) of 3%, based on controlled trials [7, 11, 12, 34, 35]. In “real life”, these rates are likely to be higher [2, 21]. All available data suggest that a relevant part of these events is due to the presence of AF. We expect an annual event rate of 8%[7, 11, 12, 34, 35] for the primary outcome consisting of cardiovascular death (2.5%), stroke (1.5%), myocardial infarction (1%), heart failure (3%). Further, a 20% reduction of the hazard rate was judged to be clinically relevant and to be expected if the theoretical assumptions on the intervention apply.

Based on these assumptions and further assumptions on recruitment and on follow-up as defined before, a sample size calculation for a group sequential design with four stages was performed resulting in a required recruitment of 2,745 patients at a rate of 42 pts/month to compensate a loss-to-follow-up of 5% of the observation time to keep an overall alpha level of 5% two-sided and to reach a power of 80%. Details are given in Appendix XI.

As long as the expected reduction of the hazard rate is kept constant at 20%, modifications of the number of patients to be recruited, the recruitment time or the follow-up time are possible without thread of the validity of the trial as long as the blind is not broken and the first stage of the group sequential design is not reached. Thus, a re-assessment of the sample size based on blind information only is provided after 42 months or when 2,400 patients have been included, whatever is first. This re-assessment will be performed by the trial statistician as follows: based on the original assumptions of the model, the number of events E that should have been observed up to the index date of the reassessment is calculated. If O is the real observed number and h_c is the control group hazard rate originally assumed, a new sample size calculation will be performed based on a control group hazard rate of $h_C \cdot O/E$, but with an assumed hazard rate reduction of 20% in the intervention group.

12.3 Patient Selection for Analyses

The primary analysis is in the intention-to-treat (ITT) population, consisting of all randomized patients with at least one follow-up assessment. The more detailed extended analysis of the primary outcome will be performed in the same population; If missing values in covariates are present, a multiple imputation procedure (20 repetitions) will be performed in the pooled data set using baseline characteristics of the patients.

After a blind review of the database, the eSC members will decide which of the reported protocol violations were considered major with potential impact on study results. For an additional evaluation of

the primary outcome, the primary and extended primary analysis will be repeated in the subgroup of patients without major protocol violations (per protocol (PP) population).

Analyses of secondary outcomes will take place in the same analysis subsets as the primary outcome. If outcomes are missing, different imputation methods will be performed (multiple imputation, interpolation, last observation carried forward) to demonstrate the sensitivity of the results for assumptions.

The safety analysis will be performed in the ITT population from randomization as treatment strategies are to be compared with respect to safety.

13 Access to Source Data / Documents

13.1 Source Data

Source data are defined as all information in original records and certified copies of original records of clinical findings, observations or other activities in a clinical study necessary for the reconstruction and evaluation of the study. Source data are contained in source documents (original records or certified copies).

13.2 Source Documents

Source documents are defined as original documents, data and records (e.g. hospital records, clinical and office charts, electronic patient records, laboratory notes, memoranda, patient diaries or evaluation check lists, pharmacy dispensing records, recorded data from automated instruments, copies or transcriptions certified after verification as being accurate copies, microfiches, photographic negatives, microfilm or magnetic media, x-rays, patient files, records kept at pharmacy, at the laboratories and at medico technical departments) involved in this clinical study.

In case of data that are result of patient interrogation and will not be documented in clinical routine, the e-CRF is the source document, if the patients answer is documented there without prior documentation on paper (e.g. in case of central follow-up performed by questionnaires).

13.3 Direct Access

Direct access is defined as the permission to examine, analyse, verify and reproduce any records and reports that are important to evaluation of a clinical study. Any party with direct access should take all reasonable precautions within the constraints of the applicable regulatory requirements to maintain the confidentiality of patient identities and sponsor proprietary information.

The investigator agrees that representatives or the designees of the sponsor such as monitors and auditors, and appropriate Regulatory Agencies will be given direct access to the regular clinical files of the patient.

14 Quality Control and Quality Assurance

14.1 Quality Control

Quality Control is defined as the operational techniques and activities, such as monitoring, undertaken within the quality assurance system to verify that the requirements for quality of the study related activities have been fulfilled.

Quality Control should be applied to each stage of data handling to ensure that all data are reliable and have been processed correctly.

14.2 Initiation Visit

At each site an initiation visit will be performed by a representative of CRI before enrolment of the first patient at this site.

14.3 Study Monitoring

Authorized, qualified representatives of CRI will accomplish the monitoring of the study sites during the trial.

Data of a sufficient number of patients will be verified on site by source data validation checks for outcome and compliance with the protocol and consistency with data in the e-CRF.

It is important that the investigator and relevant personnel are available during the monitoring visits and that an appropriate location and sufficient amount of time is devoted to the process. During the monitoring visit a PC with internet connection should be available to the monitor for direct connection to the internet database of the study and to all the data of the patients if stored in the data system of the hospital or catheter lab.

The main duty of the monitor is to help the sponsor and the investigator to maintain a high level of ethical, scientific, technical and regulatory quality in all aspects of the trial. At regular intervals during the study, the local site will be contacted through monitoring visits, letters/ emails or telephone calls by a monitor to review the progress of the study.

Further details are described in a separate monitoring manual.

14.4 Close Out Visit

Independent close out visits are not planned. In case of special requests by the sponsor, a separate close out visit may be performed at the end of the trial. The close out visit may be combined with the last monitoring visit.

14.5 Quality Assurance

Quality Assurance is defined as the planned and systematic actions that are established to ensure that the study is performed and the data are generated, documented (recorded) and reported in compliance with Good Clinical Practice (GCP) and the applicable regulatory requirements.

The investigator should permit auditing by or on the behalf of the sponsor and inspection by applicable regulatory authorities. The investigator shall take appropriate measures required by the sponsor to take corrective actions for all problems found during the audit or inspections.

14.5.1 Inspections

An Inspection is defined as the act by a regulatory authority of conducting an official review of documents, facilities, records and any other resources that are deemed by the authorities to be related to the clinical study and that may be located at the site of the study, or at the Sponsors and/or clinical research organisation facilities or at any other establishments deemed appropriate by the regulatory authorities.

14.5.2 Audits

An audit is a systematic and independent review of study related activities and documents to determine whether the validated study related activities were conducted and the data were recorded, analysed and accurately reported according to the protocol, designated Standard Operating Procedure (SOP), Good Clinical Practice (GCP) and the applicable regulatory requirements. An independent audit at the study site may take place at any time during or after the study.

15 Ethical and Legal Consideration

This is an investigator driven, phase IV trial (proof-of-principle to test the usefulness of the new therapeutic concept "early rhythm control therapy") which meets all relevant ethical and regulatory standards (ICH-GCP and EU-Directive 2001/20/EC). The trial will be conducted in accordance with the principles laid down by the 18th World Medical Assembly (Helsinki, 1964) and all applicable amendments laid down by the World Medical Assemblies.

Before initiating the study in each country, approval of the corresponding regulatory authority and Institutional Review Board/ Independent Ethics Committee will be obtained.

15.1 Ethical consideration

15.1.1 Institutional Review Board/ Independent Ethics Committee (IRB / IEC)

The primary approval has been achieved, submission to other national and local IRB / IECs will be performed subsequently. The corresponding national PI together with CRI will provide substantial support for any IRB / IEC submission.

The sponsor is responsible to assure that approval of the local IRB / IEC in each country is obtained prior to study start in the respective study site or country in accordance with local requirements. A copy of the written and dated approval or favourable opinion of the local IRB / IEC signed by the chairman has to be filed with the study documents. The trial (study number, clinical trial protocol title and version number), the documents reviewed (clinical trial protocol, informed consent form, investigator’s CV, etc.) and the date of the review should be clearly stated on the written IRB / IEC approval/ favourable opinion.

During the trial, any substantial amendment or modification to the clinical trial protocol should be submitted to each responsible IRB / IEC. It should also be informed of any event likely to affect the safety of patients or the continued conduct of the trial, in particular of any change in safety.

If requested, a progress report is sent to the IRB / IEC annually and a summary of the trial's outcome at the end of the study.

15.1.2 Steering Committee

The Steering Committee (SC) will consist of a group of internationally recognized cardiologists (refer to Appendix III). The functions of the SC are the following:

- Advice on the scientific and clinical aspects of the study protocol and related documents.
- Scientific and clinical advice on the execution and scientific reporting of the study.
- Educated advice on the conduct of the study according to the guidelines of good clinical practice (GCP).
- Advice on the benefit / risk ratio following the recommendations of the Data and Safety Monitoring Board (DSMB).

15.1.3 Executive Steering Committee

The executive Steering Committee (eSC) will consist of a small group of expert cardiologists and an expert biostatistician (refer to Appendix IV). The functions of the eSC are the following:

- Overall responsibility for the execution and scientific reporting of EAST.
- Responsibility for the conduct of the study according to the guidelines of good clinical practice (GCP). This includes the monitoring of recruitment and measures to improve the distribution of therapies based on the recommendations of the DSMB.
- Reassessment of the sample size based on the blind review of the biostatistician.
- Reassessment of benefit/ risk ratio following the recommendations of the DSMB.
- Decisions on continuation or termination of the study based on the recommendations of the DSMB.

The eSC considers the advice of the SC in its decisions.

15.1.4 Endpoint Review Committee

The Endpoint Review Committee (ERC) will centrally adjudicate all outcome events in EAST, i. e.:

- cardiovascular death,
- TIA or stroke,
- worsening of heart failure assessed by hospitalisation,
- acute coronary syndrome assessed by hospitalisation and
- cardiovascular hospitalisation

as well as any hospitalisation for other reason and any other SAE. Furthermore, cardiovascular deaths will be sub-classified as AF-related death or non AF-related deaths.

The committee will be blinded to therapy group and will consist of experienced clinicians not related to the trial (refer to Appendix V).

15.1.5 Data and Safety Monitoring Board

The Data and Safety Monitoring Board (DSMB) is an independent group of experts that advises the SC and study investigators. It will consist of one statistician and two clinicians with expertise in clinical trials and in the management of AF patients (refer to Appendix VI). The members of the DSMB serve in an individual capacity and provide their expertise and recommendations. They regularly monitor the recruitment and conduct of trial, data quality and timeliness, the distribution of therapies within the study groups, the serious adverse events and further adverse events selected to their discretion during the course of the trial. They perform the open interim analyses of the group-sequential design and give recommendations to the eSC as to continue or stop the trial.

A DSMB charter providing operating procedures and responsibilities will be discussed and enacted latest at the second meeting. Meeting frequency will be defined by the committee and may vary depending on tasks. Meetings will be face-to-face or conference calls in exceptional cases and may have an open part with guests and a closed part. Minutes of each meeting will be provided. After each meeting, recommendations will be given to the eSC in a written form.

15.2 Legal Consideration

The study will be notified to the competent authority of each participating country and approval obtained prior to study start in the respective country. The study will be performed in accordance with the respective national legislation in each country.

15.3 Modification of Protocol

Any substantial amendment to the clinical trial protocol requires written approval/favourable opinion by the IRB /IEC prior to its implementation, unless there are overriding safety reasons that require immediate action. In some instances, an amendment may require a change to the informed consent form. In this case, the investigator must receive an IRB /IEC approval/favourable opinion concerning the revised informed consent form prior to implementation of the change.

Substantial amendments will be notified to the competent authorities too.

15.4 Financing and Insurance

The costs necessary to perform the study will be agreed upon with each investigator and will be documented in a separate financial agreement which will be signed by the investigator and the sponsor, prior to the study commencing.

A patient insurance has been effected by the sponsor of the trial.

Insurance carrier (German subsidiary):
Allianz Versicherungs AG
An den Treptowers 3, 12435 Berlin, Germany
Insurance policy number: AS-9000341825 (for German part)

Country specific requirements will be taken into account.

The insurance certificate as well as the insurance conditions will be handed out to all investigators. The insurance conditions have to be provided to the patients too.

15.5 Investigator's Information on Study Treatment

There is no stipulated drug therapy. Information on drugs used throughout the study is openly available.

15.6 Personal Data and Data Protection

All data obtained in the context of the clinical trial are subject to data protection. This applies to patients' data as well as to investigators' personal data which may be included in any database of the sponsor or CRI.

The investigating physicians shall take care that patient documents (e.g. copies of reports on special findings) transmitted to CRI or the sponsor contain no names, but only the year of birth and a relevant patient number. The storage of data for statistical analysis shall likewise be performed only under the patient’s random/study number.

15.7 Data Handling and Record Keeping

15.7.1 Completion of Case Report Forms

All medical data in this trial are to be recorded directly in the e-CRFs. Documentation on paper will be restricted to exceptional circumstances only.

The investigator must ensure the accuracy, completeness and timeliness (and legibility in case of documentation on paper) of data.

15.7.2 Archiving

The investigator must maintain confidential all study documentation, and take measures to prevent accidental or premature destruction of these documents. The investigator has to retain the study documents (i.e. investigator site file) at least five years after the completion or discontinuation of the study or longer if required by national legislation. This especially applies to patients' signed informed consent forms and the patient identification list.

The investigator must notify the sponsor prior to destroying any essential study documents within the specified period following completion or discontinuation of the trial.

15.8 Confidentiality

All information disclosed or provided by the sponsor (or any company/institution acting on his behalf), or produced during the trial, including, but not limited to, the clinical trial protocol, the e-CRFs and the results obtained during the course of the trial, is confidential. The investigator or any person under his/her authority agrees to undertake to keep confidential and not to disclose the information to any third party without the prior written approval of the sponsor. The sub-investigators shall be bound by the same obligation as the investigator. The investigator shall inform the sub-investigators of the confidential nature of the trial. Both, the investigator and the sub-investigators shall use the information solely for the purposes of the trial, to the exclusion of any use for their own or for a third party's account.

15.9 Responsibilities

The sponsor of this trial is responsible to Health Authorities for taking all reasonable steps to ensure the proper conduct of the trial with regard to ethical aspects, clinical trial protocol compliance, integrity and validity of the data recording.

16 Final Report and Publication Policy, Property Rights

The sponsor will be responsible for preparing the final study report that is to be signed by the eSC. The sponsor will communicate the results of the trial to the investigators.

The sponsor will be primarily responsible for the creation, review and submission of publications and presentations relating to the major aspects of the study within a timely fashion after completion of the study. All analyses will be the responsibility of the eSC. Manuscripts for publication will be drafted by members of the eSC or other interested investigators. All manuscripts will be subject to coordinated submission and review prior to submission. Coordination will be done by eSC.

EAST is an investigator-initiated trial. Interested investigators and initiatives will be encouraged and supported as appropriate if they propose additional issues that may be studied within the main trial. These materials must be submitted to the eSC for review and comment prior to publication or public dissemination. All relevant measures for transparency of clinical trials, and especially the recommendations of the editors of the major medical journals, will be met.

The publication rules are regulated separately and described in detail in a publication policy that is confirmed by the eSC and part of the contract of the SC-members.

All information and documents provided by the sponsor or its representatives are and remain the sole property of the sponsor. The investigator shall not mention any information for any other intellectual property rights.

All results, data, documents and inventions, which arise directly or indirectly from the trial in any form, shall be the immediate and exclusive property of the sponsor.

17 Definitions and Classifications

17.1 Protocol Violation

Protocol violations are any unapproved changes, deviations or departures from the study design or procedures of a research project that are under the investigator's control and that have not been reviewed and approved by the SC.

17.2 Major Protocol Violation

Major protocol violations are any unapproved changes in the research study design and/or procedures that are within the investigator's control and not in accordance with the IRB-approved protocol that may affect the participant's rights, safety or well-being, or the completeness, accuracy and reliability of the study data. Patients with major protocol violations will be excluded from the per protocol analysis. Study specific definitions of major protocol violations will be given by the SC.

17.3 Protocol Deviation

A failure to adhere to the pre-specified trial protocol, or a participant for whom this occurred. Examples are ineligible participants who were included in the trial by mistake and those for whom the intervention or other procedure differed from that outlined in the protocol.

18 References

1. Go AS, Hylek EM, Phillips KA, Chang Y, Henault LE, Selby JV, Singer DE. Prevalence of diagnosed atrial fibrillation in adults: national implications for rhythm management and stroke prevention: the AnTicoagulation and Risk Factors in Atrial Fibrillation (ATRIA) Study. *Jama.* 2001;285:2370-2375.

2. Nieuwlaat R, Capucci A, Camm AJ, Olsson SB, Andresen D, Davies DW, Cobbe S, Breithardt G, Le Heuzey JY, Prins MH, Levy S, Crijns HJ. Atrial fibrillation management: a prospective survey in ESC member countries: the Euro Heart Survey on Atrial Fibrillation. *Eur Heart J.* 2005;26:2422-2434.

3. Wolf PA, Abbott RD, Kannel WB. Atrial fibrillation as an independent risk factor for stroke: the Framingham Study. *Stroke.* 1991;22:983-988.

4. Lloyd-Jones DM, Wang TJ, Leip EP, Larson MG, Levy D, Vasan RS, D'Agostino RB, Massaro JM, Beiser A, Wolf PA, Benjamin EJ. Lifetime Risk for Development of Atrial Fibrillation: The Framingham Heart Study. *Circulation.* 2004;110:1042-1046.

5. Singh BN, Singh SN, Reda DJ, Tang XC, Lopez B, Harris CL, Fletcher RD, Sharma SC, Atwood JE, Jacobson AK, Lewis HD, Jr., Raisch DW, Ezekowitz MD. Amiodarone versus sotalol for atrial fibrillation. *N Engl J Med.* 2005;352:1861-1872.

6. Fuster V, Ryden LE, Cannom DS, Crijns HJ, Curtis AB, Ellenbogen KA, Halperin JL, Le Heuzey JY, Kay GN, Lowe JE, Olsson SB, Prystowsky EN, Tamargo JL, Wann S, Priori SG, Blanc JJ, Budaj A, Camm AJ, Dean V, Deckers JW, Despres C, Dickstein K, Lekakis J, McGregor K, Metra M, Morais J, Osterspey A, Tamargo JL, Zamorano JL, Smith SC, Jr., Jacobs AK, Adams CD, Anderson JL, Antman EM, Halperin JL, Hunt SA, Nishimura R, Ornato JP, Page RL, Riegel B. ACC/AHA/ESC 2006 guidelines for the management of patients with atrial fibrillation-executive summary: A report of the American College of Cardiology/American Heart Association Task Force on practice guidelines and the European Society of Cardiology Committee for Practice Guidelines (Writing Committee to Revise the 2001 Guidelines for the Management of Patients with Atrial Fibrillation) Developed in collaboration with the European Heart Rhythm Association and the Heart Rhythm Society. *Eur Heart J.* 2006;27:1979-2030.

7. Singh BN, Connolly SJ, Crijns HJ, Roy D, Kowey PR, Capucci A, Radzik D, Aliot EM, Hohnloser SH. Dronedarone for maintenance of sinus rhythm in atrial fibrillation or flutter. *N Engl J Med.* 2007;357:987-999.

8. Calkins H, Brugada J, Packer DL, Cappato R, Chen SA, Crijns HJ, Damiano RJ, Jr., Davies DW, Haines DE, Haissaguerre M, Iesaka Y, Jackman W, Jais P, Kottkamp H, Kuck KH, Lindsay BD, Marchlinski FE, McCarthy PM, Mont JL, Morady F, Nademanee K, Natale A, Pappone C, Prystowsky E, Raviele A, Ruskin JN, Shemin RJ. HRS/EHRA/ECAS expert Consensus Statement on catheter and surgical ablation of atrial fibrillation: recommendations for personnel, policy, procedures and follow-up. A report of the Heart Rhythm Society (HRS) Task Force on catheter and surgical ablation of atrial fibrillation. *Heart Rhythm.* 2007;4:816-861.

9. Haissaguerre M, Jais P, Shah DC, Takahashi A, Hocini M, Quiniou G, Garrigue S, LeMouroux A, LeMetayer P, Clementy J. Spontaneous initiation of atrial fibrillation by ectopic beats originating in the pulmonary veins. *N Engl J Med.* 1998;339:659-666.

10. Marini C, De Santis F, Sacco S, Russo T, Olivieri L, Totaro R, Carolei A. Contribution of atrial fibrillation to incidence and outcome of ischemic stroke: results from a population-based study. *Stroke.* 2005;36:1115-1119.

11. Kirchhof P, Auricchio A, Bax J, Crijns H, Camm J, Diener HC, Goette A, Hindricks G, Hohnloser S, Kappenberger L, Kuck KH, Lip GY, Olsson B, Meinertz T, Priori S, Ravens U, Steinbeck G, Svernhage E, Tijssen J, Vincent A, Breithardt G. Outcome parameters for trials in atrial fibrillation: executive summary: Recommendations from a consensus conference organized by the German Atrial Fibrillation Competence NETwork (AFNET) and the European Heart Rhythm Association (EHRA). *Eur Heart J.* 2007;28:2803-2817.

12. AFFIRM I. A comparison of rate control and rhythm control in patients with atrial fibrillation. *N Engl J Med.* 2002;347:1825-1833.

13. Van Gelder I, Hagens VE, Bosker HA, Kingma H, Kamp O, Kingma T, Said SA, Darmanata JI, Timmermanns AJM, Tijssen JGP, Crijns HJ. A comparison of rate control and rhythm control in patients with recurrent persistent atrial fibrillation. *N Engl J Med.* 2002;347:1834-1840.

14. Carlsson J, Miketic S, Windeler J, Cuneo A, Haun S, Micus S, Walter S, Tebbe U, Investigators S. Randomzied trial of rate-control versus rhythm-control in persistent atrial fibrillation. *J Am Coll Cardiol.* 2003;41:1690-1696.

15. Opolski G, Torbicki A, Kosior DA, Szulc M, Wozakowska-Kaplon B, Kolodziej P, Achremczyk P. Rate control vs rhythm control in patients with nonvalvular persistent atrial fibrillation: the results of the Polish How to Treat Chronic Atrial Fibrillation (HOT CAFE) Study. *Chest.* 2004;126:476-486.

16. Hohnloser SH, Kuck KH, Lilienthal J. Rhythm or rate control in atrial fibrillation--Pharmacological Intervention in Atrial Fibrillation (PIAF): a randomised trial. *Lancet.* 2000;356:1789-1794.

17. Roy D, Talajic M, Nattel S, Wyse DG, Dorian P, Lee KL, Bourassa MG, Arnold JM, Buxton AE, Camm AJ, Connolly SJ, Dubuc M, Ducharme A, Guerra PG, Hohnloser SH, Lambert J, Le Heuzey JY, O'Hara G, Pedersen OD, Rouleau JL, Singh BN, Stevenson LW, Stevenson WG, Thibault B, Waldo AL. Rhythm control versus rate control for atrial fibrillation and heart failure. *N Engl J Med.* 2008;358:2667-2677.

18. Corley SD, Epstein AE, DiMarco JP, Domanski MJ, Geller N, Greene HL, Josephson RA, Kellen JC, Klein RC, Krahn AD, Mickel M, Mitchell LB, Nelson JD, Rosenberg Y, Schron E, Shemanski L, Waldo AL, Wyse DG. Relationships between sinus rhythm, treatment, and survival in the Atrial Fibrillation Follow-Up Investigation of Rhythm Management (AFFIRM) Study. *Circulation.* 2004;109:1509-1513.

19. Hohnloser SH, Crijns HJ, van Eickels M, Gaudin C, Page RL, Torp-Pedersen C, Connolly SJ. Effect of dronedarone on cardiovascular events in atrial fibrillation. *N Engl J Med.* 2009;360:668-678.

20. Testa L, Biondi-Zoccai GG, Dello Russo A, Bellocci F, Andreotti F, Crea F. Rate-control vs. rhythm-control in patients with atrial fibrillation: a meta-analysis. *Eur Heart J.* 2005;26:2000-2006.

21. Nabauer M, Gerth A, Limbourg T, Schneider S, Oeff M, Kirchhof P, Goette A, Lewalter T, Ravens U, Meinertz T, G. B, Steinbeck G. The Registry of the German Competence NETwork

on Atrial Fibrillation: Patient characteristics and initial management. *Europace.* 2008;under revision.

22. Kober L, Torp-Pedersen C, McMurray JJ, Gotzsche O, Levy S, Crijns H, Amlie J, Carlsen J. Increased mortality after dronedarone therapy for severe heart failure. *N Engl J Med.* 2008;358:2678-2687.

23. Koebe J, Kirchhof P. Novel non-pharmacological approaches for antiarrhythmic therapy of atrial fibrillation. *Europace.* 2008;10:433-437.

24. Allessie M, Ausma J, Schotten U. Electrical, contractile and structural remodeling during atrial fibrillation. *Cardiovasc Res.* 2002;54:230-246.

25. Kirchhof P, Bax J, Blomstrom-Lundquist C, Calkins H, John Camm A, Cappato R, Cosio F, Crijns H, Diener HC, Goette A, Israel CW, Kuck KH, Lip GY, Nattel S, Page RL, Ravens U, Schotten U, Steinbeck G, Vardas P, Waldo A, Wegscheider K, Willems S, Breithardt G. Early and comprehensive management of atrial fibrillation: executive summary of the proceedings from the 2nd AFNET-EHRA consensus conference 'research perspectives in AF'. *Eur Heart J.* 2009.

26. Cosio FG, Aliot E, Botto GL, Heidbuchel H, Geller CJ, Kirchhof P, De Haro JC, Frank R, Villacastin JP, Vijgen J, Crijns H. Delayed rhythm control of atrial fibrillation may be a cause of failure to prevent recurrences: reasons for change to active antiarrhythmic treatment at the time of the first detected episode. *Europace.* 2008;10:21-27.

27. Kirchhof P, Auricchio A, Bax J, Crijns H, Camm J, Diener HC, Goette A, Hindricks G, Hohnloser S, Kappenberger L, Kuck KH, Lip GY, Olsson B, Meinertz T, Priori S, Ravens U, Steinbeck G, Svernhage E, Tijssen J, Vincent A, Breithardt G. Outcome parameters for trials in atrial fibrillation: Recommendations from a consensus conference organized by the German Atrial Fibrillation Competence NETwork and the European Heart Rhythm Association. *Europace.* 2007;9:1006-1023.

28. Thygesen K, Alpert JS, White HD, Jaffe AS, Apple FS, Galvani M, Katus HA, Newby LK, Ravkilde J, Chaitman B, Clemmensen PM, Dellborg M, Hod H, Porela P, Underwood R, Bax JJ, Beller GA, Bonow R, Van Der Wall EE, Bassand JP, Wijns W, Ferguson TB, Steg PG, Uretsky BF, Williams DO, Armstrong PW, Antman EM, Fox KA, Hamm CW, Ohman EM, Simoons ML, Poole-Wilson PA, Gurfinkel EP, Lopez-Sendon JL, Pais P, Mendis S, Zhu JR, Wallentin LC, Fernandez-Aviles F, Fox KM, Parkhomenko AN, Priori SG, Tendera M, Voipio-Pulkki LM, Vahanian A, Camm AJ, De Caterina R, Dean V, Dickstein K, Filippatos G, Funck-Brentano C, Hellemans I, Kristensen SD, McGregor K, Sechtem U, Silber S, Tendera M, Widimsky P, Zamorano JL, Morais J, Brener S, Harrington R, Morrow D, Sechtem U, Lim M, Martinez-Rios MA, Steinhubl S, Levine GN, Gibler WB, Goff D, Tubaro M, Dudek D, Al-Attar N. Universal definition of myocardial infarction: Kristian Thygesen, Joseph S. Alpert and Harvey D. White on behalf of the Joint ESC/ACCF/AHA/WHF Task Force for the Redefinition of Myocardial Infarction. *Eur Heart J.* 2007;28:2525-2538.

29. Schulman S, Kearon C. Definition of major bleeding in clinical investigations of antihemostatic medicinal products in non-surgical patients. *J Thromb Haemost.* 2005;3:692-694.

30. Wang J, Ruotsalainen S, Moilanen L, Lepisto P, Laakso M, Kuusisto J. The metabolic syndrome predicts incident stroke: a 14-year follow-up study in elderly people in Finland. *Stroke.* 2008;39:1078-1083.

31. Connolly SJ. Evidence-based analysis of amiodarone efficacy and safety. *Circulation.* 1999;100:2025-2034.

32. Jabaudon D, Sztajzel J, Sievert, Landis T, Sztajzel R. Usefulness of Ambulatory 7-Day ECG Monitoring for the Detection of Atrial Fibrillation and Flutter After Acute Stroke and Transient Ischemic Attack. *Stroke.* 2004.

33. Liao J, Khalid Z, Scallan C, Morillo C, O'Donnell M. Noninvasive cardiac monitoring for detecting paroxysmal atrial fibrillation or flutter after acute ischemic stroke: a systematic review. *Stroke.* 2007;38:2935-2940.

34. Albers GW. Stroke prevention in atrial fibrillation: pooled analysis of SPORTIF III and V trials. *Am J Manag Care.* 2004;10:S462-469; discussion S469-473.

35. Bousser MG, Bouthier J, Buller HR, Cohen AT, Crijns H, Davidson BL, Halperin J, Hankey G, Levy S, Pengo V, Prandoni P, Prins MH, Tomkowski W, Thorp-Pedersen C, Wyse DG. Comparison of idraparinux with vitamin K antagonists for prevention of thromboembolism in patients with atrial fibrillation: a randomised, open-label, non-inferiority trial. *Lancet.* 2008;371:315-321.

36. Connolly SJ, Crijns HJ, Torp-Pedersen C, van Eickels M, Gaudin C, Page RL, Hohnloser SH. Analysis of stroke in ATHENA: a placebo-controlled, double-blind, parallel-arm trial to assess the efficacy of dronedarone 400 mg BID for the prevention of cardiovascular hospitalization or death from any cause in patients with atrial fibrillation/atrial flutter. Circulation 2009;120:1174-80

37. Wilber DJ, Pappone C, Neuzil P, De Paola A, Marchlinski F, Natale A, Macle L, Daoud EG, Calkins H, Hall B, Reddy V, Augello G, Reynolds MR, Vinekar C, Liu CY, Berry SM, Berry DA. Comparison of antiarrhythmic drug therapy and radiofrequency catheter ablation in patients with paroxysmal atrial fibrillation: a randomized controlled trial. JAMA;303:333-40.

38. Van Gelder IC, Groenveld HF, Crijns HJGM, Tuininga YS, Tijssen JGP, Alings AM, Hillege HL, Bergsma-Kadijk JA, Cornel JH, Kamp O, Tukkie R, Bosker HA, Van Veldhuisen DJ, Van den Berg MP. Lenient versus Strict Rate Control in Patients with Atrial Fibrillation. N Engl J Med; published at www.nejm.org on March 15, 2010 (10.1056/NEJMoa1001337)

39. Dorian P, Guerra PG, Kerr CR, O'Donnell SS, Crystal E, Gillis AM, Mitchell LB, Roy D, Skanes AC, Rose MS, Wyse DG. Validation of a new simple scale to measure symptoms in atrial fibrillation: the Canadian Cardiovascular Society Severity in Atrial Fibrillation scale. Circ Arrhythm Electrophysiol 2009;2:218-24.

40. Camm AJ, Kirchhof P, Lip GY, Schotten U, Savelieva I, Ernst S, Van Gelder IC, Al-Attar N, Hindricks G, Prendergast B, Heidbuchel H, Alfieri O, Angelini A, Atar D, Colonna P, De Caterina R, De Sutter J, Goette A, Gorenek B, Heldal M, Hohloser SH, Kolh P, Le Heuzey JY, Ponikowski P, Rutten FH, Vahanian A, Auricchio A, Bax J, Ceconi C, Dean V, Filippatos G, Funck-Brentano C, Hobbs R, Kearney P, McDonagh T, Popescu BA, Reiner Z, Sechtem U, Sirnes PA, Tendera M, Vardas PE, Widimsky P, Agladze V, Aliot E, Balabanski T, Blomstrom-Lundqvist C, Capucci A, Crijns H, Dahlof B, Folliguet T, Glikson M, Goethals M, Gulba DC, Ho SY, Klautz RJ, Kose S, McMurray J, Perrone Filardi P, Raatikainen P, Salvador MJ, Schalij MJ, Shpektor A, Sousa J, Stepinska J, Uuetoa H, Zamorano JL, Zupan I. Guidelines for the management of atrial fibrillation: The Task Force for the Management of Atrial Fibrillation of

the European Society of Cardiology (ESC). Eur Heart J. 2010 Aug 29; published on line Aug 29, 1010 (doi: ehq278 [pii] 10.1093/eurheartj/ehq278).

41. Van Gelder IC, Groenveld HF, Crijns HJ, Tuininga YS, Tijssen JG, Alings AM, Hillege HL, Bergsma-Kadijk JA, Cornel JH, Kamp O, Tukkie R, Bosker HA, Van Veldhuisen DJ, Van den Berg MP. Lenient versus strict rate control in patients with atrial fibrillation. N Engl J Med 2010;362:1363-73

42. Glotzer TV, Daoud EG, Wyse DG, Singer DE, Ezekowitz MD, Hilker C, Miller C, Qi D, Ziegler PD. The relationship between daily atrial tachyarrhythmia burden from implantable device diagnostics and stroke risk: the TRENDS study. Circ Arrhythm Electrophysiol 2009;2:474-80.

43. Kaleschke G, Hoffmann B, Drewitz I, Steinbeck G, Naebauer M, Goette A, Breithardt G, Kirchhof P. Prospective, multicentre validation of a simple, patient-operated electrocardiographic system for the detection of arrhythmias and electrocardiographic changes. Europace 2009;11:1362-8.

19 Signatures

The undersigned have read this protocol and agreed to conduct this study in accordance with all stipulations of the protocol and in accordance with the Declaration of Helsinki.

Date	Signature
____________________	____________________<br>Prof. Paulus Kirchhof<br>International Chief Investigator
____________________	____________________<br>Prof. Günter Breithardt<br>Co-ordinating investigator
____________________	____________________<br>Prof. Harry Crijns<br>Co-ordinating investigator
____________________	____________________<br>Prof. John Camm<br>Co-ordinating investigator
____________________	____________________<br>Prof. Andreas Götte<br>Sponsor representative
____________________	____________________<br>Prof. Karl-Heinz Kuck<br>Member of the executive Steering Committee
____________________	____________________<br>Prof. Panos Vardas<br>Member of the executive Steering Committee
____________________	____________________<br>Prof. Karl Wegscheider<br>Study statistician
____________________	____________________<br>Signature local investigator
	____________________<br>(Name local investigator; printed)

20 Appendices

Appendix I: List of participating countries / national principal investigators

(sorted in alphabetical order)

Country	Name of national principal investigator
France	Prof. Etienne Aliot, Nancy
Denmark	Prof. Axel Brandes, Odense
Netherlands	Prof. Arif Elvan, Zwolle<br>Prof. Isabelle van Gelder, Groningen
Italy	Prof. Michele Gulizia, Catania<br>Prof. Sakis Themistoclakis, Venice
Switzerland	Dr. Laurent Haegeli, Zurich
Belgium	Prof. Hein Heidbuchel, Leuven
Czech Republic	Prof. Josef Kautzner, Prague
Spain	Prof. Lluis Mont, Barcelona
Great Britain	Dr. Andre Ng, Leicester
Poland	Prof. Lukasz Szumowski, Warsaw
Germany	Prof. Lars Eckardt, Münster<br>Prof. Stephan Willems, Hamburg

Appendix II: List of participating study sites

(separate document)

Appendix III: Members of the Steering Committee

(sorted in alphabetical order)

Prof. Etienne Aliot	CHU de Nancy, Rue du Morvan<br>54500 Vandoeuvre-lès-Nancy, France
Prof. Axel Brandes	Odense University Hospital, Sdr. Boulevard 29<br>5000 Odense C, Denmark
Prof. Dr. Günter Breithardt	Mendelstraße 11<br>48149 Münster, Germany
Prof. Dr. John Camm	St. George's Hospital Medical School, Cranmer Terrace<br>SW 17 ORE London, Great Britain
Prof. Dr. Harry Crijns	University Hospital Maastricht, P. Debyelaan 26<br>6202 AZ Maastricht, Netherlands
Prof. Dr. Lars Eckardt	Universitätsklinikum Münster, Albert-Schweitzer-Str. 33
Prof. Arif Elvan	Isala Klinieken, Postbus 10500<br>8000 GM Zwolle, Netherlands
Dr. Thomas Fetsch and<br>responsible project manager<br>(non-voting members)	CRI, Arnulfstr. 19,<br>80335 München, Germany
Prof. Dr. Isabelle C. Van Gelder	University Medical Center Groningen, Postbus 30001<br>9700RB Groningen, Netherlands
Prof. Dr. Andreas Götte<br>(Sponsor representative)	St. Vincenz Krankenhaus, Am Busdorf 2<br>33098 Paderborn, Germany
Prof. Dr. Michele Gulizia	Garibaldi-Nesima Hospital, Via Palermo 636<br>95122 Catania, Italy
Dr. Laurent Haegeli	Universitätsspital Zürich, Rämistrs.<br>8091 Zürich, Switzerland
Prof. Dr. Hein Heidbuchel	UZ Leuven, Herestraat 49<br>3000 Leuven, Belgium
Prof. Dr. Josef Kautzner	Institute for Clinical and Experimental Medicine (IKEM),<br>Videnska 1958/9<br>14021 Prague 4, Czech Republic
Prof. Dr. Paulus Kirchhof<br>(chair)	University of Birmingham Centre for Cardiovascular Sciences,<br>Vincent Drive<br>Birmingham B15 2TT, England, UK
Prof. Dr. Karl-Heinz Kuck	Mendelstraße 11<br>48149 Münster, Germany

Representative of Sanofi<br>(non-voting member)	Sanofi, France
Representative of St. Jude Medical<br>(non-voting member)	St. Jude Medical, USA
Prof. Dr. Lluis Mont	Hospital Clinic Barcelona, c/Villarroel no. 170, Esc. 1, 6° Planta<br>8036 Barcelona, Spain
Dr. André Ng	Glenfield General Hospital, Clinical Sciences Wing<br>Leicester, LE3 9QP, Great Britain
Prof. Dr. Lukasz Szumowski	National Institute of Cardiology, Alpejska 42<br>04-620 Warsaw, Poland
Prof. Sakis Themistoclakis	Ospedale del 'Angelo, Via Paccagnella 11<br>30174 Mestre, Italy
Prof. Dr. Panos Vardas	Heraklion University, Department of Cardiology, PO Box 1352<br>Stavrakia<br>711 10 Heraklion, Greece
Prof. Dr. Karl Wegscheider	Institut für Med. Biometrie und Epidemiologie, Martinistrasse 52<br>20246 Hamburg, Germany
Prof. Dr. Stephan Willems	Asklepios Klinik St. Georg, Lohmühlenstr.5, 2009 Hamburg,<br>Germany

Appendix IV: Members of the Executive Steering Committee

(sorted in alphabetical order)

Prof. Dr. Günter Breithardt	Mendelstraße 11<br>48149 Münster, Germany
Prof. Dr. John Camm	St. George's Hospital Medical School, Cranmer Terrace<br>SW 17 ORE London, Great Britain
Prof. Dr. Harry Crijns	University Hospital Maastricht, P. Debyelaan 26<br>6202 AZ Maastricht, Netherlands
Prof. Dr. Andreas Götte<br>(sponsor representative)	St. Vincenz Krankenhaus, Am Busdorf 2<br>33098 Paderborn, Germany
Prof. Dr. Paulus Kirchhof<br>(chair)	University of Birmingham Centre for Cardiovascular Sciences, Vincent Drive<br>Birmingham B15 2TT, England, UK
Prof. Dr. Karl-Heinz Kuck	Mendelstraße 11<br>48149 Münster, Germany
Representative of St. Jude Medical<br>(non-voting member)	St. Jude Medical, USA
Representative of Sanofi<br>(non-voting member)	Sanofi, France
Prof. Dr. Panos Vardas	Heraklion University, Department of Cardiology, PO Box 1352 Stavrakia<br>711 10 Heraklion, Greece
Prof. Dr. Karl Wegscheider	Institut für Med. Biometrie und Epidemiologie, Martinistrasse 52<br>20246 Hamburg, Germany

Appendix V: Members of the Endpoint Review Committee

(sorted in alphabetical order)

Prof. Hans-Christoph Diener	University Duisburg-Essen, Department of Neurology,<br>Hufelandstr 55<br>45122 Essen, Germany
Prof. Dr. Laurent Fauchier	Centre Hospitalier Universitaire Trousseau, Cardiology,<br>Avenue de la République<br>37004 Tours, France
Prof. em. Dr. Helmut Klein (chair)	Waltherstrasse 9c,<br>80337 Munich, Germany
Dr. Francisco Marin	University Hospital Virgen de la Arrixaca, Cardiology,<br>Ctra. Madrid-Cartagena, s/n<br>30120 El Palmar, Murcia, Spain

Appendix VI: Members of the Data and Safety Monitoring Board

(sorted in alphabetical order)

Prof. Dr. Marie-Germaine Bousser	Hôpital Lariboisière, Service de Neurologie, 2, rue Ambroise Paré<br>75475 Paris cédex 10, France
Prof. Dr. Ian Ford	Robertson Centre for Biostatistics,<br>Level II, Boyd Orr Building, University Avenue<br>University of Glasgow<br>Glasgow, G12 8 QQ, Great Britain
Prof. Dr. Thomas Meinertz (chair)	Universitäres Herzzentrum, Abteilung für Kardiologie III, Martinistraße 52<br>20246 Hamburg, Germany

Appendix VII: (deleted with protocol amendment 14.05.2015)

Appendix VIII: Patient Information and Informed Consent

(Note: The German version of the patient information and informed consent will be used as basis for translation into local languages. The following English translation does not correspond to the English version to be used in UK study sites but only as an English master version)

Dear patient,

Your doctor has invited you to participate in the clinical trial described below (EAST study).

Clinical trials are needed to gain insight into or expand our knowledge of the efficacy and tolerability of medicines and medicinal products and are intended to help to improve the current treatment. This clinical trial has been approved by the competent Ethics Committee and the competent authority as required by legislation. The EAST study will be conducted in 11 European countries and altogether approximately 200 hospitals and medical practices will be involved; about 3000 patients will participate in this trial. This clinical trial will be conducted jointly by the European Heart Rhythm Association (EHRA; www.escardio.org/communities/EHRA) and the German Competence Network on Atrial Fibrillation (AFNET; www.kompetenznetz-vorhofflimmern.de), a research network funded by the Federal Ministry of Education and Research (BMBF; www.bmbf.de) which links German hospitals and medical practices specialised in cardiology nationwide. The AFNET funds and is responsible for this study (sponsor).

Your participation in this clinical trial is voluntary. Thus you will be involved in this clinical trial only after having consented in writing. If you do not want to participate or would like to discontinue your participation, this decision will in no way unfavourably influence your further treatment.

You have already been approached regarding the planned clinical trial. In the following, the study objectives and procedures will be explained to you. Subsequently, a study doctor will conduct the informed consent discussion with you. Please do not hesitate to address any issues that you do not fully understand. You will then be given enough time to decide whether or not you wish to participate in this study.

1. Why is this clinical trial **be** performed?

You have atrial fibrillation, a common type of cardiac rhythm disorder, which unluckily represents an important cause for stroke but also for myocardial infarction and heart muscle weakness (heart failure). These serious consequences of atrial fibrillation can unluckily not be prevented completely by currently available methods and techniques.

Atrial fibrillation is present when the heart's upper chambers (the atria) beat very rapidly and irregularly, resulting in an irregular pulse. In some patients, atrial fibrillation is only present for certain periods of time or these patients may experiences attacks of atrial fibrillation, while other patients may suffer from continuous atrial fibrillation. Many patients report symptoms such as irregular or racing heartbeat, shortness of breath or anxiety. However, the atrial fibrillation may often go completely unnoticed. Although atrial fibrillation is not a directly life-threatening condition in itself, it may have dangerous consequences. For example, the risk of stroke is up to five times higher in patients with atrial fibrillation. Patients with additional cardiovascular risk factors (e.g. high blood pressure, diabetes, older than 65 years of age) are at an even higher risk of suffering a stroke. Despite advances in the treatment of atrial fibrillation, we can still not prevent all of these severe complications.

In the EAST study we hope to demonstrate that an early as possible treatment of atrial fibrillation can prevent a stroke, heart muscle weakness (heart failure) and myocardial infarctions.

2. Will I receive the study drug in any case?

The EAST study does not involve the use of new or investigational medicines. All methods of treatment chosen for this study are approved by the competent health authorities and recommended for the treatment of atrial fibrillation. .In the trial, we will rather compare usual management of atrial fibrillation to early therapy that begins directly after the initial diagnosis of AF.

In case you decide to participate in this study, you will be assigned by chance to one of the two treatment groups (this procedure is called randomisation). Patients will be assigned in a 1:1 ratio and this approach will not be changed during the entire study. One group of patients will be treated according to the current guidelines of the European Society of Cardiology (the “Usual Care“ group). The other patient group will also be treated according to the guidelines, but will receive a therapy for prevention of atrial fibrillation earlier than usual - by means of catheter ablation and/or pharmacotherapy, depending on your doctor's decision (“Early Therapy” group).

3. What are the procedures of this clinical trial and what do I have to bear in mind in case of participation?

During the initial examination your doctor will take your medical history and perform a comprehensive physical examination. This will include measurement of blood pressure, recording of an ECG at rest, blood collection (15 ml) and an echocardiogram (ultrasound examination of the heart). In an interview, your doctor will do the so-called MoCA test (“Montreal Cognitive Assessment“) with you to determine your abilities for attention, concentration and memory. In addition you will be asked to rate your current state of health using a scale from 0 (“extremely poor“) to 100 (“perfect“). You will also be asked to complete two questionnaires which are used to determine the potential impact of your heart disorder on your quality of life. Altogether, it will take about 10 minutes to answer these questions.

If you agree, further 20 ml of blood will be collected at start of the study for scientific analyses. The purpose of these blood analyses is to gain new insights into how atrial fibrillation developes. These analyses will form a basis to further improve treatment in the future. These analyses will be performed in a central laboratory to discover new causes of atrial fibrillation. Your blood samples will be analysed “pseudonymously”, i.e. not linked to you as a person. We will ask you to provide us with a separate consent for these analyses.

Except for the “MoCA test“ and the quality of life questionnaires, these examinations would also be performed if you were not a participant in this clinical trial, but were only treated for problems with your heart rhythm.

Further study participation will depend on the result of this baseline examination. In case of participation two follow-up visits will take place at annual intervals (months 12 and 24) in the hospital/medical practice of your study doctor. In between these visits, you will receive, likewise at annual intervals (months 6, 18, 30) and thereafter in 6-monthly intervals (months 36, 42, 48, 54, etc.), a questionnaire by mail to collect information about hospital stays and other important events during the past year respectively 6-month-period. The number of questionnaires will depend on the total duration of this clinical trial: in any case you will regularly be followed-up until the end of the entire study, with a minimum follow-up period of two and a half years.

Depending on the treatment group you are assigned to, your study doctor will determine your further treatment. If your atrial fibrillation is continuously present, a procedure called “electrical cardioversion” may be performed as soon as possible. This procedure involves that the heart is set back into the normal rhythm using a defibrillator (cardioverter) which delivers targeted electric shocks (from outside to the chest). This treatment is performed under anaesthesia, and almost always the normal heart rhythm is restored. If you have been assigned to the “Early Therapy“ group, it is possible that an ablation procedure is performed during a cardiac catheterisation. This intervention is also intended to stop your atrial fibrillation. Both therapies are standard treatments of atrial fibrillation. In addition,

medications ("antiarrhythmic drugs") may be given to you to prevent atrial fibrillation. If the study doctor who is treating you does not perform catheter ablations, he/she will refer you to a cardiac ablation facility taking part in this study where you receive this treatment. Regardless of the treatment group you will be in, you will always receive the drug or interventional treatment that is best for you. Your study doctor will choose this therapy for you.

If you are randomized to the “Early Therapy” group, you will be handed out a small, patient-operated ECG device of similar size as a cheque card (as a loan). Your study doctor will explain to you in detail the handling of this device. You will be asked to record your ECG daily (at least 2-3 times a week or more frequently in case you experience any symptoms such as irregular and racing heartbeat). You can transmit these ECG recordings by telephone free of charge and at any time to a central evaluation office. In case atrial fibrillation is detected in an ECG, your study doctor will ask you to visit his/her hospital or local practice for a control examination where an ECG at rest will be performed in order to confirm atrial fibrillation, if any.

At the annual follow-up visits, the same procedures as during the baseline examination will be performed: measurement of blood pressure, ECG at rest and blood collection (15 ml). At the month 24-visit, an echocardiogram (ultrasound examination of the heart) and the so-called MoCA test will be performed again. In addition you will be asked again to rate your current state of health using a scale from 0 (“extremely poor“) to 100 (“perfect“) and to complete the two questionnaires you already know from the baseline examination.

IMPORTANT: Please keep your study doctor informed about any hospital stays as well as any medical treatment you may receive from another doctor during this clinical trial. You will receive a so-called “alert card“ on which your subject number is stated. You should always carry it with you as it would also be useful in case there should be any queries.

4. What is my potential benefit when participating in this clinical trial?

Apart from a thorough examination, you will probably receive no other personal health benefits from participating in this clinical trial. However, the results of this clinical trial shall help to improve the treatment of patients with atrial fibrillation in the future.

5. Which risks and inconveniences are associated with study participation?

With your participation in this clinical trial, no risks are involved other than those typically associated with the standard treatment for atrial fibrillation. For example, even a simple procedure such as e.g. the collection of blood from a vein, may lead to adverse consequences, such as a haematoma at the puncture site or an infection.

Your study doctor will discuss with you in detail the risks associated with cardioversion and catheter ablation as well as the potential side effects of the medication you are taking to treat your atrial fibrillation, once he/she has decided on a specific therapy for you.

Please inform your study doctor or his/her staff about all symptoms, illnesses, or injuries that you may experience during this clinical trial. In case these are serious, please immediately notify your study doctor or his/her staff, if applicable by phone.

6. Are there alternative treatment options beyond this clinical trial?

If you do not wish to participate in this clinical trial, essentially the same treatment options are available to you. Of course, you can also decide against any form of treatment. However, you should be aware that you may continue to experience atrial fibrillation and that the risk of cardiovascular complications, in particular stroke, may increase.

7. Who is not allowed to participate in this clinical trial?

You are not eligible to participate in this clinical trial, if you are at the same time taking part in other clinical trials or research projects or have taken part in any clinical trial or research project within the last two months.

Pregnant and breastfeeding women are **not eligible** to participate in this clinical trial.

Only applicable to women of childbearing potential: If you participate in this clinical trial, you will have to use reliable methods of contraception. These include oral contraceptives ("the pill") and intrauterine devices (IUDs). Should you become pregnant or suspect that you may have become pregnant during the clinical trial, you have to inform your study doctor immediately.

8. Will costs result from study participation? Will I be reimbursed for study participation?

You will not face any extra costs on top of the costs of your regular treatment because of your participation in this clinical trial.

9. Will I get an insurance during study participation?

In case of a study-related damage to your health, you will be covered by an insurance policy to the extent specified in the insurances terms and conditions. The policy covers damages up to a maximum sum insured of EUR 500,000. This insurance will be taken out because of a legal obligation for studies in general to provide such insurance cover and not because any damage to your health would be expected. This insurance policy does not cover damages only indirectly related to this clinical trial, such as e.g. accidents while travelling to the study site.

In order not to jeopardise your insurance cover, you are obliged to obey to the following rules:

a) During the duration of the clinical trial, you may only undergo another medical treatment except in cases of emergency – after consultation with your study doctor. You are obliged to inform your study doctor immediately about any emergency treatment you may receive.

b) If you suspect that as the result of your participation in the clinical trial a damage to your health has occurred or an existing condition has deteriorated, you have to inform your insurer immediately about it, e.g. with the support of your study doctor:

**Name and address of the insurance company:**	Allianz Versicherungs AG<br>An den Treptowers 3<br>12435 Berlin, Germany
Telephone:	00800 / 11224444
Insurance policy number:	AS-9000341825 (for German part)

You will receive a copy of the insurance terms and conditions on request from your study doctor.

10. Will I be informed about new findings during this clinical trial?

You will be informed about new information related to this clinical trial that may arise and that may influence your willingness to continue to participate. Based on this information, you can then decide whether you wish to continue to take part in this clinical trial.

11. Who decides whether I will be excluded from this clinical trial?

You are free to withdraw your consent at any time and without giving any reason. If you do not wish to continue to participate in this study, you will not experience any disadvantages in your medical treatment as the result of this decision. If you decide to discontinue study participation, you will be asked to confirm your decision to withdraw your consent to participate in writing.

In certain circumstances, your participation in this clinical trial may be terminated early by the sponsor / executive board or the national or foreign regulatory authorities; you will have no influence on such decisions. A reason for this may be that e.g. the entire clinical trial is terminated early. In such an event, your study doctor will discuss with you your further treatment.

Should you decide to withdraw from this clinical trial early, or to terminate your participation early for any other reason, your study doctor will ask you to agree to be contacted once again at the end of the entire clinical trial to gather information about your state of health.

Your study doctor will discuss with you details regarding your further treatment.

12. What happens with my data?

During a clinical trial, the treating doctors are obliged to document the course of the treatment and special events, such as hospital stays, in documentation sheets specifically designed for this purpose.

To ensure that all data were correctly transferred from your medical files to the documentation sheets, entries in these documentation sheets may be compared with the original data by members of staff of the competent regulatory authority or federal health authorities (European Medicines Evaluation Agency, as well as other foreign regulatory authorities) and by specially trained members of staff which are bound to confidentiality, so-called “monitors“ or “auditors“. In addition, an authorised representative of a national or foreign regulatory authority or a monitor is permitted to copy pseudonymised documents. Pseudonymised means that no personal data, e.g. names are used, but only a number code with the year of birth. Any information is treated strictly confidential. Your name and other information that may be used to identify you will not appear in these copies and in scientific publications that may result from this clinical trial.

Data relevant to this clinical trial are centrally stored in pseudonymised form at the Munich-based Clinical Research Institute (CRI) and, if required, forwarded to the above mentioned authorities for verification. The CRI will be responsible for data processing of this clinical trial. Statistical analyses will be performed with anonymous data (i.e. only number code, without reference to your study doctor) by the institute for medical biometrics and epidemiology in Hamburg, Germany. Data are protected against unauthorised access at any time. While handling your data, the principles of the data protection law will be followed. For details, especially regarding the possibility to withdraw your consent, please refer to the informed consent form at the end of this information.

13. Whom should I contact in case of further questions?

Should you wish to receive further information about this clinical trial, or should you have any questions regarding your treatment and your rights as a subject in this study or regarding a damage to your health that may occur during this treatment, please contact your study doctor mentioned on page 1 of this document or any other doctor.

Informed Consent

Early treatment of Atrial fibrillation for Stroke prevention Trial
(EAST study; EudraCT No. 2010-021258-20)

I have been informed by _________________________________ (*name of the doctor conducting informed consent discussion*) to my satisfaction about the content, procedures, risks, and objectives of the above mentioned clinical trial. I have been given opportunity to ask questions and my questions have been answered. I have had sufficient time to make a clear decision whether or not to participate in this clinical trial. In addition, I have read and understood the written patient information sheet and the following data protection consent. I have received a copy of the patient information sheet.

I know that my participation is voluntary and that I can withdraw my consent at any time without giving any reason. This withdrawal will not affect my further treatment.

Data Protection Consent:

I understood that personal data regarding my medical history will be collected, stored and analysed within the context of this clinical trial. Data regarding my medical history will be used in accordance with legal requirements; a prerequisite for the use of my data is the voluntarily issued informed consent as detailed below. Without the following consent I cannot participate in this clinical trial.

1. I agree that the study-related data regarding my medical history and my treatment are collected and recorded - either on paper or electronically - at the study site mentioned on page 1 of this document. As far as necessary, these data may be forwarded in pseudonymous form (i.e. coded so that the relation of the medical data to my person is no longer possible) to:
   a) The Clinical Research Institute (CRI) in Munich, Germany, for the purpose of data processing,
   b) the institute for medical biometrics and epidemiology in Hamburg, Germany, for the purpose of statistical analyses,
   c) in case of adverse events: to the competent Ethics Committee and the competent national authority and further to the European database of clinical trials.
2. I further agree that authorised persons of the sponsor or of the CRI (so-called "monitors" and "auditors") or members of staff of the competent regulatory authorities or federal health authorities (European Medicines Evaluation Agency, as well as other foreign regulatory authorities) are entitled to view my personal data available at my study doctor's site, to such extent as is necessary for the proper conduct of this clinical trial. These persons are bound to confidentiality. For this process, I release the study doctor from his duty to maintain confidentiality.
3. I agree that my study-related data will be archived for at least 10 years after completion or premature termination of this clinical trial. Thereafter my personal data will be erased unless this does conflict with e.g. different legal or contractual periods of record-keeping.
   Should I decide to withdraw from this clinical trial prematurely, my data collected up to this point in time will not be erased immediately, but only after the above mentioned standard periods of time, for legal reasons.
4. I agree that my family doctor / treating physician

   ....
   (name)

   will be informed regarding my participation in this clinical trial (please delete if not welcome).

I hereby give my consent to participate voluntarily in this clinical trial.

To be completed ***by the patient personally***

________________________________ ____________ ____________________

First name and surname (printed) Date Signature

I have conducted the informed consent discussion and obtained the patient's informed consent.

To be completed personally ***by the doctor conducting*** *informed consent discussion*

Patient-ID |__|__|__|__| ____________ ____________________

Date Signature

Informed Consent
regarding the analysis of human blood

Early treatment of Atrial fibrillation for Stroke prevention Trial
(EAST study; EudraCT No. 2010-021258-20)

I have been informed by ________________________________ (*name of the doctor conducting informed consent discussion*) that a certain amount of blood shall be collected during the above mentioned clinical trial for scientific analyses. I have been given opportunity to ask questions and my questions have been answered.

I have been informed that the extra blood sample collected during the baseline examination will be sent to a central laboratory where it will be stored for up to 10 years after study completion. In this way it is possible to repeat measurements for verification purposes, if required, during this period of time. The purpose of these blood analyses is to gain new insights into how atrial fibrillation develops which may lead to improved treatments in the future. I have been informed that all these analyses will be done with pseudonymised (i.e. not labelled with my name but only with my study number) blood samples. I am aware that genetical analyses may be performed in these blood samples. These tests will be exclusively performed in relation to my heart problem.

I am assured that the blood sample collected will not be used for commercial purposes.

In addition, medical confidentiality will be maintained. All data protection provisions will be adhered to.

I agree to the collection, analysis, and pseudonymised (i.e. not labelled with my name but only with my study number) storage of the blood sample to be drawn during this clinical trial.

To be completed ***by the patient personally***

________________________________ ____________ ____________________

First name and surname (printed) Date Signature

To be completed personally ***by the doctor conducting*** *informed consent discussion*

Patient-ID |__|__|__|__| ____________ ____________________

Date Signature

Appendix IX: Time Schedule

	Tasks	Date
Study planning	Draft Protocol, review and finalisation by Steering Committee	06/09-06/10
Study preparation	Set up of e-TMS (MARVIN), preparation of e-CRF; preparation of all other study relevant documentation	09/09-09/10
Study initiation	▪ Site selection, site contacts	10/09-12/10
	▪ Supply of the sites with study materials, initiation visits (country-wise)	03/11-12/11
	▪ Recruitment period (FPI to LPI)	07/11-12/16
	▪ Treatment / Follow-up of last patient (LPI to LPO)	01/17-06/19
Study duration	Mean follow-up period of all patients, assuming a linear patient recruitment	60 months
Interim analyses	▪ Blinded interim analysis for sample size recalculation	approx. 01/15 (or when 2,400 patients have been recruited)
	▪ 1st interim analysis (when the first 171 events in the 1st co-primary outcome have been observed)	approx. 01/15
	▪ 2nd interim analysis (when the first 343 events in the 1st co-primary outcome have been observed)	approx. 07/16
	▪ 3rd interim analysis (when the first 514 events in the 1st co-primary outcome have been observed)	approx. 12/19
Study closure	Final data cleaning /study closure	projected by end of 2021
Final analysis	Statistical analysis, incl. review by Steering Committee	projected by beginning of 2022

Appendix X: Definitions of study assessments

Atrial fibrillation (AF) is any arrhythmia that lasts longer than 30 seconds, is documented in an ECG and fulfils the ECG criteria of AF [8, 11], i.e. absolute irregularity of RR intervals without detectable P waves. Alternatively, AF is also present when atrial high-rate episodes (AHRE) are detected by pacemakers or implanted devices with intracardiac leads, provided that the total duration of AHRE is more than 5.5 hours in 30 days [42].

Recurrent AF is any symptomatic or asymptomatic AF episode (clinically lasting longer than 30 seconds) after successful index therapy that is documented in an ECG. When AF is only documented by a single telemetric ECG, verification of the presence of AF by another technique (standard ECG, Holter ECG or implanted ECG) is required. Any documentation of AF in a standard ECG or Holter ECG constitutes an AF recurrence.

Persistent AF is present when an AF episode either lasts longer than 7 days or is terminated by cardioversion (either with drugs or electrical shocks). Persistent AF also implies that a rhythm control therapy strategy is pursued. Persistent AF is discerned from long-lasting persistent AF by its duration and recurrence pattern.

Long-lasting persistent AF is persistent AF or recurrent AF that has been present for longer than 12 months. Usually, patients with long-lasting persistent AF have a history of unsuccessful rhythm control therapy attempts.

Permanent AF is continuous AF that is no longer deemed suitable for rhythm control therapy. Patients in permanent AF usually receive rate control therapy. The intensity of rate control therapy should follow accepted measurements including recognition of the possibility to perform “lenient rate control”[38].

Paroxysmal AF should be considered AF that lasts 7 days or less.

AF burden is defined as the time in AF per time of ECG monitoring. It is expressed as number of days with at least one documented AF-ECG / number of all days with at least one documented ECG.

Time of index therapy is the date of the first drug or device intervention after randomisation. This might be the first application of antiarrhythmic drug or the date of first AF ablation.

Time to recurrent AF is defined as the time from first documented sinus rhythm after initiation of index therapy to the first documented recurrent AF of any type.

Time to recurrent symptomatic AF is defined as the time from first documented sinus rhythm after initiation of index therapy to the first recurrent AF with accompanying AF-related symptoms.

Time to therapy change is defined as the time from initiation of index therapy to the first change of AF related therapy. Change in therapy may consist of initiation of antiarrhythmic drug therapy, catheter ablation of AF, or a change from one antiarrhythmic drug to another, among others (e.g. adjustment of pacemaker or ICD). Repetitive pulmonary vein isolation, adaptation of drug doses, or re-programming of intracardiac devices is not considered therapy change in this context. Addition of a new antiarrhythmic drug or change from one drug to another, however, is considered therapy change. For the purpose of this outcome, ß adrenoreceptor blockers are not considered antiarrhythmic drugs. Re-ablation employing other ablation techniques (CFAE, linear lesions) also constitutes therapy change.

AF-related symptoms are symptoms that are considered to be related to AF. The items palpitations, fatigue, dizziness, dyspnoea, chest pain, and anxiety will be assessed by the treating physician to determine an overall symptom score (EHRA score [27]) as specified below:

EHRA I	no symptoms
EHRA II	mild symptoms, daily activity not affected
EHRA III	severe symptoms, normal daily activity affected
EHRA IV	disabling symptoms, normal daily activity discontinued

A very similar score has been validated by the Canadian Cardiovascular Society [39]. In addition, the EHRA score quantifies the frequency of symptoms as occasional (less than once per month), intermediate (once per month—almost daily), and frequent (at least daily).

Death. Death is defined as the irreversible collapse of the cardiovascular, central nervous and respiratory system. All deaths will be recorded.

Cardiovascular death is defined as any death due to cardiovascular reason (e.g. myocardial infarction, cardiogenic shock, arrhythmia/ sudden death, cardiac rupture, stroke, pulmonary embolism). It will be sub-divided into cardiac arrhythmic death, cardiac non-arrhythmic death and vascular non-cardiac death (incl. stroke). If no other cause is identified, cardiovascular death will be assumed [27] (worst case scenario).

AF-related death. All cardiovascular deaths that do not have a clearly defined other cause (e.g. rupture of an aneurysm, pulmonary embolism, cardiac tamponade, or myocardial infarction, among others) are classified as AF-related death when AF was present in the seven days prior to death [27]. All deaths that are an outcome of AF-related treatment are also counted as AF-related deaths.

Stroke. Any focal neurological deficit must immediately raise the suspicion of stroke. Stroke is defined as a new neurological deficit with a corresponding new lesion on brain imaging, usually found by computed tomography or magnetic resonance imaging. Transient ischemic attacks with a matching lesion on imaging are part of this outcome parameter. Stroke comprises both ischemic and haemorrhagic stroke [27].

All-cause hospitalizations comprise all hospitalizations with at least one over-night stay in the hospital. Hospitalizations will be quantified by the number of admissions and by nights in hospital. Furthermore, type and number of interventions during hospitalization will be recorded (see below). All hospitalisations will be characterized as planned or unplanned.

Cardiovascular hospitalizations comprise all hospitalizations requiring at least one overnight stay in the hospital for cardiovascular reasons excluding AF, acute coronary syndrome and worsening of heart failure (see definition below). The pre-specified main causes for cardiovascular hospitalisation are:

- Stable angina pectoris or atypical chest pain,
- syncope,
- TIA or stroke (including intracranial haemorrhage),
- non-fatal cardiac arrest,
- ventricular arrhythmia,
- cardiac transplantation,
- any type of cardiovascular surgery,
- implantation of a pacemaker, ICD or any other cardiac device,
- percutaneous coronary, cerebrovascular or peripheral intervention,

- blood pressure related hospitalisation (hypotension, hypertension; except syncope),
- cardiovascular infection,
- major bleeding (see definition below),
- pulmonary embolism or deep vein thrombosis, and
- other cardiovascular reason.

Nights spent in hospital. For each overnight hospital stay, the nights spent in hospital will be counted and recorded. This includes all nights spent in hospital from randomization to end of follow-up.

Interventions and procedures. All procedures and interventions are counted as either cardiovascular or other interventions. Other interventions will be specified by the investigator (free text).

Cardiovascular interventions will comprise the following:

- Percutaneous coronary interventions (PCI),
- coronary artery bypass surgery,
- implantation of an ECG monitor device,
- heart valve surgery or percutaneous valve replacement or repair (e.g. TAVI, valvulotomy, etc),
- cardiac transplantation,
- implantation of a pacemaker, ICD or any other cardiac device,
- percutaneous cerebrovascular or peripheral intervention,
- electrical cardioversion,
- catheter ablation for AF,
- catheter ablation for other arrhythmias,
- cerebrovascular surgery or interventions for stroke or bleeding events (craniotomy, local thrombolysis, neurovascular interventions),
- vascular surgery (includes e.g. repair of aortic aneurysm, surgery for peripheral artery disease, pulmonary embolectomy, among others), and
- other interventions not mentioned beforehand.

Hospitalization for worsening of heart failure. Any hospitalization for new-onset shortness of breath or worsening of exercise capacity that severely limits daily activities should raise the suspicion of worsening heart failure. Worsening of heart failure should be confirmed by adequate clinical findings or measures, e.g. severe peripheral oedema, dyspnoea at rest, biomarkers such as brain natriuretic peptide, or demonstration of lung edema on chest radiograph or worsening of left ventricular function, or by use of iv diuretics. Hospitalizations for acute cardiac decompensation due to AF are part of this outcome parameter.

Hospitalization for acute coronary syndrome. Any hospitalization that is due to new-onset or worsening chest pain is considered as an acute coronary syndrome when myocardial ischemia or coronary heart disease requiring therapy are found upon admission. Objective signs may consist of significant coronary stenosis upon angiography (usually requiring intervention), demonstration of acute ischemia by electrocardiogram or stress test (e.g. stress echocardiography, nuclear methods, or cardiac magnetic resonance imaging), or elevated cardiac biomarkers such as troponin I, troponin T, and/or creatine kinase with a cardiac origin. This outcome parameter comprises all myocardial infarctions (STEMI or NSTEMI [28]).

Hospitalization for AF. Any hospitalization for treatment of AF with at least one overnight stay. This may consist of hospitalizations for cardioversion, initiation of antiarrhythmic drug therapy, or catheter ablation of AF, among others.

Left ventricular function will be measured by M-mode echocardiography as left ventricular end-diastolic and end-systolic diameter, and fractional shortening. These mandatory measurements may be supplemented by left ventricular end-diastolic and end-systolic volume as estimated by the Simpson (biplane) method. For patients in AF, both M-mode and 2-D measurements will be averaged over 5 cardiac cycles.

Left atrial size will be assessed in a subset of patients by three-dimensional echocardiography (systolic and diastolic volumetry). In all patients, left atrial diameter at the aortic root will be determined by M-mode echocardiography.

Major bleeding is defined as a bleeding event that results in death (also part of the outcome "death"), requires surgical or interventional therapy, requires transfusion of 2 or more units of red blood cells, or results in permanent organ damage. Symptomatic bleeding in a critical area, such as intracranial, intraspinal, intraocular, retroperitoneal, intraarticular or pericardial, or intramuscular with compartment syndrome, is also considered as a major bleeding. This definition of bleeds is modified from a suggestion put forward by the International Society for Thrombosis and Hemostasis [29]. Intracranial bleeds may also be part of the outcome parameter "stroke" when they qualify as stroke by clinical criteria.

Minor bleeding is defined as any clinically important bleeding that did not qualify as major; for example, epistaxis, ecchymosis, hematoma, or macroscopic hematuria.

Myocardial infarction is defined as an acute episode of chest pain or myocardial ischemia when any of the following criteria are fulfilled (according to the consensus document of the Joint ESC/ACCF/AHA/WHF Task Force for the Redefinition of Myocardial Infarction, 2007):

- Detection of rise and/or fall of cardiac biomarkers (preferably troponin) with at least one value above the 99th percentile of the upper reference limit (URL) together with evidence of myocardial ischemia with at least one of the following:
  - Symptoms of ischemia,
  - ECG changes indicative of new ischemia (new ST-T changes or new left bundle branch block [LBBB]),
  - Development of pathological Q waves in the ECG,
  - Imaging evidence of new loss of viable myocardium or new regional wall motion abnormality.
- Sudden, unexpected cardiac death, involving cardiac arrest, often with symptoms suggestive of myocardial ischemia, and accompanied by presumably new ST elevation, or new LBBB, and/or evidence of fresh thrombus by coronary angiography and/or at autopsy, but death occurring before blood samples could be obtained, or at a time before the appearance of cardiac biomarkers in the blood.
- For percutaneous coronary interventions (PCI) in patients with normal baseline troponin values, elevations of cardiac biomarkers above the 99th percentile URL are indicative of peri-procedural myocardial necrosis. By convention, increases of biomarkers greater than 3 x 99th percentile URL have been designated as defining PCI-related myocardial infarction. A subtype related to a documented stent thrombosis is recognized.
- For coronary artery bypass grafting (CABG) in patients with normal baseline troponin values, elevations of cardiac biomarkers above the 99th percentile URL are indicative of peri-procedural myocardial necrosis. By convention, increases of biomarkers greater than 5 x 99th percentile URL plus either new pathological Q waves or new LBBB, or angiographically documented new graft or native coronary artery occlusion, or imaging

evidence of new loss of viable myocardium have been designated as defining CABG-related myocardial infarction.
- Pathologic findings of an acute myocardial infarction.

Cost of therapy will be calculated based on medication, time spent in hospital, procedures, assistance required, and loss of working capability.

Stable heart failure indicates that there is heart failure demonstrated by a reduced systolic left ventricular function or by symptoms of heart failure (which includes heart failure with preserved ejection fraction, so-called diastolic heart failure). “Stable” indicates that the patient is in a stable condition, i.e. there are no symptoms of heart failure at rest (patient is not in NYHA IV). Furthermore, “stable heart failure” excludes a recent decompensation of heart failure. After acute decompensation, patients can be included if they have remained stable for at least 4 weeks after discharge from hospital for management of acute decompensation.

Appendix XI: Design and sample size calculation

Assumptions (based on published outcome rates extracted from several published trials and refined by analyses in the ATHENA data base (unpublished analyses)

- **Control group: Event rate per year: 8%** (Comment: This corresponds to a monthly hazard rate of 0.0069485)
- **Test Group: 20% hazard rate reduction**.
- The power of the 2nd co-primary outcome is substantially larger than the power of the 1st co-primary outcome.

Key determinations:

- **Alpha: 5%** two-sided
- Alpha spent for the 1st co-primary outcome: 4% two-sided
- Alpha spent for the 2nd co-primary outcome: 1% two-sided
- Power 1st co-primary outcome: 80%
- The adaptive design will be defined by the 1st co-primary outcome only since it is expected to be the less powerful of the two co-primary outcomes. Thus, the power of the 2nd co-primary outcome cannot be defined but is a result of the total duration of the trial and yet unknown parameters.
- Duration of recruitment: 65 months
- **Follow-up time: at least 30 months** (Comment: A total trial duration of approximately 95 months or 8 years results)
- **Loss-to-follow-up: 5% of observation time**
- Blinded interim analysis for sample size re-calculation/adaptation of study design after 42 months or when 2,400 patients have been included, whatever is first.

Design:

- Three unblinded interim analyses for early determination for significance when 25%, 50% or 75% of the required amount of information on the 1st co-primary outcome was collected
- Use of the O'Brien-Fleming alpha spending function

Sample size calculation

With these assumptions and determinations and a loss-to-follow-up of 5% of the observation time, a total of 2,745 patients have to be randomized over 65 months **at a rate of 42 patients/month**.

The first interim analysis is to be performed when the first 171 events in the 1st co-primary outcome have been observed. If the assumptions are met, this will happen 42 months after the first patient was randomized. This analysis will be performed at a significance level of 0.00000328 (reject bound 4.508). The power will be 0.12%.

The second interim analysis is to be performed when the first 343 events in the 1st co-primary outcome have been observed. If the assumptions are met, this will happen 61 months after the first patient was randomized. This analysis will be performed at a significance level of 0.0010 (reject bound 3.090). The power will be 15%.

The third interim analysis is to be performed when the first 514 events have been observed. If the assumptions are met, this will happen 77 (11) months after the first (last) patient was randomized. This analysis will be performed at a significance level of 0.0069 (reject bound 2.462). The power spent so far will be 53%.

The final analysis is to be performed when 685 events have been observed. If the assumptions are met, this will happen 95 (30) months after the first (last) patient was randomized. This analysis will be performed at a significance level of 0.0177 (reject bound 2.103), resulting in a total alpha spent of 4%. The total power spent will be 80%.

Calculations were done with software ADDPLAN 5.0, Wassmer/Lehmacher, Cologne, Germany, based on the Schoenfeld formula.

Appendix XII: MoCA test

Only sample – the form to be used throughout the study will be made available as a separate document.

MONTREAL COGNITIVE ASSESSMENT (MOCA)

NAME :
Education :
Sex :
Date of birth :
DATE :

VISUOSPATIAL / EXECUTIVE | POINTS

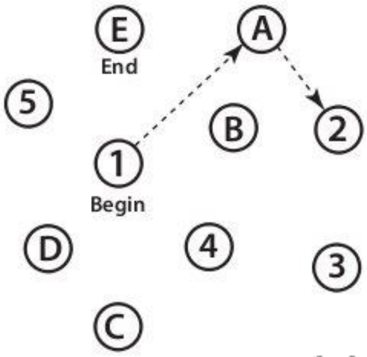

[ ]

Copy cube

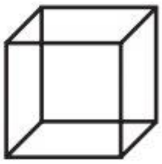

[ ]

Draw CLOCK (Ten past eleven)
(3 points)

[ ] Contour [ ] Numbers [ ] Hands ___/5

NAMING

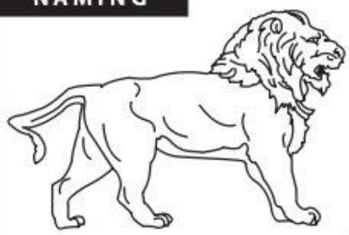

[ ]

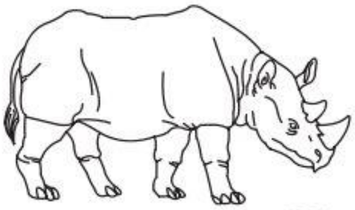

[ ]

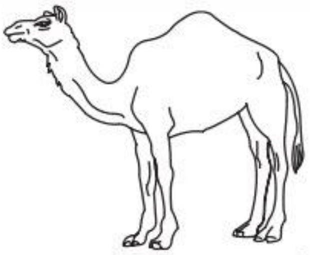

[ ] ___/3

MEMORY Read list of words, subject must repeat them. Do 2 trials, even if 1st trial is successful. Do a recall after 5 minutes.

	FACE	VELVET	CHURCH	DAISY	RED
1st trial					
2nd trial					

No points

ATTENTION Read list of digits (1 digit/ sec.). Subject has to repeat them in the forward order [ ] 2 1 8 5 4
Subject has to repeat them in the backward order [ ] 7 4 2 ___/2

Read list of letters. The subject must tap with his hand at each letter A. No points if ≥ 2 errors
[ ] F B A C M N A A J K L B A F A K D E A A A J A M O F A A B ___/1

Serial 7 subtraction starting at 100 [ ] 93 [ ] 86 [ ] 79 [ ] 72 [ ] 65
4 or 5 correct subtractions: **3 pts**, 2 or 3 correct: **2 pts**, 1 correct: **1 pt**, 0 correct: **0 pt** ___/3

LANGUAGE Repeat : I only know that John is the one to help today. [ ]
The cat always hid under the couch when dogs were in the room. [ ] ___/2

Fluency / Name maximum number of words in one minute that begin with the letter F [ ] _____ (N ≥ 11 words) ___/1

ABSTRACTION Similarity between e.g. banana - orange = fruit [ ] train – bicycle [ ] watch - ruler ___/2

**DELAYED RECALL**	Has to recall words **WITH NO CUE**	FACE [ ]	VELVET [ ]	CHURCH [ ]	DAISY [ ]	RED [ ]	Points for UNCUED recall only	___/5
**Optional**	Category cue							
	Multiple choice cue							

ORIENTATION [ ] Date [ ] Month [ ] Year [ ] Day [ ] Place [ ] City ___/6

© Z.Nasreddine MD Version 7.1 **www.mocatest.org** Normal ≥26 / 30 TOTAL ___/30

Administered by:_______________________ Add 1 point if ≤ 12 yr edu

Appendix XIII: EQ-5D questionnaire

Only sample – the form to be used throughout the study will be made available as a separate document.

By placing a tick in one box in each group below, please indicate which statements best describe your own health state today.

Mobility

I have no problems in walking about ❑

I have some problems in walking about ❑

I am confined to bed ❑

Self-Care

I have no problems with self-care ❑

I have some problems washing or dressing myself ❑

I am unable to wash or dress myself ❑

Usual Activities (e.g. work, study, housework, family or leisure activities)

I have no problems with performing my usual activities ❑

I have some problems with performing my usual activities ❑

I am unable to perform my usual activities ❑

Pain/Discomfort

I have no pain or discomfort ❑

I have moderate pain or discomfort ❑

I have extreme pain or discomfort ❑

Anxiety/Depression

I am not anxious or depressed ❑

I am moderately anxious or depressed ❑

I am extremely anxious or depressed ❑

© EuroQoL Group 1990

Best imaginable health state

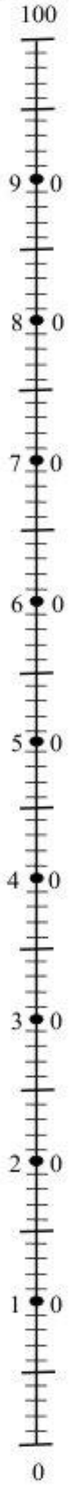

Worst imaginable health state

To help people say how good or bad a health state is, we have drawn a scale (rather like a thermometer) on which the best state you can imagine is marked 100 and the worst state you can imagine is marked 0.

We would like you to indicate on this scale how good or bad your own health is today, in your opinion. Please do this by drawing a line from the box below to whichever point on the scale indicates how good or bad your health state is today.

Your own health state today

© EuroQoL Group 1990

Appendix XIV: SF-12

Only sample – the form to be used throughout the study will be made available as a separate document.

1) In general, would you say your health is:

Excellent	Very good	Good	Fair	Poor
○	○	○	○	○

2) The following questions are about activities you might do during a typical day. Does <u>your health now limit</u> you in these activities? If so, how much?

	Yes, limited a lot	Yes, limited a little	No, not limited at all
a. <u>Moderate activities</u>, such as moving a table, pushing a vacuum cleaner, bowling, or playing golf	○	○	○
b. Climbing <u>several</u> flights of stairs	○	○	○

3) During the <u>past 4 weeks</u>, how much of the time have you had any of the following problems with your work or other regular daily activities <u>as a result of your physical health</u>?

	All of the time	Most of the time	Some of the time	A little of the time	None of the time
a. <u>Accomplished less</u> than you would like	○	○	○	○	○
b. Were limited in the <u>kind</u> of work or other activities	○	○	○	○	○

4) During the <u>past 4 weeks</u>, how much of the time have you had any of the following problems with your work or other regular daily activities <u>as a result of any emotional problems</u> (such as feeling depressed or anxious)?

	All of the time	Most of the time	Some of the time	A little of the time	None of the time
a. <u>Accomplished less</u> than you would like	○	○	○	○	○
b. Did work or activities <u>less carefully than usual</u>	○	○	○	○	○

5) During the <u>past 4 weeks</u>, how much did <u>pain</u> interfere with your normal work (including both work outside the home and housework)?

Not at all	A little bit	Moderately	Quite a bit	Extremely
○	○	○	○	○

6) These questions are about how you feel and how things have been with you <u>during the past 4 weeks</u>. For each question, please give the one answer that comes closest to the way you have been feeling. How much of the time during the <u>past 4 weeks</u>...

	All of the time	Most of the time	Some of the time	A little of the time	None of the time
a. Have you felt calm and peaceful?	○	○	○	○	○
b. Did you have a lot of energy?	○	○	○	○	○
c. Have you felt downhearted and depressed?	○	○	○	○	○

7) During the <u>past 4 weeks</u>, how much of the time has your <u>physical health or emotional problems</u> interfered with your social activities (like visiting friends, relatives, etc.)?

All of the time	Most of the time	Some of the time	A little of the time	None of the time
○	○	○	○	○

SF-12® Health Survey Scoring Demonstration by QualityMetric Incorporated

Appendix XV: Description of ECG monitoring

Patients in the early therapy group will receive a patient-operated ECG monitoring device capable of transtelephonic transmission (Tele-ECG). This device will allow to detect asymptomatic AF recurrences. Every patient receives a personal credit card-sized, single-lead event recorder (Tele-EKG-Card 100 IR Vitaphone®, Germany) to record his ECG 2-3 times per week as well as to transmit the data via telephone. Several technical prerequisites had to be fulfilled: simple and safe handling by the patient, easy transtelephonic transmission of the ECG to the analysis centre, sufficient ECG quality to assess P waves, fully automatic ECG reception 24 hours a day and automated first-line rhythm analysis separating sinus rhythm ECGs from suspected rhythm disturbances. The transmitted Tele-ECG is the basis for early recognition of recurrent AF. The ECG is obtained as a single-lead recording

- and then stored in digital format,
- sent as a frequency-modulated audio signal via telephone to the receiving centre,
- and automatically first-line analysed for rhythm disturbances.

The ECG is recorded using 4 gold electrodes, which are integrated into the backside of the Tele-ECG device. The device is safe and easy to use. Earlier trials have shown that placement of the device on the sternum and pushing a button to begin recording cause no problems even to older patients. A loud beeping sound indicates that ECG recording is performed. A tone signal with a frequency of 2,000 Hz is modulated by the amplitudes of the ECG signal. With optimal skin contact (= interference-free ECG recording), the sound is synchronized with pulse rate; poor skin contact results in squeaky noise, thereby indicating poor recording conditions. After a few tries, the patient is able to control the quality of the ECG recording by listening to the sound of the ECG signal. ECG recording automatically stops after 30 seconds. The resulting frequency-modulated signal is stored digitally in a solid state memory. Up to three ECG recordings can be stored, thereafter a transmission of the ECGs is necessary to allow for new recordings.

The stored ECGs can be sent using regular telephones including mobile phones. Several receiving units are available at the analysis centre. Automated processing of the transmitted ECG data from the individual patient is preserved. The telephonic ECG transfer is simple. After pushing a send-button, the patient positions the Tele-ECG loudspeaker to the telephone. The Tele-ECG device emits a short sequence of audio signals containing coded information about the device identification number, date and time of the ECG-recording as well as about the charge status of the batteries. Subsequently the stored ECG is send as a frequency-modulated audio signal. The receiving station decodes and transfers the audio signal to an amplitude-modulated time series which is digitally stored. After ECG transmission the identified serial number of the device is announced to the patient. In case of discrepancies of the announced number compared to the Tele–ECG serial number printed on the device the patient is asked to call his investigator.

The receiving units are connected to a local computer network. Incoming ECGs will be digitally stored on the server and can be retrieved by authorized workstations. The stored ECGs will be integrated into the trial data base for further pseudonymous analyses.

All Tele-ECGs will be classified automatically as a) sinus rhythm, b) suspicious for atrial fibrillation, c) suspicious for other rhythm disturbances or d) not valid for automated classification. In case of a), the ECG will be archived but not manually evaluated. In case of b) and c), the ECG recording will be sent automatically as PDF to the corresponding study site for further evaluation. In case of d), the ECG will be manually classified by CRI into a), b) or c), or remain in d).

All study sites receive monthly status reports of the Tele-ECG monitoring of their patients. This information allows for individual patient instruction and motivation at the visits.

Appendix XVI: Declaration of Helsinki (Version Seoul, October 2008)

(separate document)

Appendix XVII: Consensus statement of HRS/EHRA for catheter ablation of atrial fibrillation (Calkins et al Europace 2007).

(separate document)

Appendix XVIII: ESC 2012 guidelines for the management of patients with atrial fibrillation

(Camm AJ, Kirchhof P, Lip G, Schotten U, Savelieva I, Ernst S, van Gelder IC, Al-Attar N, Hindricks G, Prendergast B, Heidbuchel H, Alfieri O, Angelini A, Atar D, Colonna P, De Caterina R, De Sutter J, Goette A, Gorenek B, Heldal M, Hohnloser S, Kolh P, Le Heuzey J, Ponikowski P, Rutten F. ESC Guidelines for the management of patients with atrial fibrillation. Eur Heart J 2012)

(separate document)

Appendix XIX: Karnofsky score

(Karnofsky DA, Burchenal JH. (1949). "The Clinical Evaluation of Chemotherapeutic Agents in Cancer." In: MacLeod CM (Ed), Evaluation of Chemotherapeutic Agents. Columbia Univ Press. Page 196)

In medicine (oncology and other fields), performance status is an attempt to quantify patients' general well-being and activities of daily life

The Karnofsky score runs from 100 to 0, where 100 is "perfect" health and 0 is death. Although practitioners occasionally assign performance scores in between standard intervals of 10, there is no substantiated rationale for this and prognostication is not improved. This scoring system is named after Dr David A. Karnofsky, who described the scale with Dr Joseph H. Burchenal in 1949.

100% – normal, no complaints, no signs of disease

90% – capable of normal activity, few symptoms or signs of disease

80% – normal activity with some difficulty, some symptoms or signs

70% – caring for self, not capable of normal activity or work

60% – requiring some help, can take care of most personal requirements

50% – requires help often, requires frequent medical care

40% – disabled, requires special care and help

30% – severely disabled, hospital admission indicated but no risk of death

20% – very ill, urgently requiring admission, requires supportive measures or treatment

10% – moribund, rapidly progressive fatal disease processes

0% – death
