# Supplementary material for: Diabetes and Obesity and Treatment Effect of Early Rhythm Control vs Usual Care in Patients With Atrial Fibrillation: A Secondary Analysis of the EAST-AFNET 4 Randomized Clinical Trial
Source: JAMA Cardiol. 2025 Jul 30;10(9):932–41. doi: 10.1001/jamacardio.2025.2374 (PMC12311819; doi:10.1001/jamacardio.2025.2374)
Supplement: Supplement 2. — eTable 1. Safety Outcomes for BMI Categories eTable 2. Safety Outcomes for ERC and UC for Patients With and Without Diabetes eFigure 1. Rhythm Control Chosen by Treatment Group for Patients With a BMI <30 kg/m2 and With a BMI ≥30 kg/m2 eFigure 2. Rhythm Control Chosen by Treatment Group for Diabetes and for Non-Diabetes Patients eFigure 3. CONSORT Checklist eAppendix. Links to Study Protocol and Statistical Analysis Plan [file jamacardiol-e252374-s002.pdf]

Supplemental Online Content

Metzner A, Willems S, Borof K, et al. Diabetes and obesity and treatment effect of early rhythm control vs usual care in atrial fibrillation: a secondary analysis of the EAST-AFNET4 randomized clinical trial. *JAMA Cardiol*. Published online July 30, 2025. doi:10.1001/jamacardio.2025.2374

eTable 1. Safety Outcomes for BMI Categories
eTable 2. Safety Outcomes for ERC and UC for Patients With and Without Diabetes
eFigure 1. Rhythm Control Chosen by Treatment Group for Patients With a BMI <30 kg/m^2 and With a BMI ≥30 kg/m^2
eFigure 2. Rhythm Control Chosen by Treatment Group for Diabetes and for Non-Diabetes Patients
eFigure 3. CONSORT Checklist
eAppendix. Links to Study Protocol and Statistical Analysis Plan

This supplemental material has been provided by the authors to give readers additional information about their work.

© 2025 Metzner A et al. *JAMA Cardiology*.

eTable 1. Safety Outcomes for BMI Categories

	Underweight-Pre-Obesity		Obesity class I-III			
	Early rhythm control	Usual care	Early rhythm control	Usual care	p-value interaction	pooled p-value
n	866	824	522	564		
Primary composite safety outcome	160 (18.5)	140 (17.0)	70 (13.4)	82 (14.5)	0.368	0.011
Stroke	34 (3.9)	42 (5.1)	6 (1.1)	19 (3.4)	0.127	0.002
Death	97 (11.2)	104 (12.6)	41 (7.9)	60 (10.6)	0.427	0.031
Serious adverse event of special interest related to rhythm control therapy	39 (4.5)	10 (1.2)	28 (5.4)	9 (1.6)	0.86	0.339
Serious adverse event related to antiarrhythmic drug therapy						
Nonfatal cardiac arrest	1 (0.1)	1 (0.1)	0 (0.0)	0 (0.0)	>0.99	>0.99
Drug toxicity of AF related drug therapy	8 (0.9)	1 (0.1)	2 (0.4)	2 (0.4)	0.179	0.577
Drug induced bradycardia	6 (0.7)	2 (0.2)	8 (1.5)	3 (0.5)	0.989	0.087
Atrioventricular block	2 (0.2)	0 (0.0)	0 (0.0)	0 (0.0)	0.511	0.223
Torsade de pointes tachycardia	0 (0.0)	0 (0.0)	1 (0.2)	0 (0.0)		
Serious adverse event related to AF ablation						
Pericardial tamponade	2 (0.2)	0 (0.0)	1 (0.2)	0 (0.0)	0.993	0.879
Major bleeding related to AF ablation	2 (0.2)	0 (0.0)	4 (0.8)	0 (0.0)	0.92	0.145
Nonmajor bleeding related to AF ablation	0 (0.0)	2 (0.2)	1 (0.2)	0 (0.0)	>0.99	0.946
Serious adverse event of special interest related to RC therapy						
Blood pressure related event	1 (0.1)	0 (0.0)	0 (0.0)	0 (0.0)		>0.99
Hospitalization for AF	7 (0.8)	2 (0.2)	3 (0.6)	1 (0.2)	0.976	0.628
Other cardiovascular event	2 (0.2)	1 (0.1)	3 (0.6)	0 (0.0)	>0.99	<0.001
Other event	0 (0.0)	2 (0.2)	1 (0.2)	1 (0.2)	0.763	0.737
Syncope	2 (0.2)	0 (0.0)	2 (0.4)	1 (0.2)		0.381
Hospitalization for worsening of HF with decomp HF	2 (0.2)	0 (0.0)	1 (0.2)	0 (0.0)		0.879
Implantation of a pacemaker defi or other	5 (0.6)	2 (0.2)	3 (0.6)	2 (0.4)	0.704	0.907

© 2025 Metzner A et al. *JAMA Cardiology*.

eTable 2. Safety Outcomes for ERC and UC for Patients With and Without Diabetes

	No Diabetes		Diabetes			
	Early rhythm control	Usual care	Early rhythm control	Usual care	p-value interaction	pooled p-value
n	1039	1051	351	343		
Primary composite safety outcome	167 (16.1)	162 (15.4)	64 (18.2)	61 (17.8)	0.985	0.217
Stroke	26 (2.5)	46 (4.4)	14 (4.0)	16 (4.7)	0.317	0.265
Death	97 (9.3)	113 (10.8)	41 (11.7)	51 (14.9)	0.686	0.033
Serious adverse event of special interest related to rhythm control therapy	55 (5.3)	17 (1.6)	13 (3.7)	2 (0.6)	<0.001	<0.001
**Serious adverse event related to antiarrhythmic drug therapy**						
Nonfatal cardiac arrest	0 (0.0)	0 (0.0)	1 (0.3)	1 (0.3)	0.875	0.776
Drug toxicity of AF related drug therapy	8 (0.8)	3 (0.3)	2 (0.6)	0 (0.0)	0.699	0.425
Drug induced bradycardia	13 (1.3)	5 (0.5)	1 (0.3)	0 (0.0)	0.655	0.079
Atrioventricular block	2 (0.2)	0 (0.0)	0 (0.0)	0 (0.0)		>0.99
Torsade de pointes tachycardia	1 (0.1)	0 (0.0)	0 (0.0)	0 (0.0)	>0.99	0.977
**Serious adverse event related to AF ablation**						
Pericardial tamponade	3 (0.3)	0 (0.0)	0 (0.0)	0 (0.0)	>0.99	>0.99
Major bleeding related to AF ablation	3 (0.3)	0 (0.0)	3 (0.9)	0 (0.0)	>0.99	0.232
Nonmajor bleeding related to AF ablation	1 (0.1)	2 (0.2)	0 (0.0)	0 (0.0)	>0.99	0.638
**Serious adverse event of special interest related to RC therapy**						
Blood pressure related event	1 (0.1)	0 (0.0)	0 (0.0)	0 (0.0)	>0.99	>0.99
Hospitalization for AF	8 (0.8)	3 (0.3)	3 (0.9)	0 (0.0)	0.995	0.623
Other cardiovascular event	3 (0.3)	1 (0.1)	2 (0.6)	0 (0.0)	0.999	<0.001
Other event	0 (0.0)	2 (0.2)	1 (0.3)	1 (0.3)	0.725	0.288
Syncope	4 (0.4)	1 (0.1)	0 (0.0)	0 (0.0)	>0.99	0.94
Hospitalization for worsening of hf with decomp HF	3 (0.3)	0 (0.0)	0 (0.0)	0 (0.0)	>0.99	0.822
Implantation of a pacemaker defi or other	7 (0.7)	3 (0.3)	1 (0.3)	1 (0.3)	0.623	0.376

© 2025 Metzner A et al. *JAMA Cardiology*.

eFigure 1. Rhythm Control Chosen by Treatment Group for Patients With a BMI <30 kg/m^2 and With a BMI ≥30 kg/m^2

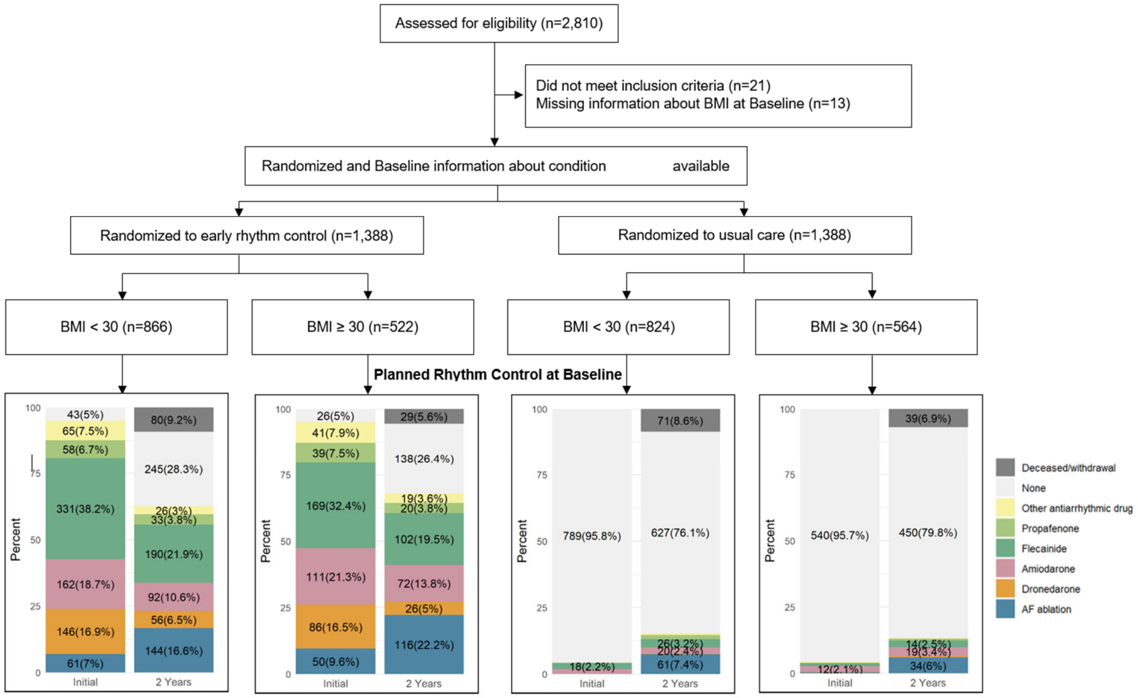

© 2025 Metzner A et al. *JAMA Cardiology*.

eFigure 2. Rhythm Control Chosen by Treatment Group for Diabetes and for Non-Diabetes Patients

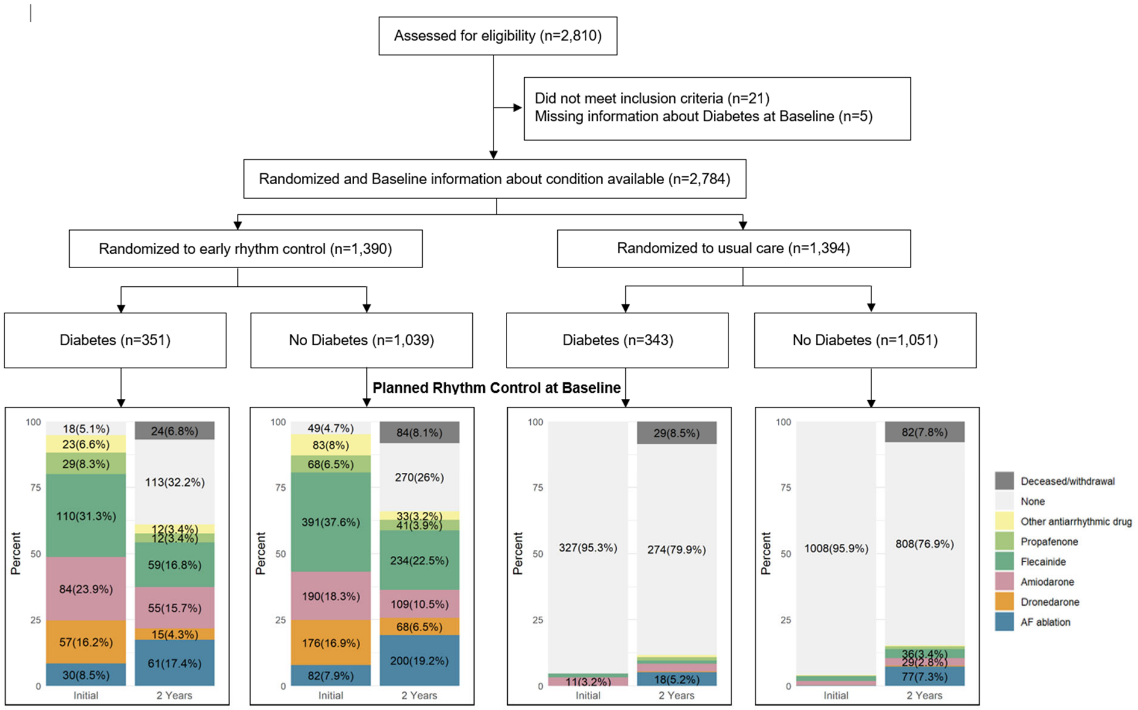

© 2025 Metzner A et al. *JAMA Cardiology*.

eFigure 3. CONSORT Checklist

CONSORT 2010 checklist of information to include when reporting a randomised trial*

Section/Topic	Item No	Checklist item	Reported on page No
**Title and abstract**			
	1a	Identification as a randomised trial in the title	yes
	1b	Structured summary of trial design, methods, results, and conclusions (for specific guidance see CONSORT for abstracts)	Suppl. Page 4
**Introduction**			
Background and objectives	2a	Scientific background and explanation of rationale	Abstract page 2
	2b	Specific objectives or hypotheses	Abstract page 2
**Methods**			
Trial design	3a	Description of trial design (such as parallel, factorial) including allocation ratio	Suppl. page 4
	3b	Important changes to methods after trial commencement (such as eligibility criteria), with reasons	none
Participants	4a	Eligibility criteria for participants	Suppl. page 4
	4b	Settings and locations where the data were collected	Suppl. page 4
Interventions	5	The interventions for each group with sufficient details to allow replication, including how and when they were actually administered	Suppl. page 4
Outcomes	6a	Completely defined pre-specified primary and secondary outcome measures, including how and when they were assessed	Methods page 3
	6b	Any changes to trial outcomes after the trial commenced, with reasons	none
Sample size	7a	How sample size was determined	Suppl. page 4
	7b	When applicable, explanation of any interim analyses and stopping guidelines	none
Randomisation:			
Sequence generation	8a	Method used to generate the random allocation sequence	Suppl. page 4
	8b	Type of randomisation; details of any restriction (such as blocking and block size)	Suppl. page 4
Allocation concealment mechanism	9	Mechanism used to implement the random allocation sequence (such as sequentially numbered containers), describing any steps taken to conceal the sequence until interventions were assigned	Suppl. page 4
Implementation	10	Who generated the random allocation sequence, who enrolled participants, and who assigned participants to interventions	Suppl. page 4
Blinding	11a	If done, who was blinded after assignment to interventions (for example, participants, care providers, those assessing outcomes) and how	none
	11b	If relevant, description of the similarity of interventions	
Statistical methods	12a	Statistical methods used to compare groups for primary and secondary outcomes	Statistics page 4
	12b	Methods for additional analyses, such as subgroup analyses and adjusted analyses	Statistics page 4
**Results**			
Participant flow (a diagram is strongly recommended)	13a	For each group, the numbers of participants who were randomly assigned, received intended treatment, and were analysed for the primary outcome	Suppl. table 1
	13b	For each group, losses and exclusions after randomisation, together with reasons	
Recruitment	14a	Dates defining the periods of recruitment and follow-up	Suppl. page 4
	14b	Why the trial ended or was stopped	Suppl. page 4
Baseline data	15	A table showing baseline demographic and clinical characteristics for each group	Suppl. table 1
Numbers analysed	16	For each group, number of participants (denominator) included in each analysis and whether the analysis was by original assigned groups	Suppl. table 1
Outcomes and estimation	17a	For each primary and secondary outcome, results for each group, and the estimated effect size and its precision (such as 95% confidence interval)	Tables 1,2,4,5
	17b	For binary outcomes, presentation of both absolute and relative effect sizes is recommended	
Ancillary analyses	18	Results of any other analyses performed, including subgroup analyses and adjusted analyses, distinguishing pre-specified from exploratory	
Harms	19	All important harms or unintended effects in each group (for specific guidance see CONSORT for harms)	none
**Discussion**			
Limitations	20	Trial limitations, addressing sources of potential bias, imprecision, and, if relevant, multiplicity of analyses	Page 12
Generalisability	21	Generalisability (external validity, applicability) of the trial findings	
Interpretation	22	Interpretation consistent with results, balancing benefits and harms, and considering other relevant evidence	Page 10
**Other information**			
Registration	23	Registration number and name of trial registry	Title page
Protocol	24	Where the full trial protocol can be accessed, if available	Suppl. page 4
Funding	25	Sources of funding and other support (such as supply of drugs), role of funders	Page 24

CONSORT 2010 checklist Page 1

*We strongly recommend reading this statement in conjunction with the CONSORT 2010 Explanation and Elaboration for important clarifications on all the items. If relevant, we also recommend reading CONSORT extensions for cluster randomised trials, non-inferiority and equivalence trials, non-pharmacological treatments, herbal interventions, and pragmatic trials. Additional extensions are forthcoming: for those and for up to date references relevant to this checklist, see www.consort-statement.org.

CONSORT 2010 checklist Page 2

© 2025 Metzner A et al. *JAMA Cardiology*.

eAppendix. Links to Study Protocol and Statistical Analysis Plan

Study protocol:
https://www.nejm.org/doi/suppl/10.1056/NEJMoa2019422/suppl_file/nejmoa2019422_protocol.pdf

Analysis plan:
https://www.nejm.org/doi/suppl/10.1056/NEJMoa2019422/suppl_file/nejmoa2019422_appendix.pdf

© 2025 Metzner A et al. *JAMA Cardiology*.
