# Supplementary material for: Diabetes and Obesity and Treatment Effect of Early Rhythm Control vs Usual Care in Patients With Atrial Fibrillation: A Secondary Analysis of the EAST-AFNET 4 Randomized Clinical Trial
Source: JAMA Cardiol. 2025 Jul 30;10(9):932–41. doi: 10.1001/jamacardio.2025.2374 (PMC12311819; doi:10.1001/jamacardio.2025.2374)
Supplement: Supplement 3. — Data Sharing Statement [file jamacardiol-e252374-s003.pdf]

Data Sharing Statement

Metzner. Diabetes and Obesity and Treatment Effect of Early Rhythm Control vs Usual Care in Patients With Atrial Fibrillation. *JAMA Cardiol*. Published July 30, 2025. doi:10.1001/jamacardio.2025.2374

Data
Additional Information: EudraCT: 2010-021258-20 ClinicalTrials.gov ID: NCT01288352 ISRCTN04708680
Data available: No

Additional Information
Explanation for why data not available: Data will be made available upon reasonable request. Requests to be sent to info@kompetenznetz-vorhofflimmern.de
